# The Complex Intron Landscape and Massive Intron Invasion in a Picoeukaryote Provides Insights into Intron Evolution

**DOI:** 10.1093/gbe/evt189

**Published:** 2013-11-20

**Authors:** Bram Verhelst, Yves Van de Peer, Pierre Rouzé

**Affiliations:** ^1^Department of Plant Biotechnology and Bioinformatics, Ghent University, Belgium; ^2^Department of Plant Systems Biology, VIB, Ghent, Belgium; ^3^Department of Genetics, Genomics Research Institute, University of Pretoria, South Africa

**Keywords:** intron evolution, intron gain, Mamiellophyceae, *Micromonas*, introner elements

## Abstract

Genes in pieces and spliceosomal introns are a landmark of eukaryotes, with intron invasion usually assumed to have happened early on in evolution. Here, we analyze the intron landscape of *Micromonas*, a unicellular green alga in the Mamiellophyceae lineage, demonstrating the coexistence of several classes of introns and the occurrence of recent massive intron invasion. This study focuses on two strains, CCMP1545 and RCC299, and their related individuals from ocean samplings, showing that they not only harbor different classes of introns depending on their location in the genome, as for other Mamiellophyceae, but also uniquely carry several classes of repeat introns. These introns, dubbed introner elements (IEs), are found at novel positions in genes and have conserved sequences, contrary to canonical introns. This IE invasion has a huge impact on the genome, doubling the number of introns in the CCMP1545 strain. We hypothesize that each IE class originated from a single ancestral IE that has been colonizing the genome after strain divergence by inserting copies of itself into genes by intron transposition, likely involving reverse splicing. Along with similar cases recently observed in other organisms, our observations in *Micromonas* strains shed a new light on the evolution of introns, suggesting that intron gain is more widespread than previously thought.

## Introduction

**Fig. 1.—:**
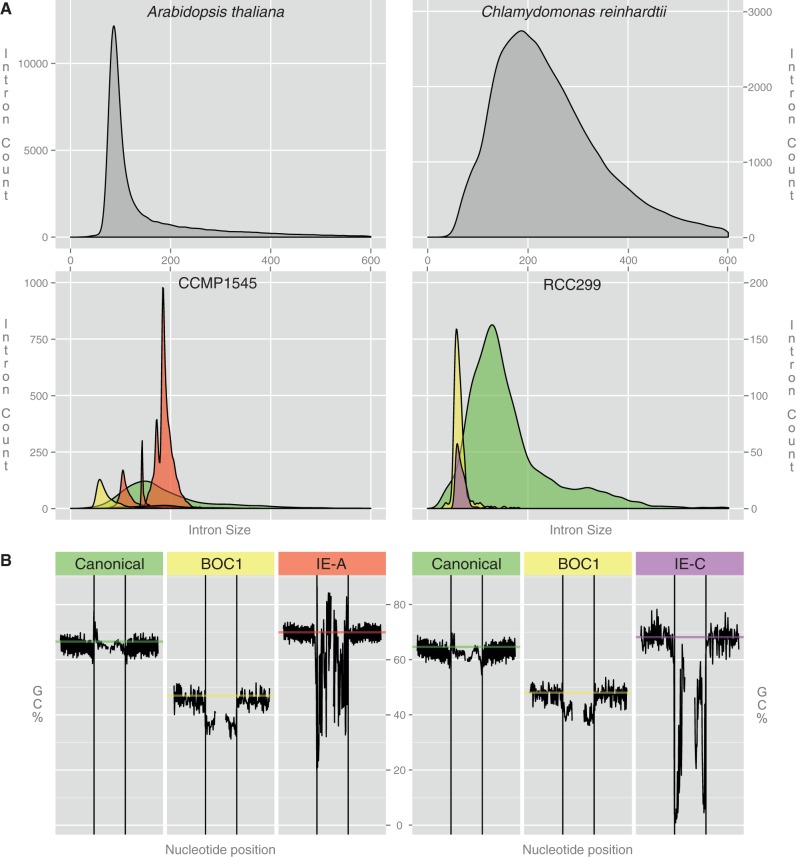
The intron landscape of *Micromonas*. (*A*) Size distribution of different intron classes in *Micromonas* strains CCMP1545 and RCC299 (intron sizes binned per 5 nt). The two panels at the top represent reference intron distributions for *Arabidopsis thaliana* and *Chlamydomonas reinhardtii*. The different classes are canonical (green), BOC1 (yellow), IE-A (red), and IE-C (purple). Due to their low occurrence, members of classes IE-B and IE-D are not displayed. Introns longer than 600 nt are excluded. (*B*) Average GC% of *Micromonas* introns (left: CCMP1545; right: RCC299) and their bordering exon regions. Exon/intron boundaries are marked by black vertical lines, while horizontal lines represent the average GC% of all coding sequences containing at least one intron of the specified class. Exons and introns were trimmed by 3 and 6 nt, respectively, on either end to omit splice-site signals. Only 80 (exon) and 40 (intron) nt on either side of the exon/intron boundary are displayed. Plots were drawn using ggplot2 (Wickham 2009).

Recently, several whole-genome sequences have been reported for Mamiellophyceae, eukaryotic picoalgae at the basis of the green lineage that play a major trophic role in the marine environment. Among these are the genome sequences of two *Micromonas* strains, isolated from tropical (Equatorial Pacific; strain RCC299) and coastal waters (Plymouth, English Channel; strain CCMP1545) (Worden et al. 2009). One striking outcome of the genome analysis of these algae was the observation of a complex intron landscape in *Micromonas*, especially in the CCMP1545 strain (fig. 1*A*). In common with other Mamiellophyceae, both *Micromonas* strains RCC299 and CCMP1545 feature two distinct classes of introns, corresponding to the unique genome heterogeneity of these picoalgae (Moreau et al. 2012). At most chromosomal locations, mamiellophycean genes harbor no or few canonical spliceosomal introns with conserved splice sites and branch-point motif (Derelle et al. 2006; Keeling 2007; Moreau et al. 2012). However, in all mamiellophycean genomes studied so far, two low-GC% regions can be identified that harbor peculiar introns (Derelle et al. 2006; Keeling 2007; Worden et al. 2009; Moreau et al. 2012). One of the low-GC% regions is located on a chromosome denoted as Big Outlier Chromosome (BOC) and is represented by chromosome 2 in CCMP1545 and chromosome 1 in RCC299. This BOC displays intron heterogeneity with numerous small AT-rich introns in the low-GC% region, dubbed BOC1 introns (Irimia and Roy 2008; Moreau et al. 2012) (fig. 1*B*). A small portion of these BOC1 introns feature noncanonical splice sites. Additionally, in *Micromonas* CCMP1545, Worden et al. (2009) reported the occurrence of repeat introns, dubbed introner elements (IEs). These IEs could be further subdivided into four different families (IE-A1–IE-A4) based on the presence or absence of specific IE sequence motifs and seemed to be absent from RCC299 or any other published mamiellophycean genome.

In this study, we present an in-depth analysis of these IEs and the discovery of three additional classes: IE-B and IE-D in CCMP1545 and IE-C in RCC299. All four classes show a high degree of within-class sequence conservation, are found on the sense strand of genes, follow similar genomic distribution patterns, and are found at unique positions in genes. These observations stand in sharp contrast to canonical spliceosomal introns, which generally display a very low degree of sequence conservation and are often found at conserved positions in genes. Based on the structural characteristics of IEs and the distribution of their occurrence, we propose that the mechanism by which they replicate possibly involves reverse splicing at the pre-mRNA level and conclude that the replication of IEs provides an important mechanism of intron gain. As a consequence, intron gain could be more widespread than commonly believed.

## Materials and Methods

### Sequence Data

*Micromonas* genome sequences (v2.0) as well as the Expressed Sequence Tag (EST) libraries were obtained from the JGI portal (http://genome.jgi-psf.org, last accessed November 28, 2013). Metagenomic sequences containing IEs were obtained (through BlastN) from the NCBI metagenomes database (taxid: 408169) and the CAMERA portal (Sun et al. 2011) using a handpicked set of ten IE sequences as query input. The CCMP1764 genome draft was assembled from the CAMERA CCMP1764 project data using the CLC Assembly Cell (v4.0.10; -b 110 –w 64).

*Arabidopsis thaliana* intron data were obtained from the TAIR10 intron database (v20101028), while *C. reinhardtii* intron data were derived from the latest Phytozome release (v5.3.1). When multiple isoforms were present, one representative was selected randomly.

### IE Prediction and Reannotation of *Micromonas* Genomes

IEs were predicted using a pattern matching approach, complemented with protein and EST evidence (supplementary methods, Supplementary Material online). Remnants of IEs were detected using a Blast (v2.2.17; -e 1e-3) and HMMer (v2.3.2) approach. Gene models were extensively curated through automated and manual procedures. All intron and gene information is stored in a relational database and can be accessed through the ORCAE platform (http://bioinformatics.psb.ugent.be/orcae, last accessed November 28, 2013) (Sterck et al. 2012). Data sets (gene models, intron sets, and environmental sequences) can be obtained from its download section.

### *Micromonas* Intron Classification: BOC1 and Canonical Introns

BOC1 introns are defined as short (<75 nt), AT-rich (<43 GC%) introns lying in the BOC1 region of chromosome 1 of CCMP1545 (position 438,300–2,118,000) and chromosome 2 of RCC299 (position 263,000–1,817,000) (Moreau et al. 2012). Canonical introns are defined as all remaining introns that do not fall in either the IE or BOC1 categories.

### Orthologous *Micromonas* Introns

In total, 6,891 one-to-one orthologous pairs were identified using orthoMCL (v2.0; mcl options: –abc –I 1.5), representing 74% of the total intron content of both *Micromonas* isolates. After alignment (MUSCLE v3.8.31; -diags), intron positions were compared and cross-referenced against their class identifier (IE-A, IE-B, IE-C, BOC1, canonical).

### Metagenomic Analysis

Metagenomic sequences were subjected to the IE prediction pipeline and aligned to the *Micromonas* genomes using a seed-and-align procedure (supplementary methods, Supplementary Material online). After quality filtering, we then compared IE positions to discover presence/absence polymorphisms (PAPs). This analysis is highly biased toward the finding of IEs that are absent in RCC299/CCMP1545 but present in the metagenomic sequences, as the reverse would require a confirmation that the read is derived from an organism that carries the specific IE. This is only the case when a sequence carries a strain identifier (i.e., as with the CCMP1764 case) or if the metagenomic sequence carries an IE up- or downstream.

## Results

*Micromonas* introns can be classified into two categories, namely singleton introns, which are all unique in the sense that they do not show significant similarity to other introns in the genome, and IEs, which are a copy of or at least show partial similarity to several or many other introns. To the first category (table 1) belong classes that are present in all Mamiellophyceae: the canonical introns and the BOC1 introns. The canonical spliceosomal introns of strains RCC299 and CCMP1545 favor the donor consensus sequence AG|GTGCGT (supplementary fig. S1, Supplementary Material online) and have a predicted NCTGAC branch-point motif at 43–52 bp upstream of the acceptor site. Comparative intron analysis revealed that 47% of all canonical intron positions are shared between CCMP1545 and RCC299 orthologs, a number that illustrates the divergence of these strains, which are members of different clades (RCC299: clade-II; CCMP1545: clade-V [Worden et al. 2009], fig. 3), and probably should be regarded as separate species.

**Table 1:** Micromonas Intron Properties

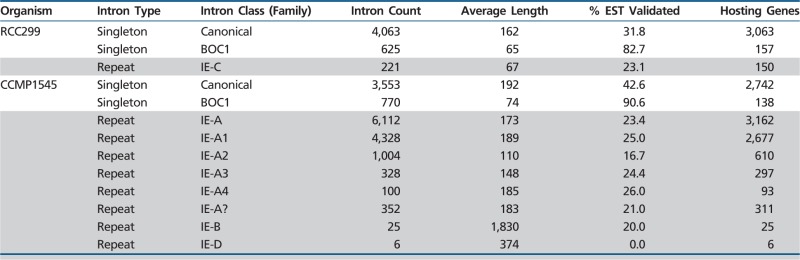

BOC1 introns share few common features, such as their short length and low GC% (fig. 1). The majority of BOC1 introns follow the common GT-AG splice site rule but have no discernible branch-point motif. Presumably, the drop in GC% (fig. 1*B*) across the splice site aids recognition by the splicing machinery. Furthermore, 34 of these introns feature noncanonical TG or CG acceptor sites, of which the majority is validated by EST alignments. Similar noncanonical acceptor sites have been found in non-prasinophytes as well (Denoeud et al. 2010). Most of the BOC1 intron positions (73%) are shared between both isolates. This percentage is considerably higher than the one for canonical introns (47%), which might be related to a constraint on the BOC1 genes, which are more highly expressed and more often functionally conserved (Moreau et al. 2012).

### Introner Elements

After careful analysis and reannotation of the *Micromonas* genomes, we have identified four distinct classes of IEs (table 1), that is, introns that are repeat elements in strains RCC299 and CCMP1545. These four IE classes differ in terms of host, abundance, sequence, and length. CCMP1545 contains three IE classes: IE-A, IE-B, and IE-D. IE-A has 6,112 members that can be further divided into four families of different size (IE-A1: 4,328; IE-A2: 1,004; IE-A3: 328; IE-A4: 100) and 352 elements with unclear class assignment due to the presence of insertions or deletions (indels) and sequence degeneracy. IE-A sequences consist of a series of sequence motifs, some of which are universal to all IE-A sequences and some of which are specific to one of the subclasses of IE-A (supplementary figs. S2–S5, Supplementary Material online). IE-A members also have very typical splice donor sites, AG|GYGCGT or AG|GTGAGAC, with the first occurring in IE-A1 and IE-A2, while the latter is almost exclusively found in IE-A3 and IE-A4 sequences (supplementary figs. S1–S5, Supplementary Material online). Fifty-three percent of IE-A1 sequences contain a GC splice donor, a characteristic that was noted in earlier studies but was never linked to the presence of IEs (Iwata and Gotoh 2011). Overall, IE-A dominates the intron landscape as it represents over half of all introns and is the main cause for the 1 Mb surplus in CCMP1545 genome size over RCC299.

Besides the IE-A introns, there are 463 IE-A-like repeats, which are positioned outside introns or inside preexisting introns (discussed later). These are remnants of IE-A introns: highly degenerated, partial copies that most often only consist of a small 50-nt motif (motif-C, supplementary figs. S2–S5, Supplementary Material online), having lost both splice sites and all other motifs crucial for the splicing process. They are found in close proximity to coding sequences (∼UTR regions) or within canonical intron sequences, but never in coding sequences where they are counter-selected for to maintain gene functionality.

The IE-B and IE-D class consist of 25 and 6 members, respectively, which have a very variable length, ranging from 100 up to 6,494 nt for certain IE-B members (supplementary fig. S6–S8, Supplementary Material online). Their GT-TG splice sites are highly unusual but have been reported before in other species, including human (Szafranski et al. 2007). Eight of the IE-B sequences harbor a long >3,000-nt open reading frame on the complementary strand (supplementary fig. S9, Supplementary Material online). This IE-encoded protein lacks homology to other known proteins, except for a small OTU-like protease domain. As such, the function of this protein, or the reason why it is embedded within these IEs, is unknown. The IE-B class contains both the longest documented mamiellophycean intron and the first documented occurrence of a nuclear intron-encoded protein within Mamiellophyceae. A defining characteristic of these two classes is the preference for phase-2 (i.e., the intron sits in between the second and third base of a codon), which contradicts the theory that newly gained introns prefer phase-0 (i.e., the intron sits in between two codons) (supplementary fig. S10, Supplementary Material online) (Nguyen et al. 2006). Although sharing common splice features, IE-Bs and IE-Ds do not show any sequence similarity, which is why they have been ascribed to different classes.

As stated previously, the IE-C class (221 occurrences) exists exclusively in RCC299. The IE-C sequences (with an average length of 67 nt) are much shorter than the IEs found in CCMP1545 and feature a highly conserved branch-point motif—GACTGACG—identical to the extended branch-point sequence reported for canonical *Ostreococcus* introns (Irimia and Roy 2008) (supplementary fig. S11, Supplementary Material online).

IEs, present in a third of all CCMP1545 genes, are fully functional spliceosomal introns. Beside the fact that they feature the necessary splicing-related motifs (donor and acceptor sites, branch-point, poly-Y tract), EST evidence confirms their excision from primary transcripts. Even more, the nonexcision of IEs from the transcripts would generally lead to a premature stop codon resulting in nonsense-mediated mRNA decay (Jaillon et al. 2008).

### Genomic Localization

**Fig. 2.—:**
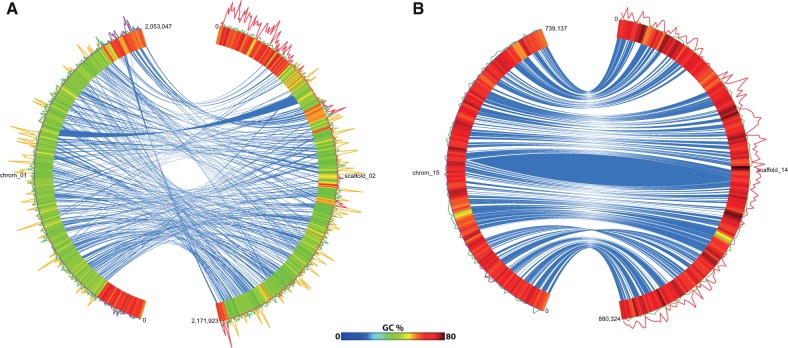
Genomic location of IEs. (*A*) Comparison of BOC chromosomes of RCC299 (left; chrom_01) and CCMP1545 (right: scaffold_02). (*B*) Comparison of chromosome 15 of RCC299 and scaffold_14 of CCMP1545. The outer band represents the GC percentage across the chromosome, while the inner connections (blue) represent orthologous genes between the two strains. Intron density is displayed on the outside of the outer band: IE-A/IE-B/IE-D (red), IE-C (purple), canonical introns (green), and BOC1 introns (yellow). Plots were drawn using Circos (Krzywinski et al. 2009).

IEs are not evenly distributed in the genome and are virtually absent from low-GC% areas, such as the AT-rich fraction of the BOC (fig. 2*A*). A second, so-called SOC, low in GC% and found in all mamiellophycean species reported so far, is also completely devoid of IEs. Other chromosomes tend to have the IEs distributed over their entire length, however with reduced densities in regions with lower GC% (fig. 2*B*). Their tendency toward high-GC% areas even surpasses canonical introns (fig. 1*B*).

We could not identify any sequence motif, both at the nucleotide level or the amino acid level that would correlate with the presence of IEs. There is also no insertion bias toward specific gene categories. On the other hand, the only functional category of genes completely lacking IEs involves genes that code for ribosomal structural components. However, it is well known that these genes are intron-poor and have a specific intron set—sometimes encoding small nucleolar RNAs—that helps to regulate the production and function of the ribosome (Parenteau et al. 2011), which could explain the absence of IEs due to strong selection against any further insertions.

The positioning of IEs within genes tends to favor the centre of the gene, which is similar to what has been recently reported for IE-like introns in fungi (van der Burgt et al. 2012). On the contrary, canonical introns in *Micromonas* are more often found at gene extremities (supplementary fig. S12, Supplementary Material online) and mostly in the genic 5′ region (Sakurai et al. 2002), a feature primarily ascribed to intron loss at the genic 3′ region (Nielsen et al. 2004).

### Replication

When searching marine metagenomes (at NCBI [http://www.ncbi.nlm.nih.gov, last accessed November 28, 2013] and CAMERA [Sun et al. 2011]) for IEs, we uncovered 2,794 metagenomic sequences containing complete or partial IEs. This finding confirms that the IEs are not an artefactual strain feature but are present in the ocean within a wider variety of strain-related organisms. When comparing both *Micromonas* genomes to these metagenomic samples (Sun et al. 2011), we discovered PAPs of IEs (supplementary fig. S13, Supplementary Material online). In total, 913 metagenomic sequences revealed 511 unique novel IE insertions. Most metagenomic sequences containing IE-A elements were highly identical to the CCMP1545 genome, while for IE-C-containing sequences, a higher degree of diversity was found (supplementary fig. S14, Supplementary Material online). At the same time, we discovered about 13 times more metagenomic sequences with novel IE-C positions compared with IE-A or IE-B/IE-D for which we have no proof for “novel” insertions (IE-A: 35; IE-B: 0; IE-C: 476; IE-D: 0).

The difference in PAPs can be explained by IE-C either being more active or IE-C being more widespread, or a combination of both. Besides in metagenomic sequences, an occurrence of IE-C-containing sequences was observed within the CCMP1764 strain (*Micromonas pusilla* clade-I) (Worden et al. 2009), for which short-read sequences have been obtained. After assembling the CCMP1764 genome, we compared it with the RCC299 genome. Only 31 IE-C positions are conserved in both genomes, while 149 and 66 are unique to RCC299 and CCMP1764, respectively, indicating that IE-C has been actively replicating since the divergence of RCC299 and CCMP1764.

Comparison with metagenomic data thus suggests that IEs are mobile elements that can replicate themselves and transpose into new locations. IEs are only found in transcribed regions in the sense orientation, which suggests that their mobility is linked to the transcription/splicing process. The mechanism most likely to explain this scenario is known as intron transposition (Lynch and Richardson 2002; Yenerall and Zhou 2012) (supplementary fig. S15, Supplementary Material online). Under this scenario, an IE can invade a transcript by reverse splicing. The resulting IE-containing transcript is subsequently reverse transcribed after which the cDNA undergoes homologous recombination with the corresponding genomic locus. The final result is that the IE is now found at a novel position in the genomic sequence.

An analysis of orthologous introns between CCMP1545 and RCC299 genes revealed 32 cases of IE remnants buried within conserved canonical introns. There are also several cases of nested or merged IEs, i.e., IEs inserted inside or merged with another IE (supplementary figs. S16 and S17, Supplementary Material online). Therefore, the “mobility phase” of IEs has to occur at a stage that still features a non-spliced primary transcript and not at the mature mRNA level.

## Discussion

The genomes of the tiny unicellular Mamiellophyceae are among the smallest found in eukaryotes (Derelle et al. 2006; Keeling 2007; Worden et al. 2009; Moreau et al. 2012). Genome analysis shows that they all lack the U12 minor spliceosome components (Bartschat and Samuelsson 2010). Consequently, it is surprising to find such a complex intron landscape within this taxon, with *Micromonas* CCMP1545 harboring five different classes of U2 spliceosomal introns, a unique feature never documented in any other eukaryote up to now. Analysis of intron size in eukaryotic genomes usually gives a typical distribution, as shown for plants (using *Arabidopsis thaliana* as a representative) and algae (using *Chlamydomonas reinhardtii* as a representative) with a single or major peak of small introns and a tail or a shoulder of big introns, which usually results from the insertion of transposable elements or other repeat elements (Iwata and Gotoh 2011) (fig. 1). Two of the five *Micromonas* intron classes, namely canonical introns and BOC1 introns, are observed in all species of Mamiellophyceae (Moreau et al. 2012). Canonical introns are found on most chromosomes, contain conserved splice signals, and their number is limited to a few per gene. On the contrary, BOC1 introns are restricted to a specific area of the genome, do not display any conserved signals, and their hosting genes can contain high numbers of them. Adding to this complexity, we described the presence of four independent populations of invasive introns of unknown origin, with numbers amounting to some 6,100 copies in the CCMP1545 strain, compared with a population of 4,300 resident introns. The unique dual genome architecture of Mamiellophyceae, unicellular picoeukaryotes with an abundant population size, coupled with the extra complexity derived from the intron invasion, strongly contradicts the idea that intron-rich architecture complexity arose in multicellular eukaryotes of small population size (Lynch and Conery 2003; Lynch 2006). It is unclear how the U2 spliceosome is able to deal with the different intron classes that presumably have different splicing efficiencies, and which evolutionary mechanisms have directed this intron diversity and invasion. Since their discovery (Gilbert 1978), the origin of spliceosomal introns in eukaryotes has been heavily debated, with tenants of the intron-early theory stating that the early eukaryotes already contained numerous introns, and proponents of the intron-late theory arguing for a gradual increase in intron numbers throughout evolution (Rogozin et al. 2012). Among the latest proposals on the origin of spliceosomal introns, it was suggested that they were acquired from mitochondria group II introns at the dawn of eukaryote evolution, right after the engulfment of the bacterial ancestor giving rise to mitochondria. They would then have invaded the ancestral eukaryotic genome with a concomitant need to create a nuclear compartment that allows the slow process of splicing to be completed before translation could be initiated (Martin and Koonin 2006). The presence of introns at homologous positions in orthologous genes in a large number of widely divergent eukaryotes rules in favor of the intron-early scenario, which consequently has led to the consensus that the Last Eukaryotic Common Ancestor contained intron-rich genes that more or less have been lost in different lineages (Lynch and Richardson 2002; Collins and Penny 2005; Roy and Gilbert 2005; Csuros et al. 2011).

However, recent studies seem to imply that intron gain is more widespread than previously thought (Roy and Penny 2007), leading to a more balanced view of intron origin (Koonin 2006). Recurrent intron gain in genes of prokaryotic origin has been observed after lateral gene transfer to eukaryotic taxa, an event that was suggested to be selected in intron-rich host genomes by nonsense-mediated decay (NMD) (Da Lage et al. 2013).

Peculiar intron gains were recently observed in the pelagic tunicate *Oikopleura dioica* (Denoeud et al. 2010), the microcrustacean *Daphnia pulex* (Li et al. 2009), the dothideomycete fungi *Mycosphaerella graminicola* (Torriani et al. 2011) and *Cladosporium fulvum* (van der Burgt et al. 2012), and of course *M. pusilla* CCMP1545 (Worden et al. 2009), a list to which we are now adding *Micromonas* sp. RCC299. Within the same species, newly gained introns were found to be highly similar in sequence, except for *Daphnia*. In *D. pulex*, 24 cases of intron gain were observed when comparing genomic sequences of two different genomes and sequences from natural isolates, but those gains were independent from each other, even gains occurring at the very same site. Regarding *O. dioica*, although its introns have several features in common with those of *Micromonas*—they are present mostly at unique positions and show noncanonical splice sites especially for newly gained introns—only four pairs of nearly identical introns (NIIs) were found out of a total of ∼75,000 introns. In this case, both NIIs in a pair were found within the same gene and were suggested to be the result of reverse splicing. In fungi, intron gain due to the insertion of near-identical introns (introner-like elements [ILEs], analogous to *Micromonas*’ IEs), shares some features with IE insertions. Depending on the species, ILEs occur in a range of a few tens up to ∼500, out of a total of more than 10,000 introns. Within the *Mycosphaerellaceae* species, they are related to each other, suggesting the presence of ILEs predating speciation within this clade ∼100 Ma. ILEs were shown to be efficiently spliced but to share specific features compared with resident introns, such as a bigger size and a conserved secondary structure. Finally, ILEs were shown to slowly degenerate with time, loosing progressively these specific features, and were thus suggested to be ancestors of many resident introns.

What makes *Micromonas* stand out is first and foremost the amplitude of intron gain, with hundreds to thousands of newly gained introns—comparable in number to an invasion of transposable elements. Because of its huge numbers, IE invasion can truly be seen as an intron-late case, in which the organisms’ intron content is significantly enriched, more than doubled in the case of CCMP1545. These IE numbers must impact the biology of *Micromonas*, while the other reported intron gains would likely not. The second difference lies in the genome characteristics. *Micromonas*, just like all other Mamiellophyceae, only contains a few resident introns, whereas the organisms listed above are intron-rich, although to a lower extent for *Mycosphaerellaceae* fungi, for which the number of introns lies between 1 and 2 introns per gene (Ohm et al. 2012). The argument of intron gain as a way to homogenize gene architecture through NMD (Lynch 2006; Lynch and Conery 2003) is falling short with the *Micromonas* IEs. Contrary to ILEs, we did not observe a clear or peculiar secondary structure within IEs. Finally, the intron invasion in the unicellular *Micromonas* goes against “simple population-genetic principles” stating that the selective disadvantage of intron-containing alleles, even if weak, would be a barrier to the proliferation of introns in organisms with a huge population size (Lynch 2002).

**Fig. 3.—:**
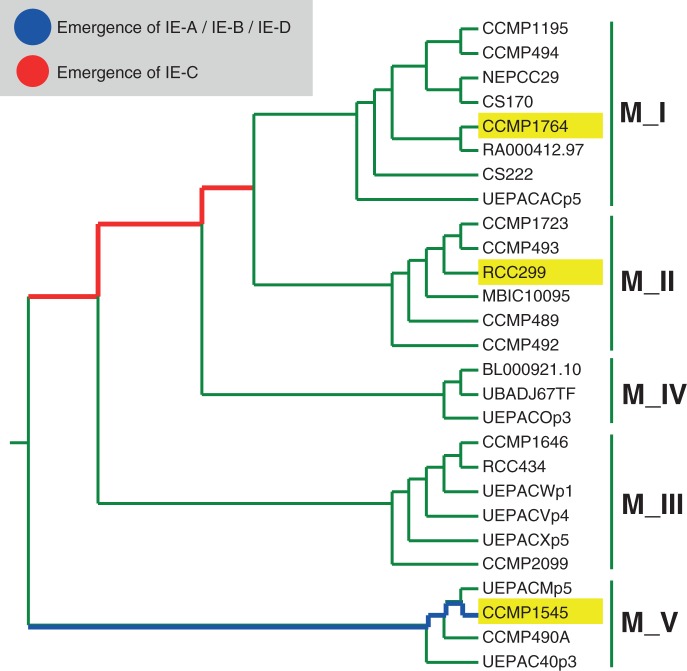
*Micromonas* phylogeny (adapted from Worden et al. 2009) inferred by neighbor joining based on 18S rRNA sequences. The tree shows the time windows in which the different IE classes have likely emerged with relation to the divergence of *Micromonas* isolates and strains and their clustering into five major clades, M_I to M_V. Isolates mentioned in the article are highlighted (yellow).

We propose that, at a given point during evolution, a genetic element such as the IE has arisen after which it started to replicate, as for ILEs. Because all intron gain events listed above vary greatly in sequence, these events must have happened independently from each other, in contrast to ILEs. In the case of both *Micromonas* isolates, metagenomic evidence suggests that IE-C is present in a wider variety of host *Micromonas* organisms, as metagenomic sequences containing IE-C display a higher degree of sequence variety than IE-A/IE-B ones (supplementary fig. S14, Supplementary Material online). This explains why *M. pusilla* CCMP1764, which belongs to a different clade than RCC299 (Worden et al. 2009), also carries IE-C sequences. IE-C therefore needs to have originated in an ancestor of clade I and II, but after the divergence of clades III and V. As of now, IE-A/IE-B seems to be restricted to clade V (fig. 3).

As reported for fungi (van der Burgt et al. 2012), IEs degrade over time and undergo mutations and indels (with a bias toward deletions) until the IE signature “fades out.” It is therefore possible that many of the *Micromonas* introns that we now label as canonical are in fact highly degraded IEs.

Various mechanisms have been suggested to explain intron gains, such as gene duplication, insertion of transposable elements, mutational creation of novel splice sites, or splicing enhancing features. Our findings as well as other recent ones implying propagation of intron copies do favor the reverse-splicing/recombination scenario (Roy and Irimia 2009) suggested earlier by Cavalier-Smith (1985). In the first step of this scenario, an intron freed from one pre-mRNA would be inserted into another pre-mRNA by the splicing machinery (supplementary fig. S15, Supplementary Material online). Reverse splicing, which was initially a rather wild hypothesis, nowadays turns out to fit with the current knowledge as it has recently been established in yeast that the two splicing steps are indeed reversible (Tseng and Cheng 2008). The second and third step should be the retro-transcription of the pre-mRNA into cDNA and the subsequent homologous recombination of this cDNA with its genomic partner (supplementary fig. S15, Supplementary Material online), both steps being documented in model eukaryotes and supported by the occurrence of intron loss for which they are required as well.

Why are IEs and other copy-introns specifically invasive and which features make these introns so successful in their capability to invade genomes while resident introns are generally noninvasive? Analysis of the transcriptome shows that transcripts for IE-containing genes are often not properly spliced, with many copies showing intron retention of IEs. This observation, together with the unusually high occurrence of noncanonical splice sites, argues for the *Micromonas* spliceosome to be permissive but rather ineffective for the newcomer introns that have not yet evolved the most efficient splicing mechanism, a hypothesis previously been put forward to explain evolution of mechanisms of RNA surveillance (Lynch and Kewalramani 2003). As a consequence, one would expect that IE splicing inefficiency would end up in promoting a proofreading mechanism, shunting the refractory spliceosome-bound pre-mRNA to a discard pathway (Hoskins and Moore 2012). This alone may in turn increase the chance for IE reverse splicing, which has been experimentally shown to happen under circumstances that favor spliceosome trans-conformation (Tseng and Cheng 2008).

Some questions remain. What is the pace at which mobile introns are created and how long do they remain invasive? Are the mechanisms that control intron abundance similar to those observed for transposable elements? Finally, are *Micromonas* IEs and other cases of mobile introns just isolated exceptions to the rule, or are we on the verge of discovering many more hidden cases which would impact our view on the evolution of eukaryotic genome architecture, where intron invasion in eukaryotes would have occurred continuously?

## Conclusions

The *Micromonas* strains CCMP1545 and RCC299 display a complex intron landscape, carrying canonical spliceosomal introns, Mamiellophyceae-specific introns (BOC1), and different classes of IEs. These IEs have colonized the genome by copying themselves into genes, likely involving reverse splicing. The findings presented in this article further strengthen the idea that intron gain is more widespread than previously thought.

## Supplementary Material

Supplementary figures S1–S17 and supplementary methods are available at *Genome Biology and Evolution* online (http://www.gbe.oxfordjournals.org/).

Supplementary Data
