# Supplementary material for: The Complex Intron Landscape and Massive Intron Invasion in a Picoeukaryote Provides Insights into Intron Evolution
Source: Genome Biol Evol. 2013 Nov 20;5(12):2393–401. doi: 10.1093/gbe/evt189 (PMC3879977; doi:10.1093/gbe/evt189)
Supplement: Supplementary Data [file supp_5_12_2393__index.html]

The complex intron landscape and massive intron invasion in a picoeukaryote provides insights into intron evolution — The Complex Intron Landscape and Massive Intron Invasion in a Picoeukaryote Provides Insights into Intron Evolution — Supplementary Data 

# The Complex Intron Landscape and Massive Intron Invasion in a Picoeukaryote Provides Insights into Intron Evolution

## Supplementary Data

files

**Files in this Data Supplement:**

- Supplementary Data - pdf file
