# Supplementary material for: The Complex Intron Landscape and Massive Intron Invasion in a Picoeukaryote Provides Insights into Intron Evolution
Source: Genome Biol Evol. 2013 Nov 20;5(12):2393–401. doi: 10.1093/gbe/evt189 (PMC3879977; doi:10.1093/gbe/evt189)
Supplement: Supplementary Data [file supp_evt189_introner_SUPP_v7.1_genomebiologyevolution.pdf]

The complex intron landscape and massive intron invasion in a picoeukaryote provides insights into intron evolution

Bram Verhelst[1,2], Yves Van de Peer[1,2,3,*], Pierre Rouzé[1,2,*]

[1] Department of Plant Biotechnology and Bioinformatics, Ghent University, Technologiepark 927, B-9052 Ghent, Belgium

[2] Department of Plant Systems Biology, VIB, Technologiepark 927, B-9052 Ghent, Belgium

[3] Department of Genetics, Genomics Research Institute, University of Pretoria, Pretoria, South Africa

[*] to whom correspondence should be addressed

Supplementary Information

Supplementary Methods

Introner Element (Remnant) Prediction

Starting from handpicked example Introner Elements (IEs), we delineated common motifs (pattern blocks) and assembled them into class-specific pattern files (IEA-1 example listed below). We used PatScan (Dsouza, et al. 1997) to scan the *Micromonas* genomes. For each class, multiple pattern files were constructed, ranging from strict to degenerate. When overlapping matches were detected, only the match belonging to the strictest pattern file was kept. EST and protein alignments were generated using GenomeThreader (Gremme, et al. 2005) (v1.4.6; -minalignmentscore 0.95 –mincoverage 0.89) and the splicing information was used in the automated curation of the final set of predicted IEs i.e. adjusting IE start and stop coordinates to match the exon/intron boundaries.

```
p1=GTGCGT
0...15
p2=ACTGGTYCCCRTACGACC[5,0,0]
0...80
p3=STTTCAAT[2,0,0]
0...40
p4=GCCTTTCAACTC[3,0,0]
0...100
p5=AG
```

IE remnants were detected using BLASTN (v2.2.17; -e 1e-05) (Altschul, et al. 1990) using the previously built set of (complete) IEs. For each class, we also built a multiple sequence alignment (MSA), constructed a profile HMM (HMMer v2.3.2), and used it to detect additional instances of degenerated IEs. This HMM approach was also used for members of the IE-B / IE-D class.

Micromonas Re-annotation

IEs were added as an extra evidence track when performing the annotation (EuGene v3.6 (Schiex, et al. 2001)). EST and protein libraries of all Mamiellophyceae were used, as well as extensive sets of manually curated gene models. RNA genes were predicted using tRNAscan-SE (Lowe and Eddy 1997) and Infernal (Griffiths-Jones, et al. 2003). Genes encoding selenoproteins were manually corrected. When compared to the old annotation, the new annotation features fewer but larger gene models. This is mainly due to the IEs that help to span stop codons, allowing adjacent gene models to be merged into one continuous model.

Gene Ontology analysis of IE genes

GO terms for all *Micromonas* proteins were derived using InterPro2GO (Mulder and Apweiler 2007), and GO term over/under-representation of genes carrying IEs, using the GO terms of the entire *Micromonas* proteome as a background, was analysed using the Cytoscape plugin BiNGO (Maere, et al. 2005) (hypergeometric test + FDR correction; significance level 0.05). This GO analysis was only performed on CCMP1545, as the low number of IEs in RCC299 makes the analysis insignificant.

Spliceosomal Components

Spliceosomal components were detected through homology with *A. thaliana* proteins in the Splicing Related Gene Database (http://www.plantgdb.org/SRGD) and through the detection of splicing-related GO labels.

Metagenomic Sequence Analysis

When aligning metagenomic sequences (MSs) to the *Micromonas* genomes, the presence or absence of IEs in both the query (the MS) and the genomic sequence can present too big a gap in the alignment for 'regular' alignment programs to cope with. As such, the environmental sequences were aligned to the genomes using a seed-and-align procedure, initiated by a regular BLASTN. Starting from the best-hit, we expanded the genomic space with neighbouring hits. In the end, we used the outer coordinates to extract the corresponding genomic region, and re-aligned it to the environmental sequence using a SMITH-WATERMAN alignment (EMBOSS (Rice, et al. 2000): water).

To be able to draw accurate conclusions on IE presence/absence polymorphisms (PAPs), we performed a quality filtering step. We only continued with alignments that have more than 100 nucleotides labelled as 'non-IE', an identity percentage of more than 50%, and a coverage of more than 60%. After careful consideration, we also decided to leave aside all metagenomic sequences labelled as 'JCVI', as they were assemblies of smaller metagenomic sequences.

RNA secondary structure analysis

For each IE class, a consensus sequence was constructed (EMBOSS (Rice, et al. 2000): cons). Secondary structures were predicted using RNAfold (Vienna RNA (Hofacker 2003)). Secondary structures were compared between classes, but no general model could be obtained.

Protein evidence for IE-D

The protein sequences of genes containing IE-D sequences and their selected orthologs were aligned using Clustal W (default settings: GONNET weight matrix) (Thompson, et al. 1994).

Visualisation tools

For visualisation purposes, we also employed the following tools: seqlogo (Crooks, et al. 2004) and R (v2.13.0) (R Development Core Team 2008).

Supplementary References

Altschul SF, Gish W, Miller W, Myers EW, Lipman DJ 1990. Basic local alignment search tool. J Mol Biol 215: 403-410. doi: 10.1006/jmbi.1990.9999

Crooks GE, Hon G, Chandonia JM, Brenner SE 2004. WebLogo: a sequence logo generator. Genome Res 14: 1188-1190. doi: 10.1101/gr.849004

Dsouza M, Larsen N, Overbeek R 1997. Searching for patterns in genomic data. Trends in genetics : TIG 13: 497-498.

Gremme G, Brendel V, Sparks ME, Kurtz S 2005. Engineering a software tool for gene structure prediction in higher organisms. Information and Software Technology 47: 965-978. doi: 10.1016/j.infsof.2005.09.005

Griffiths-Jones S, Bateman A, Marshall M, Khanna A, Eddy SR 2003. Rfam: an RNA family database. Nucleic Acids Res 31: 439-441.

Hofacker IL 2003. Vienna RNA secondary structure server. Nucleic Acids Res 31: 3429-3431.

Lowe TM, Eddy SR 1997. tRNAscan-SE: a program for improved detection of transfer RNA genes in genomic sequence. Nucleic Acids Res 25: 955-964.

Maere S, Heymans K, Kuiper M 2005. BiNGO: a Cytoscape plugin to assess overrepresentation of gene ontology categories in biological networks. Bioinformatics 21: 3448-3449. doi: 10.1093/bioinformatics/bti551

Mulder N, Apweiler R 2007. InterPro and InterProScan: tools for protein sequence classification and comparison. Methods in molecular biology 396: 59-70.

R Development Core Team. 2008. R: A Language and Environment for Statistical Computing. In.

Rice P, Longden I, Bleasby A 2000. EMBOSS: the European Molecular Biology Open Software Suite. Trends in genetics : TIG 16: 276-277.

Schiex T, Moisan A, Rouzé P. 2001. EuGène: An Eukaryotic Gene Finder That Combines Several Sources of Evidence. Selected papers from the First International Conference on Computational Biology, Biology, Informatics, and Mathematics: Springer-Verlag.

Thompson JD, Higgins DG, Gibson TJ 1994. CLUSTAL W: improving the sensitivity of progressive multiple sequence alignment through sequence weighting, position-specific gap penalties and weight matrix choice. Nucleic Acids Res 22: 4673-4680. doi: 10.1093/nar/22.22.4673

Supplementary Figures

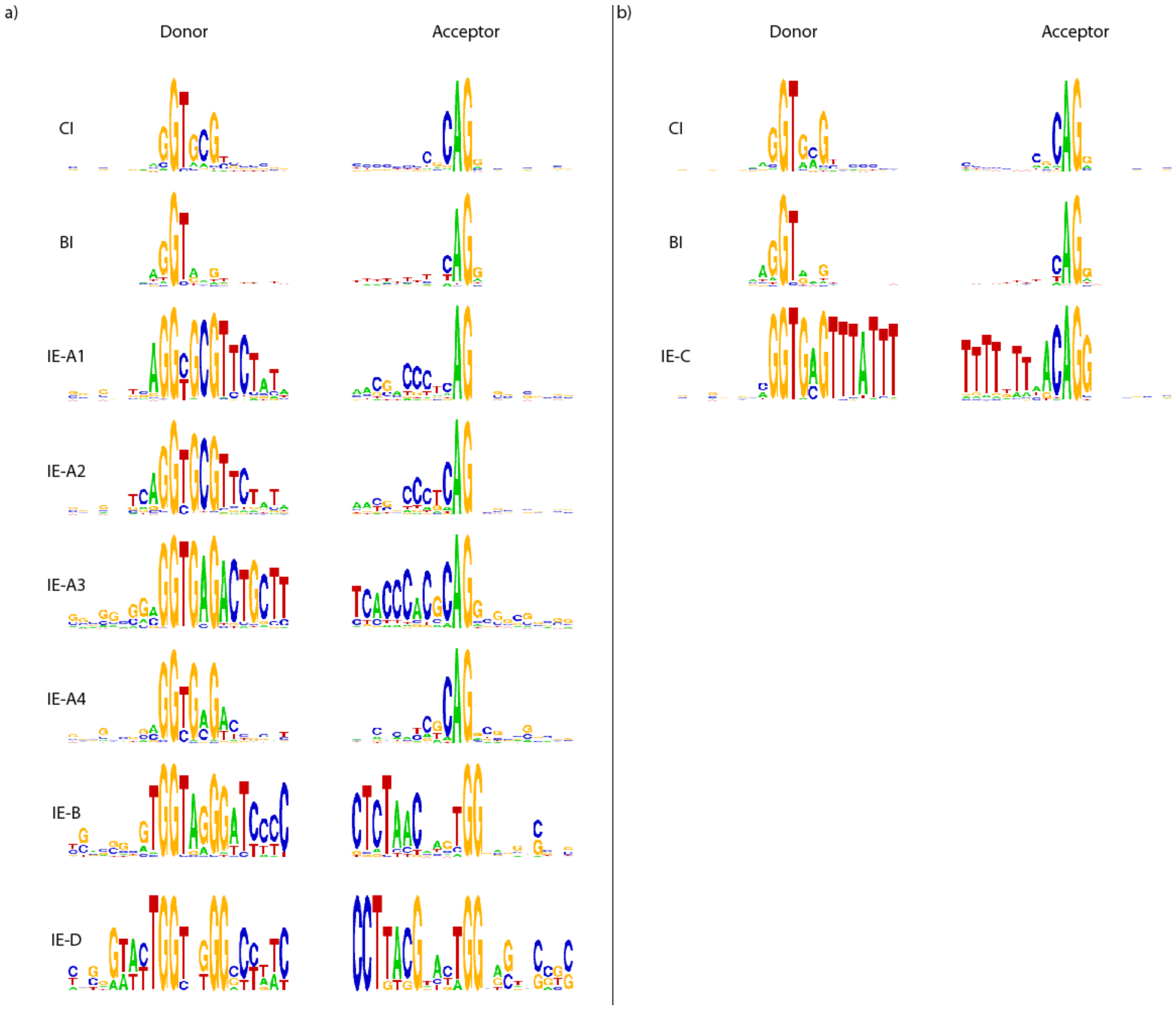

Figure S1. *Micromonas* splice site signals for all intron classes. Shown here are sequence logos for the donor/aceptor site (10 nucleotides upstream and downwstream) for CCMP1545 (**a**) and RCC299 (**b**).

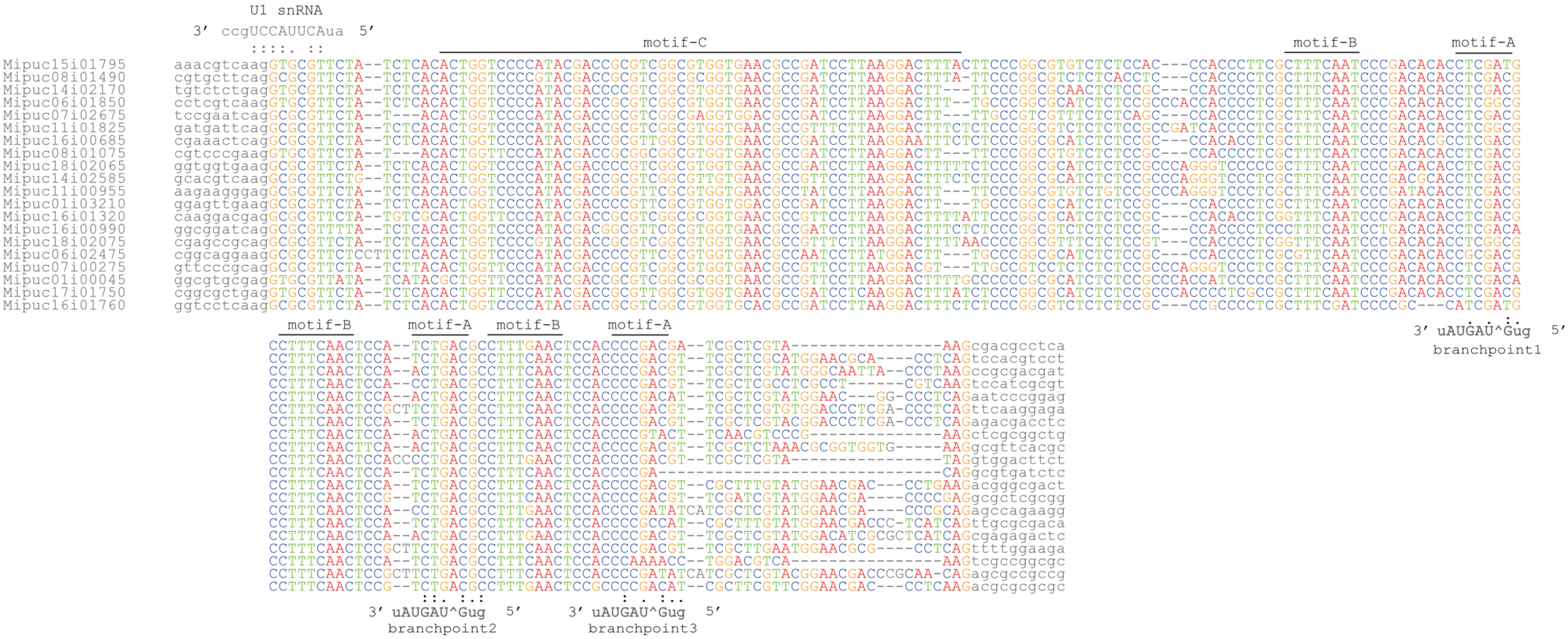

Figure S2. Alignment of 20 random IE-A1 sequences. The motifs (motif-A = branchpoint motif; motif-B: branch-point companion motif; motif-C) are marked, as are the splicing signals (donor site + branch-point) and their base-pairing information from the corresponding spliceosomal RNAs.

```
                            U1 snRNA
                         3’ ccgUCCAUUCAua  5’
                               ::::. ::                                                   motif-B motif-A                           motif-B  motif-A
Mipuc04i02095      tttgcatcagGTGCGTTCTATACAAACGTGTTTCACC----CATCGCTCGGTTTCAACACTTGATCGCGT---CC----CCTTTCAACTGACCG----GTGAACTTTTTTTGTATGGAATGGCCCT---CAGgtgacggcgg
Mipuc06i01490      ggtcgttacgGTGCGTTCTATACAAACGTGTTTCACC----CACCGCTCGGTTTCAACACTTGATCGTGT---TC----CCTTTCAACTGACCG----GTGAACTTTTTTTGTATGGAATGGCCCT---AAGattcacgaag
Mipuc04i00480      tggagatcagGCGCGTTCTAT------GTTTTTCACC----CATCGCTCGGTTTCAACACTTGATCGCAT---AC----CCTTTCAACTGACCG----GTGAACTGTTTTTGTATGGAATGGCCCTCA-CAGagcaatcgta
Mipuc16i00225      atacctccagGTGCGTTCTATACAAACGTTTTTCACC----CATCGCTCGGTTTCAACATTTGATCGCGT---CC----CCTTTCAACTGACCGATGAATGAACATCTTTTCTATGGAATGGCACTCACCAGctgctgtacg
Mipuc11i00510      tctttctcagGCGCGTTCTATACAAAAGTTTTTCACC----CATCGCCCGGTTTCAACATTTGATCGCGT---CT----CCTTTCAACTGACCG----GTGAACTATTTTTGTATGGAACGACCCT---CAGacgatgcgtc
Mipuc15i02010      gcggagtaagGTGCGTTCTATACAAAAGTGTTTCACC----CATCGCCCGGTTTCAACACTTGACCGCGT---CT----CCTTTCAACTGACCG----GTGAACTATTTTTGTATGGAACGACCCT---AAGgaggcgatgg
Mipuc02i00255      ccacgctcagGCGCGTTCTATACAAAAGTGTTTCACC----CATCGCCCGGTTTCAACACTTGATCGCCT---CC----CCTTTCAACTGACCG----ATGAACTATTTTTGTATGGAACGACCCT---CAGctcgtggacg
Mipuc07i00805      ggcgcttcagGCGCGTTCTATACAAAAGTTTTTCACC----CATCTCCCGGTTTCAACATTTGATCGCTC---CC----CCTTTCAACTGACCG----GTGAACTTTTTTTGTATGGAACGACCCT---CAGttccagtcgg
Mipuc06i01455      gcgcgttcagGCGCGTTCTATACAAAAGTTCTTCACC----CATCGCTCGATTTCAACACTTGATCGCTC---CC----CCTTTCAACTGACCG----GTGAACTTTTTTTGTATGGAACGACCCT---CAGaggcagcggc
Mipuc06i02190      gcggtttcagGTGCGTTCTATACAAACGTTTTTCACC----CATAGCTCGGTTTCAACACTTGATCGCGTGGGTC----CCTTTCAACTGACCG----ATGAACTATTTTTGTATGGAACGACCCT---CAGtcttccgcgt
Mipuc07i01580      gctgtaccagGCGCGTTCTATACAAAAG-TTTTCACC----CATAGCTCGGTTTCAACACTTGATCGCGT---GCGTCTCCTTTCAACTGACCG----ATGAACTATTTTTGTATGGAACGACCCT---CAGgcgttgatcg
Mipuc01i01490      ctcgagtcagGCGCGTTCTATACAAAAGTTTTTCACC----CACCGTCCGGTTTCAACATTTGATCGCGT---CC----CCTTTCAACTGACCG----ATGAACTATTTTTGTATGGAATGGCCCT---CAGtgcgaccggc
Mipuc06i02400      gccgggttagGTGCGTTCTATACAAAAGTTTTTCACC----CATCGCCCGGTTTCAACATTTGATCGCGTGGGTC----CCTTTCAACTGACCG----GAGAACTGTTTTTGTATGGAACGGCCCT---CAGtcgggtcgat
Mipuc15i01490      tattcgtcagGTGCGTTCTGTACAAAAGTTTTTCACC----CGTCGCCCGGTTTCAACATTTAATCGCGT---CC----CCTTTCAACTGACCG----GAGAACTGTTTTTGTATGGAACGGCCCT---CAGggaccgcgcg
Mipuc10i01100      gggtgtcagGTGCGTTCTCTACAAAAGTTTTTCACT----CATCGCCCGGTTTCAACATTTGATCGCTC---TC----CCTTTCAACTGACCG----GTGAACTGTTTTTGTATGGAATGGCCCT---CAGggtgacgtac
Mipuc10i02050      tcgttatcagGTGCGTTCTATACAAAAGTTTTTCTCCCGAGCACCGCTCGGTTTCAACATTTGATCGCTC---CC----CCTTTCAACTGACCG----GTGAACTGTTTTTGTATTGAATGGCCCT---CAGccaagctgag
Mipuc05i00370      acgtcgtaagGTGCGTTCTATACAAAAGTTTTTCACC----CATCGCCCGGTTTCAACAGTTGATCGCGT---CT----CCTTTCAACTGACCG----ATGAACTGTTTTTGTATGGAATGGCCCT---AAGgctctcgttc
Mipuc18i02040      ttcgacgaagGCGCGTTCTGTACAAAAGTTTTTCACC----CACCGCTCGGTTTCAACGTTTGATCGCGT---CC----CTTTTCAACTGACCG----ATGAACTGTTTTTGTATGGAACGCCCCG---AAGgctacaccgt
Mipuc04i01315      ggggcatcagGCGCGTTCTATGCAAACGTGTTTCACC----CATCGCTCGGTTTCAACACGTGATCGCGT---CC----CCTTTCAACTGACCG----GTGAACATTTTCTGTATGATT----------CAGtgtccgactg
Mipuc13i01340      ggagattcagGCGCGTTCTATACAAACGTTTTTCACC----CACCGCTCGATTTCAACATTTGATCGCGT---CC----CCTTTCAACTGACCG----ATGAACCTTTTTTT--GGGAACGCCTCT---CAGaggacgtcgc
                                                                          .:. .                            :::. :
                                                                      3’ uAUGAU^Gug  5’               3’ uAUGAU^Gug  5’
                                                                         branchpoint1                    branchpoint2
```

Figure S3. Alignment of 20 random IE-A2 sequences. The motifs (motif-A = branchpoint motif; motif-B: branch-point companion motif; motif-C) are marked, as are the splicing signals (donor site + branch-point) and their base-pairing information from the corresponding spliceosomal RNAs**.**

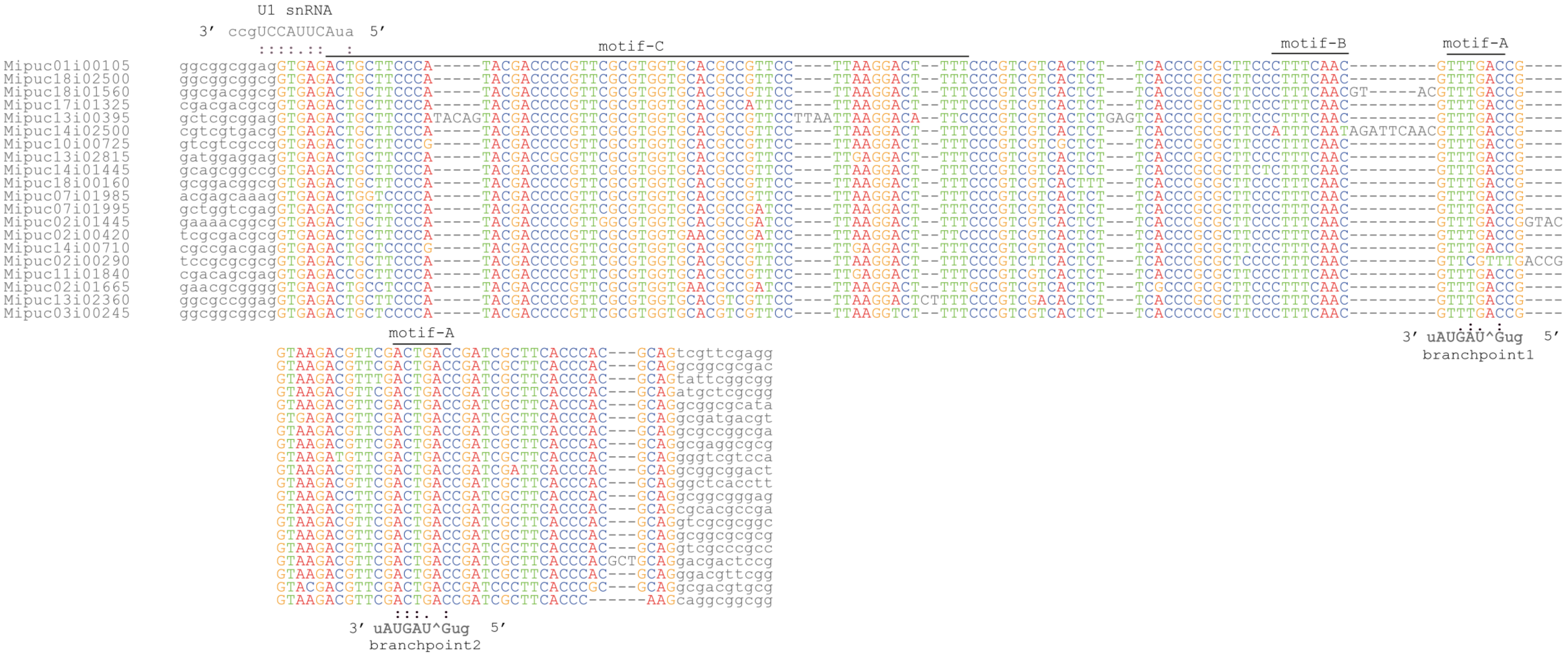

Figure S4. Alignment of 20 random IE-A3 sequences. The motifs (motif-A = branchpoint motif; motif-B: branch-point companion motif; motif-C) are marked, as are the splicing signals (donor site + branch-point) and their base-pairing information from the corresponding spliceosomal RNAs..

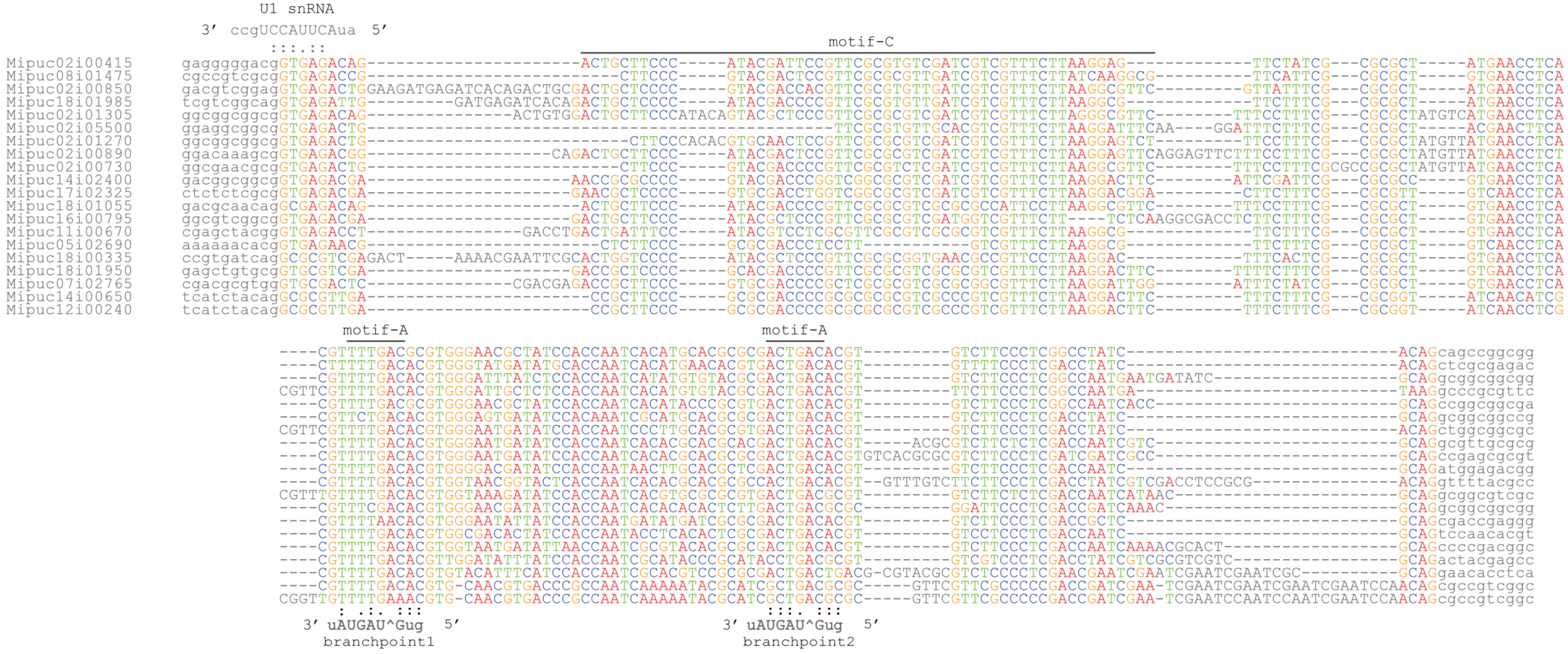

Figure S5. Alignment of 20 random IE-A4 sequences. The motifs (motif-A = branchpoint motif; motif-B: branch-point companion motif; motif-C) are marked, as are the splicing signals (donor site + branch-point) and their base-pairing information from the corresponding spliceosomal RNAs.

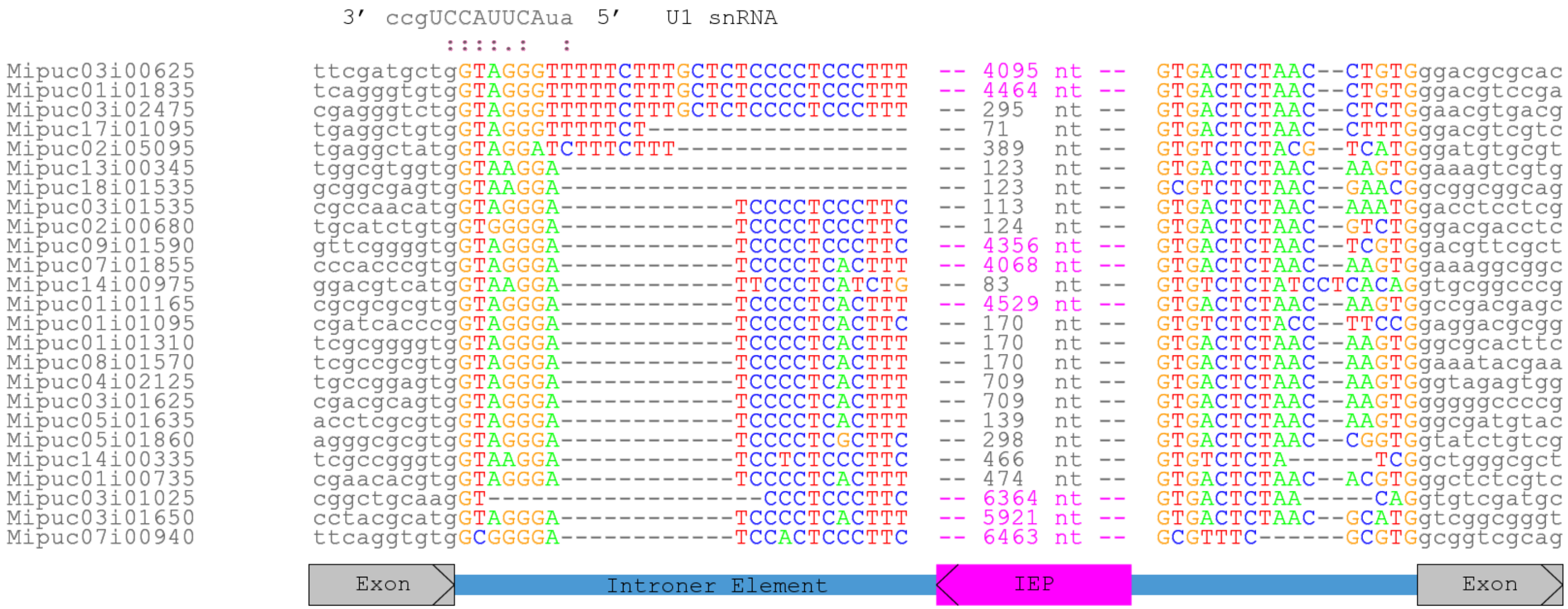

Figure S6. Alignment of all 25 IE-B sequences showing the splice site regions in detail. The structure and orientation of exonic regions (grey) and Intron-Encoded Proteins (purple) is represented schematically beneath the alignment. Base-pairing information regarding the donor site (U1 snRNA) is also provided.

```
Mipuc02i00175     tcgtgttttgGCA-GGGCC-----TGTCTGATTCTCCCGCCCTGTTTCTC------------------------------------------------------------
Mipuc02i02895     aagaattttgGTATGGATCTTCTCTTGAATCTGTCCCTACCTGTATCTCCCCTTCTTCCCTCTGCTTGTGTCATCGTTCGTCTCGACGACGACAATGACGCGACATTTTC
Mipuc02i02703     tcgggtactgGT-GGGGCC-----TATCTGGTTCTCCCGCCTGTTTCCCCTCTTTCATCCCCTGTGTATTCGTTCGTTCCATTACAATACAGTACCAGTCAGTCTGTTCC
Mipuc05i01652     ctcggaactgGTTGGGGCC-----TATCTAGTTCTCCCGCCTGTTTCCCCTCTTTCATCCCCTGTGTATTCGTTCGTTCCATTACAATACAGTACCAGTCAGTCTGTTCC
Mipuc16i02340     cgccgtattgGT-GGGGCC-----TATCTAGTTCTCCCGCCTGTTTCCCCTCTTTCATCCCCTGTGTATTCGTTCGTTCCATTACAATACAGTAA---------------
Mipuc16i00767     cgtggaactgGTCTGGGCCTACTGTATCTAGTTCTCCCGCCTGTTTCCCCTCTTTCATCCCCTGTGAACG----------------------------------------

                            ----------------------------------------------------------------------------------------------------
                            AT--------------------------------------------------------------------------------------------------
                            GTCGGACGATATCGCGACGCTGTCGCGGTGTGTCGTGTGTTGTTCTTCTACAGCTCTCTTTTCTCTCTCAATCTGGCGCCTGCTCGGCCTTCGCGTGCGC
                            GTCGGACGATATCGCGACGTTGTCGCGGTGTGTCGTGCGTTGTTCTTCTACAGCTCTCTTTTCTCTCTCAATCTGGCGCCTGCTCGGCCTTCGCTGCGAT
                            ----------------------------------------------------------------------------------------------------
                            ----------------------------------------------------------------------------------------------------

                            ----------------------------------------------------------------------------------------------------
                            ----------------------------------------------------------------------------------------------------
                            CTTCGCCTTCGCGTTCGAC---------------------------GATCGACGATCGGCGCGGCCTTGCACCCAATCCACCGGGGGGCTCTTCATCGCG
                            CAAGCACACCAGCTCGGACACGAGCTCATCGATCGCGTGCGCGTCGGCACGATCGTCGGCGCGGCCCTGCACCCAATCCACCGGGGGGCTCTTCATCGCG
                            ----------------------------------------------------------------------------------------------------
                            ----------------------------------------------------------------------------------------------------

                            ----------------------------------------------------------------------------------------------------
                            ----------------------------------------------------------------------------------------------------
                            ATGAGTTTGGGAACGCTCGGGCTCATGTCGAGGTCGTGCAGCTTGAAACCCTTGGGCATGCCTGG-----------------------------------
                            ATGAGTTTGGGAACGCTCGGGCTCATGTCGAGGTCGTGCAGCTTAAAACCCTTGGTCATGCCTGGAGCCACGCGGTTATGAGCCTTGGCTTCCACGCGCT
                            ----------------------------------------------------------------------------------------------------
                            ----------------------------------------------------------------------------------------------------

                            ----------------------------------------------------------------------------------------------------
                            ----------------------------------------------------------------------GTCCGTTTCTCCGTCGGTTTCTTCAAATGT
                            ----------------------------------------------------------------------AGCCACTCTTCCGTCGGTTTCTTCACATAT
                            -508 nt-TCTGACGCTCCCCTCTTTTTCCCTTGGGAAATTTTCAATCGATCGTCGCCGCCTTTCCCCTGTCCGTCTGTTCCGTCGGTTTCTTCACATAT
                            ----------------------------------------------------------------------------------------------------
                            ----------------------------------------------------------------------------------------------------

                            ----------------------------------GTCATCCATGCCACAAACG-TATATTTGTCGATGACTCACCTGGCTTTGATGAACGCACGCGTGAC
                            TGAATGACACA--------------GTCGTTCTCACTATGCATGACACACACATTCTATCTATCCATGACTCACCTGACTTTGATAAATGCACGC-----
                            CGAATGACACAGACGCTCTGACACGGACGTTCTCGTCATCCATGACACAAACG-TATATTTGTCGATGACTCACCTGGCTTTGATGAATGCACGT-----
                            CGAATGACACAGACGCTCTGACACGGACGTTCTCGTCATCCATGACACAAACG-TATATTTGTCGATGACTCACCTGGCTTTGATGAATGCACGT-----
                            ------------------------------------------------------------TGTCGATGACTCACCTGGCTTGGATGAATGCACGT-----
                            ------------------------------------------------------TGTATTTGTCGATGACTCACCTGGCTTTGATGAATGCACGT-----

                            TGTTCCCATACGACCGCGTTCGCGTCGCGAACTTCATCCCTTGAGGACTTTTTTCTCTCCCGATGCGACCTTACGGCGAGggaccgccgc
                            --------------------------------------------------------------ACGCGACCTGTGGCTTTGgcgcatcgtc
                            --------------------------------------------------------------ATGCGACCTTACGTACTGgtggtacatg
                            --------------------------------------------------------------ATGCGACCTTACGAACTGgcagtcgcgc
                            --------------------------------------------------------------ATGCGACCTTACGTATTGgatggcgccg
                            --------------------------------------------------------------ATGCGACCTTACGTACTGgaagtacggc
```

Figure S7. Alignment of all six IE-D sequences showing the splice site regions in detail. The IE-A remnant inside the chimeric Mipuc02i00175 has been highlighted in blue.

Figure S8. Protein evidence supporting IE-D introner elements (in lack of any EST evidence). Canonical introns (light blue), IE-A (green), IE-D (dark blue), and chimeric IEs (red) have been highlighted accordingly. Intron phases are indicated on top of the intron positions. Gene IDs correspond to the following organisms: *Ostreococcus lucimarinus* (Ol*), *Ostreococcus sp.* RCC809 (Od*), *Ostreococcus tauri* (Ot*), *M. pusilla* CCMP1545 (Mipuc*), *M.sp.* RCC299 (Mipur*), *Bathycoccus prasinos* (Bathy*), *Physcomitrella patens* (*PHYPHA), *Arabidopsis thaliana* (*ARATH) and *Dictyostelium discoideum* (*DICDI). a) Alignment of WD89 proteins. b) Alignment of Jumonji-1 proteins (Jmj-N and Jmj-C Jumongi domains are highlighted in grey, while the Zn-finger domain has been highlighted in yellow), c) Alignment of sepA-like Serine/Threonine protein kinase, d) Alignment of U5 snRNP helicase, e) Alignment of UAA1 proteins (UDP-N-acetylglucosamine; UMP antiporters).

a) WD89

```
                         10        20        30        40        50        60        70        80        90       100
                          |         |         |         |         |         |         |         |         |         |
Ol03g00040       M--TRTRDDDDAHDDDDRRGARALVDDSGAPNVFENDDYVEPTPESDEALTFTVRGG-----------RASTTAMSLRATSMTSTAVDKDAPYNTHIASN
Od03g03130       M----SRDD--------------LVDDSGAPNAFENDGYDEPGPESDEALTHALPAAGT--------TASDVDAVALVARSHASTSVKGDAPYNVHVATN
Ot03g00170       M-RDATVEDD-------------FVDRSGAPSAFENDGYDEPDATNDEALTFQCARG------------GGDGSAALVARATRTTAREGDAPYNTHLSVS
Mipuc02g00100    M------PS------------------RLAPACTTVAAFG---PSHDPCYVMALAPRSVRARLVRVPPHVPPTLRSARVDRSLAPTRLLPPPPSPLPRRS
Mipur03g06080    MYRPASNPSP--------------CH-HLAPVSTTVAAFG---PQNDPCYVMAIASR------------YV--AR---AHPTPSPAAPLSLPPS------
                         .                    **       :    .  * . .                           .    :.:     * .

                        110       120       130       140       150       160       170       180       190       200
                          |         |         |         |00       |         |         |         |         |         |
Ol03g00040       HDGTSCAVATTSGRVRVYARERATGAFAHG---------------EREIRLNASVNECEFGSAADPFSIVCACGDGTTRVYDARSQ-DDRPTASFRAPFN
Od03g03130       HDGGVVAVVTTSGRAVVYERRGTDGGLETET--------------ARDVEVGCVVNECEFGPREDPYSLVCACGDGTVRVYDARCG-DSRAVASFRAPAG
Ot03g00170       ADGAMCAVTTTSGRVRIIARDGGSGALGDDAR-------------EFVPGHGCAVNECEFGSASDPHAIVLACGDGTVRVYDVRTK-DDRATAVFRAPYG
Mipuc02g00100    PDGAHLAASLSTNAVKIYARGDAPSGGDFGGGGGGGGHLGTLRAVTELSAHDGAVTDCAFPIPSEPWTVLTSSADATIRAWDLRQG-GERPCASYVAPFA
Mipur03g06080    SDGKYLATSLSTQAVKVYQR--------TED--G------GIAALVELTGHGAAVTDVTIPLPHEPCTVCSSSLDGTVRLWDCRAAEGQREVMKFVAGFA
                  **   *.  ::  . :  *                               .  *.:  :    :* ::  :. *.* * :* *   ..*    : *
```

```
                       210       220       230       240       250       260       270       280       290  s    300
                         |    1    |         | 2       |         |         |         |         |         |  1      |
Ol03g00040      ER---DVASASLGGSASDFLVATAVGPHVAFYDRRQQG----NASLFQDAHSEAVTRVRFHPERRSELYTSSVDGLVCAFDCA--HTPLNDEESLISVMS
Od03g03130      ER---DVCSATLGGGATGSMIAAAVGPHVVFFDRRRHGADASHADMFRDAHSEAVTRARFHPERRRELYTSSVDGLMCAFDCS--RAPLNDEDALIAIIS
Ot03g00170      ER---DVPSASLGGDG-DTLVAAAAGAHVVFYDRRTQGGD-ACVGLFQDAHSEVVTRVRFHPERRSELYTSSVDSLVCAFDCS--RAPLNDEDALITIMS
Mipuc02g00100   TRATGGFATATAGGVG-DHLVVAGWTNQIVFWDRRTREG----LRAFEDAHSEDVTRVRFQPTRRNRLFTASVDGLACVFDVGGCPADVDDEDGLLCVMT
Mipur03g06080   K----EFASATLGGGN-DHLVVGAQNEQVVFWDRRVGTG----LEVFEDSHSEDVTRVRFQPGRRNRLFTAGVDGLACAFDVGGCPADINDEDGLLTVMH
                      . :*: **   . ::. .   ::.*:***           *.*:*** ***.**:* ** .*:*:.**.* *.** .   : ::**:.*: ::

                       310       320       330       340       350       360       370       380       390       400
                         |    00   |         |         |         |         |        1|         |         |         |
Ol03g00040      AEAAVNNIGFCRS---GSSGDARDALWCVTGVEDAHIFVASS-DRRRVGVHLVHLKNARELVRTAATS-SPEVSASAPEFAAQVDYVLGIHDGVA-----
Od03g03130      ADAAVNTIGFCRS---GASGDERDAVWCATGVEEAHVFVASS-DRRRVGVHLAHLKNAREVARAATTN-SPRASSTAPEFLAGVDYIVGVHDGVA-----
Ot03g00170      ADAAVNNIGFCRT---GASGNERDAVWCTTGIEEAHIFLTTSSDKRRVGVQLVHLKNARALAQTAAHSQSFMD------FSTQVDYVLGIHDGVD-----
Mipuc02g00100   TDCSIVELGFCGGREAAGSNDADSIAWVLTGNEDAWAFDAGA-DLETLGSTLAHVPNTRAAAFSAAAQGASSDATASSSFSRGVDYLLGCFRVRPGEGEG
Mipur03g06080   TGCAIVELGFVSG---AGD--DD-VLWLLTGNEDAWFYDAGD-DADTIGNTLAYIPDTRGAAQRSS----FAADIHAGGLSQQVDYLVGCFSRK----EP
                : .::  :**      ...       *  ** *:*  : :   *   :*  *.:: ::*  .  ::             :   ***::* .

                       410       420       430       440       450       460       470       480       490       500
                         |         |         |         |         |  1      |         |         |         |         |
Ol03g00040      --PGELFLSAGTQSGVVGIFPLVQGSPSMRGTSAYVGLGAPAAVLRRGHRDIVRS-IVWDANATASESARVPATVGEDALVCAWTPDVGNEERP-----P
Od03g03130      --PGELFLSAGTQAGVVGVFPLVQGPSHARGTADFITLAPPVAVLRHGHRDIVRC-VAWDANARASEPSRVPLTCGEDSLVCAWTPGTNDDAAPPP---P
Ot03g00170      --PGELYLSAGTQSGSVGVFPLVQGPEPARGTAGYVILGPPVAVLRNGHRDIVRA-MSWDGNRHASEAARVPLTCGEDSLVCAWTPGAANAADPP----S
Mipuc02g00100   DGASSVVVAAGTQDGAVGLFPIVPPRDGSGD-VARCELAAPTRVLDGGHADIVRACVLWDWDADAAA----PATGGEDSRVVLWGAAGGAG-GGDG--SS
Mipur03g06080   GGGSTVMVAAGTQGGAVGVYPVVASTDPAS---SPAVLGAPIAVMDGGHVDIVRA-MAWKPDNAAEP----PVTGAEDSRMCAWGEKKAVDPGGDGGYIA
                   . : ::**** * **::*:*              *..*  *:  ** ****. : *. :  *      * * .**: :  *

                       510       520
                         |         |
Ol03g00040      VDRARPPG------------RRHSPY
Od03g03130      VDRTRPPG------------RRHSPY
Ot03g00170      VDRTRPLG------------RRHSPY
Mipuc02g00100   GGGKAARSESAETEYHDRGRRRHSPY
Mipur03g06080   AGGKIGRG--------DEGGRRHSPY
                 .     .            ******
```

b) Jumonji-1

```
                         10        20        30        40        50        60        70        80        90       100
                          |         |         |         |         |         |         |         |         |         |
Ot08g01040       MSAPT--TRNANHER--------------------PSTSQSAARRT----------------------------RTVDAAIEDARVFTPTLEEFADPIVY
Ol08g01020       MSARR--DVDARHAS----------------------TSEKDSERKPS--------------------------RTTTADIASAPTFRPTLEEFADPIAY
Bathy04g01120    MLSLN--DKDEDQEEGLPLKPTQNDFVNEEEEERIPRARSKRARRAPKAVYDNDFEAHEIQNAIKASKVITNRCRTDGSDIPLAPIFYPTEEEFEDPIAY
Mipur03g03040    MVAADGGAIASEPPTGRRAKRTIRAPTKFVDEDGFNDADLQRALKAS----------------------MKHVRRVSSTTVPECPVFRPTAEQFADPFAY
Mipuc02g01760    MAARS--KRLTRPPAKRRD----------ADDAMFDEAEVQRALRAS----------------------VKLQKRTSSTCMPSCPTFHPTPEQFRDPFAY
                 * :                                     .    :                            *.  : :  .  * ** *:* **:.*

                        110       120       130       140       150       160       170       180       190       200
                     Jumonji-N      |         |         |         |         |         |         |         |         |
Ot08g01040       LTKIEPLVRRTGICKVIPPRGAKPTWNEDVWRKDVSTFETKLQNVHKLSEGRLFQFGKSYT-KSGYKAMAMAFEKEWAEGRAD--FDACDVN--------
Ol08g01020       LSSIEARAREAGICKVIPPRGAAPRWNGEAWRRDDARFETKLQNVHSLSEGRTFQFGKEYA-KGEYEAMAKAYEERWAKERPD--VDANDAN--------
Bathy04g01120    ICSIQSKAEAFGICKIVPPDGYAPNFNRACCFGEKSLVETKHQNVNRLQQGESFPPGKTYVGLEKYKEMADTFEENYKEAHPETFKDIKDEDDL------
Mipur03g03040    IKSITPEAMPYGIAKIIPPEGWKPPFNEEAG-GDGIPFDTKLQTVNRLQEGLHFEDGERYT-RDSYRDMADAFKRKYLETHRRVADETERLRRENRGWSD
Mipuc02g01760    IKSITSEGIAAGIVKIVPPEGWRPPFNANAG-GGEIPFDTKLQAVHRLQEGVDFEDGKRYT-RESFRAMADSFAAAWMHAHLSLDAEVKRLMIE-RGFEE
                 : .* .     ** *::** *  * :*          .:** * *: *.:*  *  *: *.    :. ** ::   : . :     :

                        210       220       230       240       250       260       270       280       290       300
                          |         |         |         |         |         |         |         |         |         |
Ot08g01040       ------SVERAFWNMVETQEEKAA-------------------VEYGNDLDTKEFGTGFGVDAHG-----------------------------------
Ol08g01020       ------ALERAFWDMVETRSEQAR-------------------VEYGNDLDTKIFGTGFGVDENG-----------------------------------
Bathy04g01120    ----LKRIEDEYWRIVETNPNEAKAECGSLIQTKNVNKKGEVLVEYGSDVDARRFQSGFAAGISGD----------------------------------
Mipur03g03040    DACEARALEEEFWRIVETDVEKIR-------------------VEYGSDLDADVYGSGFAKVPLG-------------SASAAAGATPDSDSD-------
Mipuc02g01760    ADARARAVEEEFWRIVETNAERVS-------------------VEYGSDLDADVYGSGFGLYPRANYDGNDEISDDVMSRRADADGDGDGDGDGDGDGDG
                        :*  :* :***  :.                     ****.*:*:  :  :**.    .

                        310       320       330       340       350       360       370       380       390       400
                          |         |         |         |         |         |         |         |     Jumonji-C     |
Ot08g01040       ------------ERHPWDFEHLYSHPLNLLRVIEHDIPGLTKPWLYLGMLFATFCWHVEDHFLCSVNYLHTGASKTWYGVPGSDAEAFENCARATVPRLF
Ol08g01020       ------------EKHPWDFEHLYSHPLNLLRVVEHDIPGLTKPWLYLGMLFATFCWHVEDHFLCSLNYLHRGAAKTWYGVPGSDAEAFENCARATVPRLF
Bathy04g01120    --------PEDTEKHPWDMFELSKHPDNLLRFVDDDIPGLTTPWVYCGMLFATFCWHVEDHYLASVNYAHKGSAKTWYGIPGSDAEKFEAIAKTAVPSLF
Mipur03g03040    --------EDGGVPHAWDFGELIRHPSNLLRVVGGDIPGLTRPWLYFGMMFSAFCWHVEDHYLGSVNYMHAGAPKTWYGAPTHAADAFERAVRDIVPGIF
Mipuc02g01760    DGDCDSDSEDGARRHAWDFSELVNHPSNILRVVGGDIPGLTRPWIYFGMLFSAFCWHVEDHYLGSVNYLHDGAPKTWYSIPPASASAFERAVRTIVPTRV
                              *.**: .*  ** *:**.:  ****** **:* **:*::********:* *:** * *:.****. *   *. **  .:  **  .
```

```
                                 410       420       430       440       450       460       470       480       490       500
                                  |         |         |         |         |         |         |         |         |         |
Ot08g01040       QQAPDILHQIVTMVPPGILID-HGVKVVHTVQHPGEFIVTFPRAYHAGFSHGFNVAEAVNFGHANWLDHGRRAIDVYSTGSFKRNAVFAHHRLLARAAET
Ol08g01020       EQAPDILHQIVTIVPPGVLVD-HGVKVVHTVQQPGEFVVTFPRAYHAGFSHGFNVAEAVNFGHVNWLDFGRRAIDVYSTGSFKRNAVFAHHRLVSRAAET
Bathy04g01120    KENPDKLHHITMLVPPGQLIE-NKIKIVKLVQKPGDFVVTFPRAYHSGFSHGFNVGEAVNFAPVDWIEMGRVACRNYVKGNGKRNAVFAHDRVVVTAAKS
Mipur03g03040    KDAPDLLHRLVTLVPPAVLGEGHGVPVCQTLQRAGEFVVTWPRAYHAGFSHGWNVGEAVNFGTADWVPMGRAAVNDYQHGVGKRDSIFSHEKMILDTAKA
Mipuc02g01760    HDTPDLLHRLVTLVPPGVLRDAHGVPVFQTLQKPGTFIVTWPRAYHAGFSHGYNVGEAVNFGTAEWVPFGRAAVEAYVTSSFKRNAVFSHERVLLETGRR
                 .: ** **::. :***. * : : : : : :*:.* *:**:*****:*****:**.*****. .:*:  ** *   *  .  **:::*:*.:::  :..

                                 510       520       530       540       550       560       570       580       590       600
                                  |         |         |         |         |         |         |         |         |         |
Ot08g01040       FAEVLNAKGL------LLKSKVMGTVIATLCKELESIVSDEEIYRSSLVR--RG--------LKMEVVALPNEDD--DACCIRCKAIPFLSVVRCK-CLP
Ol08g01020       FVEVLGKNAR------LVKSKAMGAIVSTLRKELETILSDEEIYRASLVR--RG--------LNIEIVQAPNEDD--DACCIRCKAMPFLSVVRCK-CLP
Bathy04g01120    LKKIFETTK--------SRGKWMAHMSRVLRTDLETLADELENWQSILNGKQRGDGFIKGDPLRFYKCQNIPEMDGPEDCCVVCKAMPFAAVVRCE-CEF
Mipur03g03040    FVRRYGYGDGSSREDQSLRAPWIARMADALRAELQIIEKEQRAGRAVVTS--KG-----VKEVAGKENE-ASKHEDEDENCALCKAMPHLAVVHCARCFE
Mipuc02g01760    HARSFASPGGVSDEA---RAPWIASVARMIRDDLFTIAREQRTGRDAALT--RG-----VRVSADDDCLGGRVTHDHEVVCAECKSMPYLAVARCETCWK
                   .               :.  :. :   :  :*  :  : .  :       :*                    .  :  *  **::*. :*.:*

                                 610       620       630       640       650       660       670       680       690       700
                                  |         |         |         |         |         |         |         |         |         |
Ot08g01040       --TAVRCLRHAMDGCDCAASERCLEVRVMGSYIRGLLRSLLLGEPVEKAGGMAEEKDLEKFVASVKTVAVIRSPS----------TTAAPPASKKSKLSD
Ol08g01020       --TAVRCLRHAMDACDCAAGERTLEIRVVDSRLRELIKALFFGDGIQTKNDAAKAR--VDFSANVNRVAVNRAPPPKPKVVLPKPKTVKPPPTRAVLASP
Bathy04g01120    GRSFARCLQHWNRGCDCKQRHRMVEMRMEVDELRALAKSLEL----------------------------------------------------------
Mipur03g03040    IAEKIEFEQAVARARAEGRSAAGAAWALQGGSSSERLRRMGRYH--------------------------------------------------RRRRRNRP
Mipuc02g01760    LKDHIELSQAIARARAQGKSAAGAALALGNDRAKGAPYGGTTRE--------------------------------------------------GRRDLNRP
                      .  :    .             :  .

                                 710       720       730       740       750       760       770       780       790
                                  |         |         |         |         |         |         |         |         |
Ot08g01040       LDQTVEG--LQERIEER--QALLSRTEGGKVWTSERAKAFDKAAEQLGGMFKATSGDIGKILRDEYDIHASRDQIGSRLQKCRNKLRREQQSTDGDGV
Ol08g01020       PPTRIVASKADDAFTARGLPRKRAKCETRRRWTAEMVADFEVAVERLGGVDAATGKKLAEALS---AHDVTRDQCASRLQKHREKIKSNADARATL--
Bathy04g01120    ----------------------------------------------------------------------------------------------------
Mipur03g03040    VFCLG--HALDGNCGHG---------------VTELVMTTNASMDELERVLGALQK--------------------------------------------
Mipuc02g01760    VFCLK--HALGGACGHP---------------ARERVVNTRVDVREIEDLARALGEFVGPS---------------------------------------
```

c) SepA

```
                         10        20        30        40        50        60        70        80        90       100
                     2    |        |00       |         |         |         |     00  |  00     |         |        2 |
SEPA_PHYPA      M-SRHTSGSAFHKSKTLNDKYMLGDEIGKGAYGRVYKGLDLENGDFVAIKQVSLENIPPEDLASIMSEIDLLKNLNHRNIVKYQGSFKTKTHLYIILEFV
MAP3K_ARATH     M-ARQMTSSQFHKSKTLDNKYMLGDEIGKGAYGRVYIGLDLENGDFVAIKQVSLENIGQEDLNTIMQEIDLLKNLNHKNIVKYLGSLKTKTHLHIILEYV
SEPA_DICDI      M-SKKEP-EEIKKNVTVGN-YNLGVVIGKGGFGTVYQGLDIEDGDFVAIKQINLTKIPKDQLQGIMNEIDLLKNLNHANIVKYIKYVKTKDNLYIVLEYV
Mipur05g06740   MELPRSTRRS-----TLVGSYILGDEIGKGAYGQVYKAIDKRDGRVVAIKEIPLAGIDEASLAGVRLEIDLLGSLSHPNVVGQLGTIRTPSYFYIVLEYC
Mipuc16g03100   MELPRSTRRS-----TVVGNYILGDEIGKGAHGQVYRAIDKRDGRVVAVKEIPLRATSAYDVDAIESECALLRSLSHRNVTRFLGTVRAPEHLYILLELA
Ot17g01940      M--PQTDPQP--HPPRVVGHLILGELIGVGATSSVYKAVDQRDGRVSAVKEISLIGVDSQDMERITAELELLSNLEHANVVKYEGARTIGESLYVELEFA
Ol16g02130      M--PASPPASPHHAVKRVGNHILGELIGSGATSRVHKAVDQRTGSICAVKEIPLRGVPVEQLERITSEVELLSRLEHANIVKYEGAVRVEECLYIMLEYA

                        110       120       130       140       150       160       170       180       190       200
                          |         |       00|         |         |       00|    2    |         |         |         |
SEPA_PHYPA      ENGSLANNIKPNKFGALPENVVGRYIAQVLEGLVYLHE--QGVIHRDIKGANILTTKEGEVKLADFGVATKLTEADI-----------------------
MAP3K_ARATH     ENGSLANIIKPNKFGPFPESLVTVYIAQVLEGLVYLHE--QGVIHRDIKGANILTTKEGLVKLADFGVATKLNEADF-----------------------
SEPA_DICDI      ENGSLSGIIK--KFGKFPETLVCVYIRQVLEGLVYLHE--QGVVHRDIKGANILTTKEGKIKLADFGVATKFDDT-------------------------
Mipur05g06740   EAGSLAASIKANKFGPAPEALCKVYVAQVLDALAYLHSPRNGIVHRDVKGANLLATKDGCVKLADFGSAARMGEDGR--------------GGAQRPGSN
Mipuc16g03100   ENGSLAGVIKPSRFGPTPEPLAACYVAQLLDGLIYLHA--NGVTHRDIKGANVLATKDGVVKIADFGVAVRVHGGAA--------------NPPVNPAHH
Ot17g01940      ENGSLARTVHPSRFGGFPESLCAVYVAQILRGLAYLHG--QGVVHRDIKGANILTTKEGVVKLADFGVATKGSRVGGGDGLGRRFGLLN--EASRKIASM
Ol16g02130      ENGSLARTVHPSRFGAFPESLCAVYVAQVLRGLAYLHS--QGVVHRDIKGANILTTKEGVVKLADFGVATKGGRASG-DGLSGVFGAEGRSEGSDGDASD

                        210       220       230       240       250       260       270       280       290       300
                          |         |    2  00          |         |         |         |    0    |       2 |         0
SEPA_PHYPA      --------------NTHSVVGTPYWMAPEVIEMSG--VSAASDIWSVGCTVIELLTCVPPYYDLQPMPALFRIVQ-DDHPPLPEHVSEVIIDFLRQCFQK
MAP3K_ARATH     --------------NTHSVVGTPYWMAPEVIELSG--VCAASDIWSVGCTIIELLTCVPPYYDLQPMPALYRIVQ-DDTPPIPDSLSPDITDFLRLCFKK
SEPA_DICDI      --------------SAAAVVGTPYWMAPEIIELNG--ATTKSDIWSVGCTVIELLTGSPPYYDLGQMPALFRIVQ-DDCPPLPEGISPPLKDWLMQCFQK
Mipur05g06740   KP---TKKTTGPVDGEGDVVGTPYWMAPEVIEMSG-GSDPKSDVWSVACVVVELITGSPPYFDLQPMPALFAIVR-DESPPLPPGISPELRGFLSACFRK
Mipuc16g03100   PA---RSVDDDDGDPGASPVGTPYWMAPEVIEMRGPGDDPKSDVWSVACVVIELLTGAPPYFEMQPMPALFAIARGDARERIPPGVSDALRDFLSRCFEK
Ot17g01940      EN--SEEANADGEAKPNDALGTPYWMAPEVIEMRN--VTAAADVWSVGCTIIELLTSNPPYYDLDPMPALFRIVR-DKHPPLPAGISDALRDFLMLCFKK
Ol16g02130      GRGIGDGTRAEGEGEGDKALGTPYWMAPEVIEMRS--VTAAADIWSVGCTIIELLTSNPPYFDLDPMPALFRIVR-DEHPPLPTGISEALRDFLLLCFKR

                        310       320       330       340       350       360       370       380       390       400
                0         |         |         2    2    |         |         |         |         |         |         |
SEPA_PHYPA      DAKRRPDAQTLLGHAWIRKSRREKRNGVVSHGIAH-------------------------------------------------------------FPRL
MAP3K_ARATH     DSRQRPDAKTLLSHPWIRNSRRALRSSLRHSGTIR-------------------------------------------------------------YMKE
SEPA_DICDI      DPNLRISAQKLLKHKWIQASIKKKPVENGAGGVGNGTDSLGAPANIDDIAKNITDYNERINKKPSHQRKPSIHPKSPKGKVFLPPPEEEEDEWGDDFSNT
Mipur05g06740   DPAQRPTASELRSHEWLKGVATAGAATGSSSG--------------------------------------------------------------------
Mipuc16g03100   APERRPSAAELRRHAWLRDVATPGTTSDAASRAID-----------------------------------------------------------------
Ot17g01940      DPKDRPAAEALLSHTWLTDE--------------------------------------------------------------------------------
Ol16g02130      DPKDRPSAEELINHTWLMDEHKVLAETWTKR---------------------------------------------------------------------
```

```
                        410       420       430       440       450       460       470       480       490       500
                          |         |        00         |         |         |         |         |         |         |
SEPA_PHYPA       PGS----HDQDLLETYMSTTAIRVPPTVTTSLTRPPAGSRVSESPEP-LVHNTVLRRTSGGPEEHAQCEAN------VMRSTSGKFS---------HLLP
MAP3K_ARATH      TDSSSEKDAEGSQEVVESVSAEKVEVTKTNSKSKLPVIGGASFRSEKDQSSPSDLGEEGTDSEDDINSDQGP---TLSMHDKSSRQSGT-------CSIS
SEPA_DICDI       PKSIKLPDKKSPLKLTNNKPSTPLKQQPTNNTPVQQQQQQQQPPPLKLAVPKQPVIENDDDWGDDFNTVSDLSKAVGSLNFNNNKKNETPKPNIKKPTFS
Mipur05g06740    ---------PARRTEHLTLDDPRPDSAASSLAGSGRSSPAHSRPTSRGAQRRGSRGDGGDGAWEPPASIAD----------------------------P
Mipuc16g03100    ---------DVVVEETVVASVSRVNSGGSTSGMSGGGGGGGGGGKTKNQNQNQNQNHRGDAPSKPTPRRPS----------------------------P
Ot17g01940       ------------------RSLTEQPRE-----------------VGALIDRVANIGVEAST---------------------------------------
Ol16g02130       -------------DASGGRTQNDDAEEG---------------VIVKVIDQMAKVGVNGDVTAADDAAAAN----------------------------S

                        510       520       530       540       550       560       570       580       590       600
                          |         |         |         |         |         |         |     00  |         |         |
SEPA_PHYPA       GQKDAELKDSLSGTEICTWKNDIHGMKTEVN-G---------NHVSTQVSLSYLHPFVFV----------ELSLWCAVKPSTVHS----SKFTDGPLDDN
MAP3K_ARATH      SDAKGTSQDVLENHEKYDRDEIPGNLETEASEGRRN--TLATKLVGKEYSIQSSHSFSQKG---------EDGLRKAVKTPSSFGGNELTRFSDPPGDAS
SEPA_DICDI       EDEDEDDDDDGFGSGGDDEDDDFGDIPTSIKLNPKFGSNIKGNSSGSANTTNSSSTVVQQPKLTVSNNNNNNNKKLPLSPRQPSSGNVKEGINHGSTGSK
Mipur05g06740    QP--RERRRRRPP-----PSSPFQDVKVAMN----------------GDADADADEVADE----------------------------------------
Mipuc16g03100    SSPFQDVGKKQPVRIVRGVAANLDDVVVALRPEERS--------ASLAASSSSSSSVASDG---------------------------------------
Ot17g01940       ----------------MKRASSSSDALNLLG-------------------ISTSNGMTKE----------------------------------------
Ol16g02130       SP-------LIAA---SERSSSQKDLENFMKSD-------------LGNFMKSDSGVTKA----------------------------------------

                        610       620       630       640       650       660       670       680       690       700
                          |         |         |         |         |         |         |         |        00    2    |
SEPA_PHYPA       TNGLFFG--------SHEASPSVP-AVPNHGLPPRGGPSLEIHATAAKMKAKTAQSQAEAESLLTAKTN-GYESSIDLDDDDSVRTGLVSYVDKILMYKC
MAP3K_ARATH      LHDLFHPLDKVPEGKTNEASTSTPTANVNQGDSPVADG--GKNDLATKLRARIAQKQMEGETGHSQDGGDLFRLMMGVLKDDVLNIDDLVFDEKVPPENL
SEPA_DICDI       SGGVIIDQWG---EDGEEDNDWGDVATVNFDPKVIRKGTVNKPDLSTRLKNRIALSETALSNSFNNNGNDDEDEDIFADDFDEDDDEDFDLDKNLMKDNY
Mipur05g06740    --------------------------------------VAAPAAVSVRL--EYPNTLNTLNTGFDGDVA---RATRDARWSDLAGLVRTAVADGVTAAAV
Mipuc16g03100    ----------L---------------------KTAKAPTAAPTEEEVRAGDRLASTVKRIAATLRASGS---GSIDDTAGKGKGARTPDALAGGFARACQ
Ot17g01940       ---------------------------------------------QIDLNETIVSVMDRLRSAF----------------ADTSSSENVEMAG-------
Ol16g02130       ---------------------------------------------QIDFNKSLISIVDRLKDAFGTS----------T--SDVAAIDDAEQAG-------

                        710       720       730       740       750       760       770       780       790       800
                     00   |         |         |         |         |         |     00  |         |         |   00    |
SEPA_PHYPA       I-VQALEVSRLMAMLKLDETEEVILAACQKLSSIFREFPKQKQDFMKPHGLIPLMDMLDMN--NNRVIHAVLQVLNLITDDNVELLESACRAGLIP-IMM
MAP3K_ARATH      FPLQAVEFSRLVSSLRPDESEDAIVTSSLKLVAMFRQRPGQKAVFVTQNGFLPLMDLLDIP--KSRVICAVLQLINEIVKDNTDFLENACLVGLIP-LVM
SEPA_DICDI       A-RMSSEILKLMNLLTPEQPEEVISSACTQLITMFKENSEQKTLLIRRHGVIPIMEMLEVSNIQSHVLCSILKVVNQIIDNNMEIQENLCLVGGIP-AIM
Mipur05g06740    D-AALTSADTAGALAAALHAAASKDGDDATAVRERGNNIDDANAAASLDAAAAVLAATRRK-------------RADGGDAPGETLAAFVTFGAASSTLS
Mipuc16g03100    D-LVDALARERGATRVSVDACVAANGVMGALVGALEASSPAAAATAAATAAAAVIDRANRG-------------GGGSGSGS-EALATFELLGGIPLIME
Ot17g01940       --ADFVQLMDSSTSFNVTNETLFTTRANCTFMNVINDDAAAETTRTVAFECVAAASAASR-----------------------DVLTSFVTMGVLPRALE
Ol16g02130       --AEFVRAMETATSVLVANETLFTTRACGVFVNVLENESASARMRTVALECVTAASMLSR-----------------------DILTSFVTLAVLPRALE
```

```
                        810       820       830       840       850       860       870       880       890       900
                          |         |       2 |         |         |     2   |         |         |         |         |
SEPA_PHYPA       SFASP--ECSKEMRMQAAYFVQKICHKSSLTLQMFIACRGLPVLVGLLEKDYARHRE-MVHMAIDGIWQVLELPGLSLKNDFCHIFARSSILARLVDTLH
MAP3K_ARATH      SFAGFERDRSREIRKEAAYFLQQLCQSSPLTLQMFISCRGIPVLVGFLEADYAKHRE-MVHLAIDGMWQVFKLKKSTSRNDFCRIAAKNGILLRLVNTLY
SEPA_DICDI       KFSGP--EYPASVRLETASFISKMCSTSTLTLQMFIACKGLPILVDFLLSPYAESKR-LVWMAVDAIVNVFELQSPTPKNDFCRLFSKCGLLK-------
Mipur05g06740    LLRSDDASVGVGVKIAAAKVVRELSRGGVVALRCLASCDAPRALAGVLAQSAMSPS--RRELARVCVDVARFLTTLHTRAVTGSIPPSEASRMAS-----
Mipuc16g03100    RLARQTGPETRESRVACAKAARSVAAGGAVARRCLAACGAPKALAATLSSPGFYSGEGNRELTRACVETCAEMMRAHEDEERGRGGGGEGVDFSGRR---
Ot17g01940       VLRRENASAR--AKIAALRLCRVTAKAGPVFSRCLVSCGALPILVGVLDEGFGGSGRELTKYALSAIYIAAEIERGDQMPRQLGQAISLSLAQAG-----
Ol16g02130       VLKKKDSSMR--IKLAALRLCRATAKAGPVFTRCLVACGALPILVDVLDYGYSGNGRELTKYALGAIYIAAEIERGDQLPKQLAQTVSLALASAG-----

                        910       920       930       940       950       960       970       980       990      1000
                          |         |         |         |         |         |         |         |         |         |
SEPA_PHYPA       TLNEASRVP---------NGSMTTEQSRPLSSEK-AFVKSQSGP--IDQSRIVN---------KEVFRSRSGQLD-HTPVRVTHDGN-------------
MAP3K_ARATH      SLSEATRLASISGDALILDGQTPRARSGQLDPNNPIFSQRETSPSVIDHPDGLKTRNGGGEEPSHALTSNSQSSDVHQPDALHPDGDRPRLSSVVADATE
SEPA_DICDI       -------------------------------------------------------------------------------T-LP-----------------
Mipur05g06740    -----------------------AASSGGISSGDVDFDEDDGETR-----------------------GGGVVFDAFARAGLIPALV-------------
Mipuc16g03100    ------------------KKPDDAAAAATTTTTDDDAIEDVPAGRT----------------------AGWGMRVAIARAGLLPPLV-------------
Ot17g01940       ---------------------LFPKLVALLGSTHTASLEDEANP-----------------------------EVMDDTRSEG-----------------
Ol16g02130       ---------------------LFPKLVTLLGATHTASLEDAAMIT----------------------------DDIDDARSEG-----------------

                       1010      1020      1030      1040      1050      1060      1070      1080      1090      1100
                          |         |         |         |         |         |         |         |         |        1|
SEPA_PHYPA       -----QRNLLGLNNGDSSRMLPWQAHRACGL----P------------EFSWQNLPGQLEYTGQHSG---HLTHMKLAISSE--RLSDPFQPGYGESTWV
MAP3K_ARATH      DVIQQHRISLSANRTSTDKLQKLAEGASNGFPVTQPDQVRPLLSLLEKEPPSRKISGQLDYVKHIAGIERHESRLPLLYASDEKKTNGDLEFIMAEFAEV
SEPA_DICDI       ----------------------------------------------------------------------------------------------------
Mipur05g06740    ------NAIRDLNAAANE-----ERGVGPG----------------------------------------------------------------------
Mipuc16g03100    ------RALSSLHAASNE-----EAGVGPGGGG-------------------------------------------------------------------
Ot17g01940       ------------GSS---------MTGSIG----------------------------------------------------------------------
Ol16g02130       ------------GSS---------MTGSIG----------------------------------------------------------------------

                       1110      1120      1130      1140      1150      1160      1170      1180      1190      1200
                          |         |         | 1       |         |         |         |         |         |         |
SEPA_PHYPA       LGGDGCRRERDDEARSD--------------ANTSSSTSQTTSGQLS-------R---------LQETHNPEEAQEYLGKVANLLLEFSRAD-----TAV
MAP3K_ARATH      SGRGKENGNLDTAPRYSSKTMTKKVMAIERVASTCGIASQTASGVLSGSGVLNARPGSTTSSGLLAHALSADVSMDYLEKVADLLLEFARAE-----TTV
SEPA_DICDI       -------------------------------------------IVLR------------------DSIADGEAAATYPDRIINLFIMFSAAD-----SVV
Mipur05g06740    --------------------------------RKATNDTEKSESEKS-----------------------ESPSSVYRERVADILLDATRSRNDRGCAAS
Mipuc16g03100    --------------------------GFGGLEKNSAAAASKTKTKSSPLAGRNANPD-------LPPHVAATVSGTCRDLVADILLSATSPG--HGARDA
Ot17g01940       ----------------------------------GTSTHSVSRGLYS------------------------SGGRYYRELVAETLYRVAKRG--DGCPEV
Ol16g02130       ----------------------------------GASTISRSRSGGS------------------------SGGKYYRELVAETLYRVAKRG--EGCAEV
```

```
                      1210      1220      1230      1240      1250      1260      1270      1280      1290      1300
                         |         |       00|         |         |         |         |  00     |         |         |
SEPA_PHYPA      KKHMCSTSLLQRLLQML-NTLPPPILIKILECIKQLSQDPFTLEYLQLAEAMKHLIPFLEARDGPYVGRIHNQVLNALHNLCKIN---------------
MAP3K_ARATH     KSYMCSQSLLSRLFQMF-NRVEPPILLKILECTNHLSTDPNCLENLQRADAIKQLIPNLELKEGPLVYQIHHEVLSALFNLCKIN---------------
SEPA_DICDI      RKTMSAVEVIRPILDTL-SQLMPEQLAKVLKSIKQLSMDHNTLANLQNAGAIRFMVPFLGRRTGAFVAEIHNHVLNTMFHLCRID---------------
Mipur05g06740   RFALCELQAMHGLLALAGSPLPKSTSAKLLRVVGHLARDDNCKDAMQRAGAVPKLVRFLQWEDPA----TREEALRALYNLCRG----------------
Mipuc16g03100   IEALCETSTLHGLLALVGAPLPASTSKKILALVRRLARERNAHEPMQRAGAIPKLARFLQWEDDD----HRETAMVALFHLCGGGGGGGGGEDAGAASSA
Ot17g01940      SKSLVDVRIIHGCLAQL-SVVPRSTATKLLGLVHLLSKQPDSFDVLQHSGAIPKLVKCVEGNVKSGHAQSWELALKALHNLCAMN---------------
Ol16g02130      SKALVDVRIIHGCLAQL-SVVPRSTATKILGLVHLLSQQTNAFHVLQNAGAIPKLVKCVEGNFRSGHAQSWEMALKALHNLCAVN---------------

                      1310      1320      1330      1340      1350      1360      1370      1380      1390      1400
                         |         |         |         |         |         |         |         |         |   2     |
SEPA_PHYPA      ------KRRQEQAAECGIIPHLMHFIKI-------------------------------------------NSPLKQFALPLLCDMAHASRTTRELLRTN
MAP3K_ARATH     ------KRRQEQAAENGIIPHLMLFVMS-------------------------------------------DSPLKQYALPLLCDMAHASRNSREQLRAH
SEPA_DICDI      ------PERQYQAAIDGIIPHLQYFITS-------------------------------------------HSPLNQFALPIICDLAHS-KKARSELWKN
Mipur05g06740   -----DASALEQAAVAGVTPHLIAVAAPDVFN--------KLGLADELGAATGTNGGGTHWTRGGPGAEAQIERLAPLAAPFLCDMASSSRRTRGELARH
Mipuc16g03100   AEDPGVAARREQAAIAGAVPHLVAVATPEIAHGDATVDVSSSAASAGRGVVNGLHGDGDHWRR----HPLSAAKLRALASSMLCAFAYSSRKTRLELSKH
Ot17g01940      ------KERQEQAAVAGLIPILVKIVA----ERN--------------------------------VAHTVQQSAMKLAVPLLCDMASASRKTRELLEKH
Ol16g02130      ------KERQEQAAVAGLIPILVQIIASARDEKNG-----------DGSMSTATNGDKVNTAA---VATTARSSAMTLAVPLLCDMASTSRKTREILEKH

                      1410      1420      1430      1440      1450      1460      1470      1480      1490      1500
                         |         |         |         |         |         |         |         |   2     |         |
SEPA_PHYPA      RGLDFYLSLLDDEVWAVT-------ALDSLAVCLAHDNEQRKVEQALLQKDALQRLVAFFQSCG----APSFVHILEPFLKIISKSVRLNTALAVSGLTP
MAP3K_ARATH     GGLDVYLSLLDDEYWSVI-------ALDSIAVCLAQDVDQ-KVEQAFLKKDAIQKLVNFFQNCP----ERHFVHILEPFLKIITKSSSINKTLALNGLTP
SEPA_DICDI      NGVAFYLSLLEERYWQVN-------ALDSLAVWITDETHK--VENIIATNENIKKLIQLFTNAE----SQSFAGILEPLLKIIQISIPVNILLGTSNFIT
Mipur05g06740   DALDAYLSIARNKPSASSPPGLQLAAVRAVAGWMRDEPWK--VEARLAEPDAIAAWASAIDPRR---IPPIDPEVLDTLREIARESPRLCAALASGGAMA
Mipuc16g03100   RALDAYLSLVASGEGGAG----SATALRAVSAWQRDEPWA--AEARLMEPDAVASIAAALQPRPGDVLLPCD-AFLTTLLGLLKRSPRVCAGVAQGGGMA
Ot17g01940      GALDAYVELISVESGWTLS------ALNAVGSWLAIEPWK--AEARFLEPDAINSILDVLDEST-----SQD-NIMQALLNLISTSPRLCQALANEHFIP
Ol16g02130      GALDTYVQLIAVNSGWTLS------ALNAVGSWLAIEPWK--VEARLLETDAIDSILEVLDEST-----SQA-NVLEALLDLISKSPRLCQALANEDFIP

                      1510      1520      1530      1540      1550      1560      1570      1580      1590      1600
                         |         |         |         |         |         |         |         00        |         |
SEPA_PHYPA      LLVARLENQDA----------------------IAR------------------------------------LTLLKLIRAVYEHHP-RPKQLIVEHDLP
MAP3K_ARATH     LLIARLDHQDA----------------------IAR------------------------------------LNLLKLIKAVYEKHP-KPKQLIVENDLP
SEPA_DICDI      KIIDKLGHTNP----------------------QVR------------------------------------LNLLKIITSLYECHP-NAKKMIQEFKLI
Mipur05g06740   PLVEALGAPSSATAAVSSRATHNAGTVDWNNGAVLRRSNSRRLELRPGRAGAGSRTDDPSRPHDARKDPRRTLAYVRLLAQLADHRRDAVDESGRGHDLV
Mipuc16g03100   PLVEALGPPSSSAAAASGLSRSSS-------GGAFR--G-RLLETHPG----------------------RALLFLRLLAVVYEHHP-RREEAAAGYDVR
Ot17g01940      PLMDALTTAAT--------------------KPTTR------------------------------------ITLLKMLGVVHEHAQ-RPKELIIRYDVV
Ol16g02130      SLMDAITSPTT--------------------KPTIR------------------------------------ITLLKTLGVVHEHAQ-RPKELIIRHDVV
```

```
                1610      1620      1630
             00    |         |         |
SEPA_PHYPA      TKLQRLIEDRRDGERSGGQVLVKQMAYSLLRALHINTVL
MAP3K_ARATH     QKLQNLIEERRDGQRSGGQVLVKQMATSLLKALHINTIL
SEPA_DICDI      PIIQKIADT--DKS-----VLVQKMASKLLEAFNANTVI
Mipur05g06740   GRLRGVDDESADSERAAEDVR--TEAERLLAELRR----
Mipuc16g03100   ARLRGVLERGGGGGREAAAAV--DVAEELLRKMR-----
Ot17g01940      ARLKRLIEGVTDER-SSGAVA--QLVDKILRSMRLTRVA
Ol16g02130      ARLKSLFTDHGDERQSHTVIA--QLVDKILRSMRLSRVV
```

d) U5 Helicase

```
                         10        20        30        40        50        60        70        80        90       100
                          |         |         |         |         |         |         |         |         |         |
Mipuc05g04870   MG--GGAEGKRGAAAP------PRLSTFLRTFTLATTND--------ARDAVDVDAEFVARAAILARAGDGGAGGHG-AGRYHATASLAARVLGGSDASP
Mipur08g03240   MG--HGRDGVSGGSQSGSTRPAPRLSALLRSLATADANE--------DVDALYVARRGALDAAIASGSATGPAPGRGGKGRYHATSSLAR---------L
Ol08g01930      M--------ATTHAVA------PRLTTFLRAARG-------------LDDVVDVDAERARRNETLRTTTRRDATTD-----DTPSVRLAR---------R
Ot08g01860      M---------SSIVVR------PRLTTFLRASRAS---S--------VDDIVDVEAERARRTRGFEGVTRGGGERER----EGPSARLAS---------V
Bathy01g04990   MAPLSTSSSSSSSSSSSSRRPPPRLTTFLRARCGGKSGDGSDDSIDRYWYVSDVDEAFVARAKQIGSATTKTSSPSLSFRQTAKKATKNRDLALLKLSDV
Q9SYP1_ARATH    M------ANLGGGAEAH-----ARFKQYEYRANSS------------LVLTTDNRPRDTHEPTGEPETLWGKIDPRS----FGDRVAKGRP------QEL
A9RTW1_PHYPA    M------AHLGGGAEAH-----ARFKQYEYRANSS------------LVLTTDTRPRDTHEPTGEPESLYGRIDPRS----FGDRVYHGRA------NDL

                        110       120       130       140       150       160       170       180       190       200
                          |         |         |         |         |         |         |         |         |         |
Mipuc05g04870   PSPSSPSSPSPSSSRDPPKDARDALDAFLPVVVKVT--GGDTLLPDQLADASLVAFDAIAGLCEESIAAAERWDEETTYGRDP--DLAKKKAAAE----E
Mipur08g03240   ICPASPDSP--------PREVRAALERFLRAVVRVT--GGDTLLPDALADASLLAFRTVASLARDANAAAARWAEEERYGRDA--DLAKRRGQAE----R
Ol08g01930      VLRCEDGRE--------AKDVVKTCRAFVSVVVRML--GSDTMTETESGDAAASAWDAASGFVEVIANERRAVESERGYVAD--HETARKIFEVR----E
Ot08g01860      VFGNDGSDR--------DKEVASACRAFMSAVLRAL--GGDTMTEDALGDAVVAAWEVLRDNVETLGRERRDLEERQPYVDDAVFETERKLFEVR----E
Bathy01g04990   ALKTSSSSLED-----FDEETRLALDLVADASIQLLRLENDLISPDELLEAPADLYAAVATNCDVDWKLEADLEDVVEYSNAP--ELERKKVQGKRRGNE
Q9SYP1_ARATH    EDKLKKSKKK--------ERDVVDDMVNIRQSKR-----RRLREESVLTDTDDAVYQPKTKETRAAYEAMLGLIQKQLGGQPP----SIVSGAAD-----
A9RTW1_PHYPA    EEKLTKHRRKR----EVKEKEKGSNAEGLKKARKRL---RGMQEESVLSIVDDGMYRPKTKETRAAYEALLSTIQQQFGDQPQ----DILRGAAD-----
```

```
                       210       220       230       240       250       260       270       280       290       300
                         |         |         |         |         |         |         |         |         |         |
Mipuc05g04870   RERARRMKTALAKALGPVDE--KHVPDLIDVAKRLVHIQKKRKGTSDDRDVDASAAEFGAAAVPARREFGADVVFNAP-RSRAVGSRASTSASTLIPRPA
Mipur08g03240   RDFMRRKRAALVAELGPVSD--QHLDEFDAAVNELIALQDAHGGGPGGDGANDGDGDD-G------YEFGSDIPFAAPGRGRAPASSSSR-TSS------
Ol08g01930      REVRKDMMEALRGALGPVEANAPTVREFESLVEDLLRMRGE-FDEFHTLQSTLDAEPTTSG-----RIFGQNIKFKRA--GDAQPSDRIR----------
Ot08g01860      RELRRDFGEKLRGALGPIEASAATLRELEQHVETLLKFRAERITDLDDYMIASSAAPSTSG-----RSFGHNIKLKCR--GDVQPSDRVR----------
Bathy01g04990   REAFGFVKARLGGVLSETGEAAQRWKDFWQSANALRRVGSTSSTGGDLNGTSMGVGGKKE------TYYGEDMFFVPPRFYGANDFDSSN----------
Q9SYP1_ARATH    -EILAVLKNDAFRNPEKKMEIEKLLNKIENHEFDQLVSIGKLITDFQEGGDSGGGRANDD------EGLDDDLGVAVEFEENEEDDEESD----------
A9RTW1_PHYPA    -EVLGVLKNDRFRDLDKKKEIEKLLNSMSNERFAQLVAIGKLISDYSEGGDAGAEGAG--------EALDDDIGVAVEFEEEEEEDESDY----------

                       310       320       330       340       350       360       370       380       390       400
                         |         |         |         |         |         |         |         |         |         |
Mipuc05g04870   PAASASAAASASAAAKLAATMKAGLRAAGEAEAEKRRGENAGTSP-LDASASRATSFGPLPPAEQSANEQLRWLNEKCVEHVGG---V-AGAGWEDVAQG
Mipur08g03240   ---SAQAPASTSSAARMAEQMRAGLLAATVSDADGKKSELYGAS---DANSASEGEFS-IPAAEASATAQLAWLKERCVAISG-------ANGWEETAQQ
Ol08g01930      ---------------DLKRAMKTNLSEASEAERLRIESERMAHPDE------RMSGFGEPEHVESDATTQLTWLKRQCEGFVANSAHA-LDQTWEAVAGT
Ot08g01860      ---------------DLKRAMKANLDELDEVARARIKSEKISHPN---------ELFGEPQREESDVTTQLTWLKRQCENFVANGAHA-LDQSWEAVASA
Bathy01g04990   ---QHCDQISSGLESTILSNMTRGLDSANAFASLELERSRTPSFHELDALG---TESEVSTSLQAGSGSILSWLREQCEIFRENNQNSDAFAGWTDICGN
Q9SYP1_ARATH    --------------PDMVEEDDDEEDD-EPTRTGGMQVDAGINDE--------DAGDANEGTNLNVQDIDAYWLQRKISQAYEQ-------QIDPQQCQV
A9RTW1_PHYPA    --------------DEVQEESDGEEGDGQDTRQASAMQMGGQDDE--------DMEEADEG--LNVQDIDAYWLQRKISQAHG--------DIDPQQSQK

                       410       420       430       440       450       460       470       480       490       500
                         |         |         |         |         |         |         |         |         |         |
Mipuc05g04870   VARATLSDASR-SDDDVAAELFDLLGDGGVELIMGAIERRVAMCAALRKRITTLRETLGGGKDDDGEDRDRAAAGGPGRTVTISSTTDKQIEKLRRKEER
Mipur08g03240   LARALLAAETR-SDDEVAAELFDLLGDGGVETIAGAIERRAAINAALRRRIGTLRETLGG-AGGDGDGGGGARKDAPMAQVTIQSTSDIKMEKLRRKEER
Ol08g01930      VGRAIMNASS--SDDECAAELYEYLGDYGFELIAGVVERRTVLTSAIKNRAQLLRDALNAQSGADRA------GPSITRVVTITSTLDKQLEKARRKEER
Ot08g01860      VGRSIMNSTI--SDDACAAELYEYLGDYGFELIAGVVERRTELASAIKKRAQMLRETLSAQSGVD--------GPSVARVVTINSTLDKQLEKMRRKEER
Bathy01g04990   ICRVCLNTSS--SDDQVASELFDLLGDGGFDLVASVCERRGQLADAIRRRLQALKEAFDPEESAQDD------YAKSKSAVSVRSTTDVAMEKIRKKEER
Q9SYP1_ARATH    LAEELLKILAEGDDRVVEDKLLMHLQYEKFSLVKFLLRNRLKVVWCTRLARAEDQEERNRIEEEMRGL--GPELTAIVEQLHATRATAKEREENLQKSIN
A9RTW1_PHYPA    LAEDVLSKLAEGDDREVENRLVILLDYDKFDLIKLLLRNRLKVVWCTRLARAEDEDARKKIEEEMSNG--GPVLAGILEQLHATRATAKERQKNLERSIR

                       510       520       530       540       550       560       570       580       590       600
                         |         |         |         |         |         |    2    |         |         |         |
Mipuc05g04870   KVGRRIAQGQGEPLLEWLAA-SGVGFGVLCEGDWEAAAASGGGR-GEDDVFAGLR--VGGGSGGRKALPAGTTRKVH-KGYEEVAVPAAKVAPVGDAERF
Mipur08g03240   KVGRRIAQGQGEPLLEWLAN-SGVGFAALCEGDWEAAANQPS---TEDDIWAGLYGLGGGGGGGKKALPAGTTRKVH-KGYEEVHVPAGERAPVGEHERF
Ol08g01930      KANRKLASGSGASIMEWLQA-VGVGFDALCEGDWENQQAPSSSS-NPDDILAGLRGLGRGMDGGRKALPPGTTRIVHPEGYEEISVPAREPDPVAAGERS
Ot08g01860      KANRKLASG--ENVMEWLQA-VGVGFDALCEGDWENQQTPSSSS--PDDILASLRGLGSGLGGGRKALPPGTTRMVHEQGYEEISVPARDPGAIGEGERS
Bathy01g04990   RNKRRAAGGHGEYLLEWFTNDSGLGYGAFCDDLLPLPNRNEAAPGSVDDILNSLRGLGLGTDGGKKALPPGTTRKVL-EGYEEIYVPARIPDAVADGELQ
Q9SYP1_ARATH    EEARRLKDETGGDGGRGRR--------DVADRDSESGWVKGQRQMLDLESLAFDQG-GLLMANKKCDLPPGSYRSHG-KGYDEVHVPWVS-KKVDRNEKL
A9RTW1_PHYPA    EEAKKLRDDGGEAADRGRRK---D--REVGVGGGESGWLKGQRQLLDLEQLTFHQG-GLLMANKRCELPPLSYRTPK-KGYEEVHVPHLKPKPFAEGEEL
```

```
                    610       620       630       640       650       660       670       680       690       700
                      |         |         |         |         |         |         |         |         |         |
Mipuc05g04870  VAIEELDDWAQLAFAGMTSLNRIQSKIYPAAFRSNENLLVCAPTGAGKTNIAMLTVLHEIGAHFDDDGEWNGD--DFKIVYVAPMKALAAEVTNAFSRRL
Mipur08g03240  VPIEELDDWAQPAFAGMKSLNRIQSRIYEAAYHSNENLLVCAPTGAGKTNIAMMTVLHEIGQHIEYGELAYGA--DFKIVYVAPMKALAAEVTGAFSRRL
Ol08g01930     VAIEELDEWAQPAFQGIRMLNRIQSKIFPQAYHTNENLLVCAPTGAGKTNIAMLTVLHEIGLHIDENGDYLPE--DFKIVYVAPMKALAAEVTDAFSRRL
Ot08g01860     VAIEELDEWAQPAFKGIKLLNRIQSRIFPTAYHTNENLLVCAPTGAGKTNIAMLSILHEIGLHIDENGDYLPE--DFKIVYVAPMKALAAEVTETFGRRL
Bathy01g04990  VSVSYLPAWAQTAFKGIQTFNRIQSKIFECAYTSNENVLVCAPTGAGKTNIAMLCAMQEIAKHFDEENNCLHEHDDFKIVYVAPMKALAAEVTRTFQKRL
Q9SYP1_ARATH   VKITEMPDWAQPAFKGMQQLNRVQSKVYDTALFKAENILLCAPTGAGKTNVAMLTILQQLEMNRNTDGTYNHG--DYKIVYVAPMKALVAEVVGNLSNRL
A9RTW1_PHYPA   VKISDMPDWAQPAFKGMKSLNRVQSKVYETALFTSENLLLCAPTGAGKTNVAMLTILHELGLRKQLDGTFDLS--SFKIVYVAPMKALVAEMVGNFSERL

                    710       720       730       740       750       760       770       780       790       800
                      |         |         |         |         |         |         |         |         |         |
Mipuc05g04870  APLGITVRELTGDTQLTKKELEETTMIVTTPEKWDVITRKGGEVSVASTLGLLIIDEVHLLNDERGPVIETLVARTHRQVETTQSMIRIVGLSATLPNPM
Mipur08g03240  EPLGIQVRELTGDTQLTKKEMEETHMIVTTPEKWDVITRKGGEVSVASSLRLLIIDEVHLLNDERGPVIETLVARTHRQVETTQSMIRIVGLSATLPNPA
Ol08g01930     APLDIVVAELTGDTQMSKRELEETQMIVTTPEKWDVITRKGGEVSVASTLRLLIIDEVHLLNDERGPVIETLVARTLRQVEQTQSMIRIVGLSATLPNPV
Ot08g01860     APLDIVVAELTGDTQMSKRELETTQMIVTTPEKWDVITRKGGEVSVASTLRLLIIDEVHLLNDERGPVIETLVARTLRQVEQTQSMIRIVGLSATLPNPL
Bathy01g04990  DELGMVCRELTGDTQLSKRELEETHVIVTTPEKWDVITRKGGEVSVASTLRLLIIDEVHLLNDERGPVIETLVARTRRQVEQTQSMIRIVGLSATLPNPR
Q9SYP1_ARATH   KDYGVIVRELSGDQSLTGREIEETQIIVTTPEKWDIITRKSGDRTYTQLVRLLIIDEIHLLHDNRGPVLESIVARTLRQIETTKENIRLVGLSATLPNYE
A9RTW1_PHYPA   EPYGVTVRELTGDATLSRGQIEETQIIVTTPEKWDIITRKSGDRTYTQMVKLLIIDEIHLLHDNRGPVLESIVARTVRQIETTQEMIRLVGLSATLPNYE

                    810       820       830       840       850       860       870       880       890       900
                      |         |         |         |         |         |         |         |         |         |
Mipuc05g04870  DVAKFLGVS-DAGLFVFDQSYRPIPLTQVFVGVTEGNAMKRLNLMAEIAYDKCAGALKSGKQAMVFVHSRKDTVKTARQLAELAAN--AEGGVELFGCAE
Mipur08g03240  DVAKFLGVS-DAGLFVFDQSFRPIPLTQMFVGVTEGNAMKRQMLMAQIAYDKCTAALRSGKQAMVFVHSRKDTVKTAKQLGEIAANDQTQGGLELF-APE
Ol08g01930     DVARFLGVNNDAGLFVFDQSYRPIPLTQKFIGVTEKNSMKRQTLMAQIAYNKACEALRNGKQAMVFVHSRKDTVKTAKQLAEFAAA---QDGMELF-SNN
Ot08g01860     DVARFLGVNNDAGLFVFDQSYRPIPLTQKFIGVTEKNAMKRQTLMTQIAYNKACEALKNGKQAMVFVHSRKDTVKTARQLAEFAAA---QGGLELF-SNE
Bathy01g04990  DVARFLGVTEGKGLFVFDQSYRPIPLTQVFIGVSETNAMKRQNVTIRIAFKKACEALRKGKQAMVFVHSRKDTVKTARQLAEIAGE---EGELELF-END
Q9SYP1_ARATH   DVALFLRVDLKKGLFKFDRSYRPVPLHQQYIGISVKKPLQRFQLMNDLCYQKVLAGAG-KHQVLIFVHSRKETSKTARAIRDTAMA---NDTLSRF-LKE
A9RTW1_PHYPA   DVALFLKVDEKKGLFYFDNSYRPCPLAQQYIGVTVRKPLQRFQLMNDICYEKVMEVAG-KHQVLIFVHSRKETAKTARAIRDAALA---NDTLGRF-LKE

                    910       920       930       940       950       960       970       980       990      1000
                      |         |         |         |         |         |         |         |         |         |
Mipuc05g04870  DDEGKKRFKTEIDRSRNNELKELVGKGFGCHNAGMLRSDRTLVEKLFAAGVVKVLVCTATLAWGVNLPAHTVVIKGTQLYDPQKGGFRDLGVLDVQQIFG
Mipur08g03240  NHPDFTTWKKEVERSRNNELKELFHRGFGCHNAGMLRSDRTLVERLFSAGVVKVLCCTATLAWGVNLPAHTVVIKGTTLYDPSKGGFRDLGVLDVQQIFG
Ol08g01930     QHERKAEFAQQVSRSRNNELKDLFLKGLGCHNAGMLRADRSLTEKLFAAGLIKVLVCTATLAWGVNLPAHIVVIKGTQLYDPQRGGFRNLGVLDVQQIFG
Ot08g01860     QNERKAEFAMQVSRSRNNEVKELFLKGLGCHNAGMLRADRTLTEKLFAAGLIKVLVCTATLAWGVNLPAHMVVIKGTQLYDPQRGGFRNLGVLDVQQIFG
Bathy01g04990  THEEKSIFAREVSRSRNQEMKELFFKGFGCHNAGMLRKDRTLVEQMFAAGVIKVLVCTATLAWGVNLPAHQVIIKGTQLYDASAGGFKDLGVLDVQQIFG
Q9SYP1_ARATH   DSVTRDVLHSHEDIVKNSDLKDILPYGFAIHHAGLSRGDREIVETLFSQGHVQVLVSTATLAWGVNLPAHTVIIKGTQVYNPEKGAWMELSPLDVMQMLG
A9RTW1_PHYPA   DGASREILQKENDVVKNNDLKNLLPYGFAIHHAGMGRADRTLVEDLFGDGHIQVLVSTATLAWGVNLPAHTVIIKGTQIYNPEKGAWTELSPLDVMQMLG
```

```
                  1010      1020      1030      1040      1050      1060      1070      1080      1090      1100
                     |         |         |         |         |         |         |         |         |         |
Mipuc05g04870    RAGRPGFDTSGEGVIVTEHKKLAHYLSLLTHSTPIESQFISCLADNLNAEIVLGTVTNVKEGAQWLGYSYLHTRMEKNPLAYGITWDDVKLDPGLGEHRR
Mipur08g03240    RAGRPGFDTSGEGVIITEHKKLAHYLALLTHSTPIESQFISCLADNLNAELVLGTVCSVKEGAQWLGYSYLHTRMEKNPLAYGLTWDDVNLDPGLVRHRR
Ol08g01930       RAGRPGFDTSGEGVIVTEHKNLAHYVSMLTHSTPIESQFVSNLADNLNAEVTLGTVTNVREGAQWLGYSYLHTRMEKNPLAYGLTWDDIRLDPGLLDHRR
Ot08g01860       RAGRPGFDTSGEGVIVTEHKNLAHYIAMLTHSTPIESQFISNLADNLNAEVTLGTVTNVREGAQWLGYSYLHTRMEKNPLAYGLTWDDVRLDPGLLDHRR
Bathy01g04990    RAGRPGFDTSGEGVIITEHKKLTKYVAMLTHSTPIESQFIECLADNLNAEIVLGTVTNVREGAQWLSYSYLHTRMEQNPLGYALTWDEVRLDPGLIEHRR
Q9SYP1_ARATH     RAGRPQYDQHGEGIIITGYSELQYYLSLMNEQLPIESQFISKLADQLNAEIVLGTVQNAREACHWLGYTYLYIRMVRNPTLYGLAPDALAKDVVLEERRA
A9RTW1_PHYPA     RAGRPQFDTYGEGIIITGHSELQYYLSLMNQQLPIESQYISKLADNLNAEIVLGSVQDAREACDWLGYTYLYIRMLKNPTLYGVSREALEADPSLEERRA

                  1110      1120      1130      1140      1150      1160      1170      1180      1190      1200
                  00 |         |         |         |         |         |         |         |         |         |
Mipuc05g04870    KLVKEAARTLDRAKMIRFDERSGQLYQTEAGRIASHFYIKQTSMEMFDEHLKRHMSVPEVFHMVSHAGEFENISPREDEMPELETLRRDKKNACPIEVKA
Mipur08g03240    KLVTEAARTLHRAKMVRFDEKSGFIYQTEAGRIASHFYIKQASMELFDEHLQRHMSMPEVFHMVAQATEFENIAPREDEMPELEALRRNRKGACPLEIKA
Ol08g01930       KLIKEAARVLDRAKMIRFDERSGQLYQTEAGRTASHFYIRVNSMEVFDGLMHRHMTLPDIFHMISHSSEFENIVPREDEIPELETLRRNRR-VVPIDIKA
Ot08g01860       KLIKEAARTLDRAKMIRFDERSGQLYQTEGGRTASHFYIRVSSMEVFDSLMHRHMTLPEVLHMISHSSEFENIVPREDEIPELETLRRDRR-IIPVEIKA
Bathy01g04990    NLIKTAARKLHKAKMIRFDEQSGQLYQTEAGRIASHFYIKVTSMELFEEMMNRHMSLPEVLHVISHSSEFENIAPREDEMPELEALRRNRRSACPIEIKG
Q9SYP1_ARATH     DLIHSAATILDKNNLVKYDRKSGYFQVTDLGRIASYYYITHGTIATYNEHLKPTMGDIDLYRLFSLSDEFKYVTVRQDEKMELAKLLDRVP----IPIKE
A9RTW1_PHYPA     DLVHSAAIVLDRNNLVKYDRKSGYFQVTDLGRIASYYYISHGSMATYNEHLKPTMGDIELCRLFSLSEEFKFVTVREEEKMELAKLLDRVP----IPVKE

                  1210      1220      1230      1240      1250      1260      1270      1280      1290      1300
                     |         |         |    00   |         |         |         |         |         |         |
Mipuc05g04870    TLADKAGKVNLLLQVYISRARMEAFSLIADSSYISQNASRICRALYELCLRRGWPSLAETLLTLLKTVDLRIWPHQHTLRQFETTLSPDTLYRLETRDAT
Mipur08g03240    TLADRAGKVNLLMQVYISRARMEAFSLVADSSYISQNASRICRALFELCLRRGWPSLAEELLTLSKAVDLRIWPHQHALRQFEQTLSPETLYKLEERQAT
Ol08g01930       SLTDKVGKVNLLLQVYISRASMQSFSLIADSMYISQNASRICRALFELCLRRGWPSLAEQLLTVSKSCDLRIWPHQHELRQFEKSLKPEVLFKLEEKKAT
Ot08g01860       SLTDRVGKVNLLLQVYISRANMQSFSLIADSMYISQNASRICRALFELCLRRGWPSLAEQLLTVSKACDLRIWPHQHELRQFEKTLKPEVLYKLEEKKAT
Bathy01g04990    DMSDKIAKVNLLLQVYVSRKRLESFSLVADSSYISQNASRICRALFELMLKRGWPSLAETLLTLSKAVDRRLWPHHSPLRQFENTLKPETIYKLEEKDAT
Q9SYP1_ARATH     TLEEPSAKINVLLQAYISQLKLEGLSLTSDMVYITQSAGRLVRALYEIVLKRGWAQLAEKALNLSKMVGKRMWSVQTPLRQFHG-LSNDILMQLEKKDLV
A9RTW1_PHYPA     SLEEPSAKINVLLQAYISQLKLEGLSLTSDMVFITQSAGRLMRALFEIVLKRGWAQLAEKALTLCKMVSRRMWSSQTPLRQFKG-IPNDILSKVEKKDLP

                  1310      1320      1330      1340      1350      1360      1370      1380      1390      1400
                     |         |         |         |         |         |         |         |         |         |
Mipuc05g04870    VERLWDMSPSEIGSLLRLNTDVGKKVKGCLEALPHLAMEASVQPITRSVLRVSVTLTPDFIWRDSQHGGIQRWLVWVEDPVNEHIYHTETFNLSKKQHKE
Mipur08g03240    VERLFDMSAQEIGSMLRLNTAVGQKVRGCLESLPHLTMEATVQPITRSVLRVTVALTPEFKWRDAVHGGLQRWLVWVEDPVNEHIYHNETFMLSKKLHGE
Ol08g01930       LDRLWDMSASEIGSMLRLNTQIGGQVKSCMRAMPHLNMTAVVQPITRSVLRVSVTLTPEFEWRDAVHGALQRWLIWVEDPVNEHIYHSETFNLSKKQSRD
Ot08g01860       LDRLWDMSGGEIGSMLRLNAQIGGQIKSCMRAMPHLNMTATVQPITRTVLRVSVTLIPEFEWRDQLHGALQRWLIWVEDPVNEHIYHSETFNLSKKQCRD
Bathy01g04990    VDRLIDVSAKEVGDLLRLNAVVGAQVKRCVEQLPHVNLEAVVRPITRSVLRVSATLTPEFMWRDEVHGQAQQWLIWVEDPVNEHIYHTETFTLSKKQYKE
Q9SYP1_ARATH     WERYYDLSAQELGELIRS-PKMGKPLHKFIHQFPKVTLSAHVQPITRTVLNVELTVTPDFLWDEKIHKYVEPFWIIVEDNDGEKILHHEYFLL-KKQYID
A9RTW1_PHYPA     WERYYDLSSQEIGELIRY-PKMGKSIHRYIHQFPKLELAAHVQPITRSVLKVDLTITPDFQWDEKYHGYVESFWVIVEDNDGENILHHEYFLL-KMQYVE
```

```
                   1410      1420      1430      1440      1450      1460      1470      1480      1490      1500
                      |         |         |         |         |         |         |         |         |         |
Mipuc05g04870   GKQHMAFTIPIFEPMPSQYFLRATSESWLGCETFLELRFDGLVLPQKHPPHTDLLDLTPLPRSALND--------EKYESLYAKKFTHFNAIQTQAFHTL
Mipur08g03240   GKQHLAFTIPIFEPVPPQYFLRATSESWLGCETFLELNFNELVLPDRGPAHTELLDLPPVPRQALYPPENPELGRKEFFDLYEGKFEFFNKVQTQAFNTL
Ol08g01930      GAQYLAFTIPIFEPVPPQYFLRAMSETWLGCESFVELNFQHLILPEEHPPHTELLDLDPLPRSALNN--------PVYESMYEGKFTHFNAIQTQAFHTL
Ot08g01860      GAQYLAFTIPIFEPVPPQYFLRAISENWLGCESFVELNFQHLILPEEHPPHTELLDLDPLPRSALKN--------PVFESMYEKKFTHFNAIQTQAFHTL
Bathy01g04990   GRMTLAFTIPIFDPRPPQYFLRATHLYWLGCESFLELDLEDIVLPTEPPPNTELLDLEPLPRSALNN--------PTYESLYEKKFTHFNAIQTQAFHTL
Q9SYP1_ARATH    EDHTLHFTVPIFEPLPPQYFVRVVSDKWLGSETVLPVSFRHLILPEKYPPPTELLDLQPLPVTALRN--------PNYEILYQ-DFKHFNPVQTQVFTVL
A9RTW1_PHYPA    EDHNLSFTVPIYEPLPPQYFVRVVSDRWLGSETVLPVSFRHLILPEKYPPPTELLDLQPLPVSALRN--------PSYEVLYQ-KFRHFNPIQTQVFPVL

                   1510      1520      1530      1540      1550      1560      1570      1580      1590      1600
                      |         |         |         |         |         |         |         |         |         |
Mipuc05g04870   FHTNVNVLLGAPTGSGKTISAELAMMRTFRDE-P-GGKVVYIAPLKALVRERIEDWRKHLCPVLGKRLVELTGDYTPDLRALLSADIIVATPEKWDGISR
Mipur08g03240   FHSESNVLLGAPTGSGKTISAELAMMAAFRDH-P-GGKIIYIAPLKALVRERIEDWKGKLCKVLNKKLVELTGDYTPDIRALQGADIIVCTPEKWDGISR
Ol08g01930      YHTDTNVLLGAPTGSGKTISAELSMMKVFRDS-P-GSKVVYIAPLKALVRERIKDWRKNLCPTLGLRMVELTGDYTPDLRALLQADIIVSTPEKWDGISR
Ot08g01860      YHTDTNVLLGAPTGSGKTISAELAMMKVFRDY-A-GSKVVYIAPLKALVRERIKDWRKNLCPTLGLRMVELTGDYTPDLRALLQADIIVSTPEKWDGISR
Bathy01g04990   YHTNHNVLLGAPTGSGKTISSELTILKMFRDE-PPGSKVVYIAPLKALVRERVDDWKKYFCPTVNKKMVELTGDYTPDLRALLRADIIVATPEKWDGISR
Q9SYP1_ARATH    YNTNDNVLVAAPTGSGKTICAEFAILRNHHEGPDATMRVVYIAPLEAIAKEQFRIWEGKFGKGLGLRVVELTGETALDLKLLEKGQIIISTPEKWDALSR
A9RTW1_PHYPA    YNTDDNVLVAAPTGSGKTICAEFAVLRMLQKG-EAGGRCVYIAPVEALAKERLRDWESKFGRTLGVRVVELTGETATDMKLLEKGQIIISTPERWDVLSR

                   1610      1620      1630      1640      1650      1660      1670      1680      1690      1700
                2     00        |         |         |         |         |         |         |         |         |
Mipuc05g04870   NWQSRAYVQKVSLVVIDEIHLLGADRGPILEVIVSRMRYISARTKQPVRIVGLSTALANARDLGDWLGIEDE-----GLFNFRPSVRPVPLECHIQGFPG
Mipur08g03240   QWQARSYVTKVSLVVIDEIHLLGADRGPILEVIVSRMRFISTRTERPVRIVGLSTALANANDLADWLGIEKQEGPKSGLFNFKPSVRPVPLECHIQGYPG
Ol08g01930      NWQSRAYVTKVALVVIDEIHLLASDRGPILEVIVSRMRYISARTGSNVRIVGLSTALANARDLGDWLGIDKE-----GLFNFRPSVRPVPLECHIQGFPG
Ot08g01860      NWQSRAYVKKVALVVIDEIHLLASDRGPILEVIVSRMRYISARTGSNVRIIGLSTALANARDLGDWLGIEEE-----GLFNFRPSVRPVPLECHIQGFPG
Bathy01g04990   NWQSRSYVSKVKLVIIDEIHLLGADRGPILEAIVSRMNYISARTKSKIRIVGLSTALANARDLGDWLGIENDK----GLFNFRPSVRPVPLECHIQGFPG
Q9SYP1_ARATH    RWKQRKYVQQVSLFIVDELHLIGGQHGPVLEVIVSRMRYISSQVINKIRIVALSTSLANAKDLGEWIGASSH-----GLFNFPPGVRPVPLEIHIQGVDI
A9RTW1_PHYPA    RWKQRKHVQQVSLFVVDELHLIGGEGGPVLEVIVSRMRYIGSQTENQIRIVALSTSLANAKDLGDWIGASSH-----GLFNFPPGVRPVPLEIHIQGVDI

                   1710      1720      1730      1740      1750      1760      1770      1780      1790      1800
                      |         |         |         |         |         |         |         |         |         |
Mipuc05g04870   KFYCPRMMTMNKPTYAAIRTHSP-EKPTLVFVSSRRQTRLTAMDLIAYAAAD-ERPEGFVHMSANELAGVVRRARDPALKHCLQFGIGIHHAGLSPEDRA
Mipur08g03240   KFYCPRMMTMNKPTYAAIRTHSP-EKPALVFVSSRRQTRLTAIDLIAYAAAD-ERPDTFVHMDPYEMEMHLAKVKSPELRHTLQFGVGLHHAGLAPEDRA
Ol08g01930      KFYCPRMMTMNKPTYAAIRTHSP-EKPTLVFVSSRRQTRLTALDLIAYAAAD-ERPDGFVHMSDDELTMHLSKVKDPALKHTLQFGIGLHHAGLTPEDRE
Ot08g01860      KFYCPRMMSMNKPTYAAIRTHSP-TKPALVFVSSRRQTRLTALDLIAYAAAD-ERPDGFVHMSNEELSIHLSKVKDPALKHTLQFGIGLHHAGLTPEDRE
Bathy01g04990   KFYCPRMLSMNKPTYAAIRTHSP-LKPALVFVSSRRQTRLTALDLIAYAAAD-ENPDAFVHCNSQELEQRIAKIQDPALKHTLQFGIGLHHAGLSPEDRG
Q9SYP1_ARATH    SSFEARMQAMTKPTYTAIVQHAKNKKPAIVFVPTRKHVRLTAVDLMAYSHMDNPQSPDFLLGKLEELDPFVEQIREETLKETLCHGIGYLHEGLSSLDQE
A9RTW1_PHYPA    ANFEARMQAMTKPTYTAIVHHVKKQEPALIFVPTRKHARLTALDLVTYATVNGNGKSPFLHCAEADLAPFLSKVKDEALIHALLQGIGYLHEGLSAIEQE
```

```
                   1810      1820      1830      1840      1850      1860      1870      1880      1890      1900
                      |         |         |         |         |         |         |         |         |         |
Mipuc05g04870   ICEELFAECKIQVLVCTSTLAWGVNLPAHLCIIKGTEFYDGKSRRYVDFPITDVLQMMGRAGRPQFDKSGCCVIMVHEPKKAFYKKFLYEPFPVESSLAD
Mipur08g03240   LCEELFLKCKIQVLVCTSTLAWGVNLPAHLVVIKGTEFYDGKTRRYVDFPITDVLQMMGRAGRPQFDTSAVAVIMVHEPKKAFYKKFLYEPFPVESSLAD
Ol08g01930      LCEELFAQCKIQVLVTTSTLAWGVNLPAHLVVIKGTEYFDGKTKRYQDFPITDVLQMMGRAGRPQFDKSGCCVILVHEPKKTFYKKFLYEPFPVESSLAE
Ot08g01860      LCEELFAQCKIQVLVTTSTLAWGVNLPAHLVVIKGTEYFDGKTKRYQDFPITDVLQMMGRAGRPQFDKSGCCVILVHEPKKTFYKKFLYEPFPVESSLAE
Bathy01g04990   VAEQLFAECKIQVLVSTSTLAWGVNLPAHLVVIKGTEFYDGKTKRYQDFPITDVLQMMGRAGRPQFDKSGCCVVLVHEPKKNFYKKFLYEPFPVESSFNE
Q9SYP1_ARATH    IVTQLFEAGRIQVCVMSSSLCWGTPLTAHLVVVMGTQYYDGRENSHSDYPVPDLLQMMGRASRPLLDNAGKCVIFCHAPRKEYYKKFLYEAFPVESQLQH
A9RTW1_PHYPA    VVTSLLTAEAIQVCVATSSMCWGMTLSAHLVVVMGTQFYDGRENAHTDYPITDLLQMMGRASRPQVDTSGKCVILCHAPRKEYYKKFLYEPFPVESHLDH

                   1910      1920      1930      1940      1950      1960      1970      1980      1990      2000
                      |         |         |         |         |         |         |         |         |         |
Mipuc05g04870   NLPDHFNAEVVAGTIRSKQDAVDYLTWTYFFRRLVQNPSYYDCEGVEHAELNAFLSRLVENALVMLEDARCVEIGE-DDSVAPLLLGRIASYYYLQHPSV
Mipur08g03240   QLPDHFNAEVVAGTIRSKQDAVDYLTWTYFFRRLVKNPSYYDLESVEHDALNAFLSRLVENALAQLEDAQCLTIGE-DDSLEPATMGRIASFYYLQHPSV
Ol08g01930      NLCDHFNAEIVSGTIKTKQDAVDYLTWTYFFRRLLKNPTYYNLDTIQTDNLNEYLSDLVENALELLEDARCIAIDEEDDGLEPLMLGRVASYYYLQYPSV
Ot08g01860      NLCDHFNAEIVSGTIKTKQDAVDYLTWTYFFRRLLKNPTYYNLDTIEADKMNEYMSDLVEGALELLEDARCIAIDDDDDSLEPLMLGRVASYYYLQYPSV
Bathy01g04990   CLEDHFNAEVVGGAIKSKQDAVDYLTWTYFFRRAMKNPTYYNLEDTNHETVNSYLSEMVENTMETLASAKCLAINDEDDSIKPLMLGRIASFYYLNFKTM
Q9SYP1_ARATH    FLHDNFNAEVVAGVIENKQDAVDYLTWTFMYRRLPQNPNYYNLQGVSHRHLSDHLSELVENTLSDLEASKCIEVED-EMELSPLNLGMIASYYYISYTTI
A9RTW1_PHYPA    YLHDHLNAEVVVRTIENKQDAVDYLTWTFMYRRLTQNPNYYNLQGVSHRHLSDHLSELVESTLSDLESSKCVAIED-DMDLSPLNLGMIAAYYYISYTTI

                   2010      2020      2030      2040      2050      2060      2070      2080      2090      2100
                      |         |         |         |         |         |         |         |         |         |
Mipuc05g04870   ALFASSLSHANTVEQLLKTLCGVAEYDELPVRHNEDKVNAELAIRVKEAGGFAVDARLADDPHTKANLLFQAHFLRVPLPMSDYVTDTKSVLDQAIRIIQ
Mipur08g03240   ALFASSLGPDTSLEQLLGILCGVAEYDELPVRHNEDKVNAELARQVEDAGGFKVDARLADDPHTKANLLFQAHFLRLQLPMSDYVTDAKGVLDQAVRILQ
Ol08g01930      ALFASNIKANSSLESLLETLCGVAEYDELPVRHNEDKLNTELAEVVADAGGFQVDIRLAEDPHVKTSLLFQCHFLRLPLPLSDYYTDTKSVLDQAIRILQ
Ot08g01860      ALFASNIKANSSLEDLLETLCGVAEYDELPVRHNEDRHNTELAQVVADAGGFQVDVRLAEDPHVKTSLLFQCHFLRLPLPVSDYYTDTKSVLDQAIRILQ
Bathy01g04990   AVFSKRLKKSNTLEDVLTTLCDVAEYDEIPVRHNEDKLNADLAINVLKAGGYQVDRRAYDDPHVKASLLFQAHFLRLPLPMSDYHTDTKSVLDQSQRILQ
Q9SYP1_ARATH    ERFSSLLSSKTKMKGLLEILTSASEYDMIPIRPGEE----DTVRRLINHQRFSFENPKCTDPHVKANALLQAHFSRQNIGG-NLAMDQRDVLLSATRLLQ
A9RTW1_PHYPA    ELFSSSLTAKTKLKGLLEILSNASEYTRLPMRPGED----ELIRKLVMHQRFSMDKPKFTDPHVKANALLQAHFARHSVSG-NLALDQRDILIDASRLIQ

                   2110      2120      2130      2140      2150      2160      2170      2180      2190      2200
                      |         |         |         |         |         |         |         |         |         |
Mipuc05g04870   AIIDVASDAGWLHTALNAMRLMQMVMQGRFLTDSPLTTLPHVDAEVAGKLRRG-GVKSLPQFVTRAIKDRAGAKKALCAAGLSGRTAEETTNVAARYPSV
Mipur08g03240   AIIDVCAESGWLATCLHAMNLMQMVMQGRFITDPSCMSVPGVDEQKAASLSGS-GYEALPQLVDACVNKEAAARKALTNAGLKQRQVDEAVNHCQRMPLI
Ol08g01930      AMIDVTSDAGWLHTALSTMNLMQMIMQGRMITDSSLLTLPHIERRHLRNLEKH-GLSILPQLMDLCSSNKQQARRVLSECGINGRKIDQVVDLCLRLPVI
Ot08g01860      AMIDVTSDAGWLRTAMNTMNLMQMIMQGRMISESSLLMLPHIQPDHLKGLERC-GVSGLSQLIDLSTKNIQKARKVLSDCGITGRKMEKIVDLCVRLPVL
Bathy01g04990   AMTEIVSEAGWLSTALSIMNLTQMIIQGLSINDNSLLLLPKIADEEVSKLERFSKITCLPQLVFLAIHRKSAFFEAMSKAGLSKSKSEAIYNVCANLPLL
Q9SYP1_ARATH    AMVDVISSNGWLNLALLAMEVSQMVTQGMWERDSMLLQLPHFTKDLAKRCQEN-PGKNIETVFDLVEMEDEERQELLKMS---DAQLLDIARFCNRFPNI
A9RTW1_PHYPA    AMVDVISSSGWLHPALAAMELSQMVTQGLWERDSYLLQLPYFTKDLAKKCADN-PDKPIQTVFDLVEMEDDERRELLQMS---DAQLMEIARVCNRFPNI
```

```
                       2210      2220      2230      2240      2250      2260      2270      2280      2290      2300
                         |         |         |         |         |         |         |         |         |         |
Mipuc05g04870   MMRASSVKTSRASAGGGKADEGVVEVHLKRLHARGGKDGGNGGGGRSSAPRAVCPLFPKLKEEGWWLVLGD-RISGELLALRRVGFGGAASAKLTYAAPD
Mipur08g03240   DIDVKLSK-----------DGTEVEVNLRRTSKSAG--GGGKGGGRGSAPRAILPRYPKVKEEGWWLILGD-RNNRELLSLKRVGFGQSARAKLAVDRSA
Ol08g01930      DAKATTETTKGIN------GEKTVHVKLRRIGKKCG--------SK--APTSYTPRFPKIKEEGWWIVVGD-TANDELLALRRISFGDAANVKLKCPSGS
Ot08g01860      HVEASIETVKGG-------GDTTVHVQIRRIGKKCG--------SK--APTSYTPRFPKIKEEGWWVIVGD-TANNELLALRRISFGDRADVKLKCPPSA
Bathy01g04990   DMKATLVEDKVNSSDG--RRNVSVKVSLKRSGKKSG---------RKTAPRAYAPRFPKQKDEGWWIVLGEKRRTGELVAMRRAQYADTFDAVLKIDNFP
Q9SYP1_ARATH    DLTYEIVGSEEVNP-G---KEVTLQVMLER--DMEG---------RTEVGPVDSLRYPKTKEEGWWLVVGD-TKTNQLLAIKRVSLQRKVKVKLDFTAPS
A9RTW1_PHYPA    DLAHEVLDNDDISP-G---DTVTLQVTLER--EMEG---------RQELSPVDAPRFPKPKEEGWWLVVCE-PKSNQLLAIKRVSLQRRSKVKLDFTAPN

                       2310      2320      2330      2340      2350      2360      2370      2380      2390      2400
                         |         |         |         |         |         |         |         |         |         |
Mipuc05g04870   APIGGGRGPELDLVVHLVSDCYVGMDQDLGVSEGLPAS--IDAEEDGDSSDDDGFWLPAAAVAERMKA-ASETARRLASESESESESEDDEAFWEDETPT
Mipur08g03240   NAV-----FEPDLHVYLISDCYVGLDQEVEVARGAGAVGAEDAGDTGDTDDEQGFWLSPEQVAARLAARATERATEDTDSDEDFWEMPAAAAPAGERYSA
Ol08g01930      SSRAR-----PDLVVFLMSDSYIGLDQEVKIDSNTMVD---EDSSDEFAEDDDTFWALP-----------------PD-STEPFWLGEG------------
Ot08g01860      SPRPRR----QTLAVYVVSDSYIGLDQEILINADDFVE---VSD-DEVDDNADTFWLLP-----------------PT-QTEPFWLGEAD-----------
Bathy01g04990   RGMS-----VTDITVFIMSDTYIGLDQEVLVSNTDDKR----FLSSAGVADRHRFFEERE------------S---DSEAEGNFWQDEDELS--------
Q9SYP1_ARATH    EPGE------KSYTLYFMCDSYLGCDQEYSFSVDVKG------SGAGDRMEE------------------------------------------------
A9RTW1_PHYPA    EVGR------KTYTLFFMCDAYLGCDQENEFTIDVKEG--VDAEDDGNAMEE------------------------------------------------

                       2410      2420      2430
                         |         |         |
Mipuc05g04870   EEVAAAAVDQDAFFWEGEGAYLAAGGDGEKKT
Mipur08g03240   VMAAKMPVEEDPFFWENEREYLDAK-------
Ol08g01930      -----------------ENTLLT---------
Ot08g01860      -----------------ENSLLT---------
Bathy01g04990   -----------------DEDIPDF--------
Q9SYP1_ARATH    --------------------------------
A9RTW1_PHYPA    --------------------------------
```

e) UAA1

```
                         10        20        30        40        50        60        70        80        90       100
                         |         |         |         |         |         |         |         |         |         |
Mipuc16g00610     MKTRARDAGTRGGD------------RDADARGRGAPRGRRAGA---------GVFSTR----ARERSGAR----AAEARTDATSTR--------AAV
Mipur10g04930     MSDRPAYHLVPGRED---------PPTNHDPPPMNPPLGHRSSD---------HVGSVR----LLEALGAPSRRDDGEPEKGASILS--------SSG
Ol16g00570        MGVAASARASRPSDFVDADDDDAWRRADAGARARRGRRETSAGS----FSAIPSVSSFGGGLMNYERQGARVTRRSPSNEGGDVEQGLADWGRDDASI
Ot17g00460        MDDAASDGRGR-------TDDARWTSASAGARDRARGRETRSGADGQRFETIPSVSSFGGGLMNYEVFSRRSSANEMSSEP-DVERG----GVEPSPV
Bathy06g01490     MGLFDGFSSSSSKPSSS-------LNASSSSKNDAFPMNSILSDD---VETSLVVNASNNN--KHNNRGEEEETITMMDNNNNLSVN-------EMPM
                  :                          . ..           .           * :        :  .          .                .
```

```
                          110       120       130       140       150       160       170       180       190       200
                           |         |         |         |         |         |         |         |         |         |
Mipuc16g00610     PRRAERRR---DPAKLWGVPLRMSD-KDLVYFLVSGAILSYLGFTATQEGVFR-----GADFHYGGMVTLFTTLVYCAAAAIERKKSNDAV--RKGSWRN
Mipur10g04930     AGGTNRNA---EPAILWGIPLRFADERRLAYFLGGGAVACYLAFTATQEGVFA-----SMGGAHGGMVTLVTTAVYCCLAFGERVRSGETH--RKGTWRD
Ol16g00570        SPGATKRASGATPARVWGRELRLSE-GALLATLSAGALFAYVAFTMTQEGVFRR---ATKEFKYGGAVSLCTCLVYCALAQCERVSNGDHG-ARRGNLKD
Ot17g00460        SPGTLKRLNADAPVKVWGREVRMSE-LGLLMTLSGGALFAYVAFTMTQEGVFRR---ATKDFKYGGVVSLCTSLVYCGLAQCERASNGDAPWSRRGDIRD
Bathy06g01490     SPQPSKEK---DPIKLWGVKLLFSE-RSIFWLLASGALFAYLGFTTVQEYVFRNHAKEAIGFRFGGVVTLVTSFTYALLGFLERLANDE----RRAKLKD
                  .  . :.     *  :**  : :::   :   * .**: .*:.** .** **           .** *:* *  .*.  .  ** ..:       .  ::

                          210       220       230       240       250       260       270       280       290       300
                           |         |         |         |         |         |         |         |         |         |
Mipuc16g00610     YLILAAITTGSMWITNAAMQYLNYTTRIVAKSSKVIPTMILGTFMQGRTYGRDEYGCAILLVCGIALFTMGDVDAAPSFDPTGVVMIFVALFLDSAAGNF
Mipur10g04930     YLILAAMTSGGMYATNAALAYLNYTTRIVAKSSKVIPTMLLGTVMQGRRYSASEYLAAGMLVLGIALFTMGDVDTLPSFEVKGIVLIAVALCLDSAAGNF
Ol16g00570        YAMLSVMTSGSMYLTNAALSYINYTTRIVAKCSKVIPVMIVGTFMHGRRYGSEDYAMCVLLVIGITMFTMGDVDSFPNFNYRGVTYITIALFTESTAGNF
Ot17g00460        YALLSVMTSGSMYLTNAALSYINYTTRIVAKCSKVIPVMIVGTLMQGRRYGVEDYGMCILLVVGITLFTMGDVDSFPNFDYRGVAYITIALFLESTAGNF
Bathy06g01490     YVLLATMTSGGMYLTNFSLSYINYTTRIVAKCSKVIPTMVMGALMQGRRYEKKDYFAAMTLVCGVCLFALGDRASLPQFQPKGVVMIVCALFIEAAAGNF
                  * :*:.:*:*.*: ** :: *:*********.*****.*::*:.*:** *  .:*  .  ** *: :*::**  : *.*:  *:. *  **  :::****

                          310       320       330       340       350       360       370       380       390       400
                           |         |         |         |         |         |         |         |         |         |
Mipuc16g00610     EERRFFNIPDPVTHAEVVFHSNLIGIGFSAIAMLFSGELLPAFAHAFANATVLPAICVAALFGYLSVSFIMLLIRHYGASNTEIVKSTRKMISIGASMLL
Mipur10g04930     EERRFFNVPDPVHHAEVVYHANLIGMGLTCVGMWLSGELWVSVAFVASNVTSTPLMAVAAAFGYLSVSFILLLIRHYGAANTEVVKSMRKMVSIALSMTL
Ol16g00570        EERRFFNIPQPISHCEVVFYVNAIGSAWIALGLFASGELFTAVAHLLGEPDVLAAICVAAAFGYISVTCILLCLRHYGATNTEVIKAVRKMLSLALSLVV
Ot17g00460        EERRFFNLPKPISHCEVVFYVNAIGSVWIALGLFASGELFVSLAHISREPTMLVAICLAAAFGYISVTCILLCLRHYGATNTEVIKAVRKMLSLALSLLV
Bathy06g01490     EEKRLFNVSLPASHAEVVMHANIFGLLMTTFGMTLNGEIWPIVAYMNSHRECVFRAMIAASFGYMSVSFILLSIRQYGATNTEIIKALRKMLSIALSLLL
                  **:*:**:. *  *.*** : * :*     ..:  .**:   .*.   .       :** ***:**: *:* :*:***:***::*: ***:*:. *: :

                          410       420       430       440
                           |         |         |         |
Mipuc16g00610     YPAPWNWKYGAGLACTGAALYGLYLLKRRKLAASG--GSIEATK
Mipur10g04930     YPKPWDWKYGAGAASTVVGLYALYAIKRRKYLAAGPGGSMEAAK
Ol16g00570        YPKPMGWKYVVGTGATATALFGLYKIKMRRLRLAG--GSMEAAK
Ot17g00460        YPKPMGWKYIVGTTATCVALFGLYKIKMRRLRAAG--FSMEAAK
Bathy06g01490     YPKPMGWRYVTGTCVTAFGILTLYAIKKRRYVKAA--GNAEAFK
                  ** * .*:* .*   *  .:  ** :* *:   :.   . ** *
```

Figure S9. Alignment of all intron encoded proteins (IEP) within IE-B sequences. The divergent group of IEPs (blue) and the 'OTU-like cysteine protease domain' (yellow) have been highlighted.

```
                      10        20        30        40        50        60        70        80        90       100
                       |         |         |         |         |         |         |         |         |         |
Mipuc07g03770  ----------------------------------------------------------------------------------------------------
Mipuc09g04130  ----------------------------------------------------------------------------------------------------
Mipuc01g03710  ----------------------------------------------------------------------------------------------------
Mipuc03g05030  MTPERVVTWGVVAMSVAVLRIVTEAFMAVMSGCGDVEGSSVHATLVVSPPITSVSTAAPLRPPADSPPARLAAGMRTRAVRRKWRVCVFQERRKALMMAM
Mipuc07g01720  -------------MSVAVLRIVTEAFMAVMSGCGDVEGSSVHATLVVSPPITSVSTAAPLRPPADSPPARLAAGMRTRAVRRKWRVCVFQERWKALMMAM
Mipuc03g01960  MTPERVVTWGVVAMSVAVLRIVTEAFMAVMSGCGDVEGSSVHATLVVSPPITSVS---------------------------------------------
Mipuc01g06560  ----------------------------------------------------------------------------------------------------
Mipuc03g01120  ----------------------------------------------------------------------------------------------------

Prim.cons.     MTPERVVTWGVVAMSVAVLRIVTEAFMAVMSGCGDVEGSSVHATLVVSPPITSVSTAAPLRPPADSPPARLAAGMRTRAVRRKWRVCVFQER2KALMMAM

                      110       120       130       140       150       160       170       180       190       200
                       |         |         |         |         |         |         |         |         |         |
Mipuc07g03770  ----------------------------------------------------------------------------------------------------
Mipuc09g04130  ----------------------------------------------------------------------------------------------------
Mipuc01g03710  ----------------------------------------------------------------------------------------------------
Mipuc03g05030  MMTMMTAASVATPTPDDADAKATPVKASTATDET-----------------------------------------------VFVATPPDDADTAPSEASSP
Mipuc07g01720  MMTMMTAASVATPTPDDADAKATPVKASTATDETHGETPSSVLVSPAVESTLVPAAAESTVEAAAAITASSEASLPTSEPQSSVAHSAATATAPSEASSP
Mipuc03g01960  -----TAASVATPTPDDADAKATPVKASTATDET-----------------------------------------------VFVATPPDDADTAPSEASSP
Mipuc01g06560  ------------------------------------------------------------------------------------MYGEAQPPLASDDE
Mipuc03g01120  ------------------------------------------------------------------------------------MYGEAQPPLASDDE

Prim.cons.     MMTMMTAASVATPTPDDADAKATPVKASTATDETHGETPSSVLVSPAVESTLVPAAAESTVEAAAAITASSEASLPTSEPQVFVAT2222ATAPSEASSP

                      210       220       230       240       250       260       270       280       290       300
                       |         |         |         |         |         |         |         |         |         |
Mipuc07g03770  -------------MAAARAAGRLSRRLGAA-----------------LRDTAMNAARDFTRMGSSRAQKATAVKP-------------------------
Mipuc09g04130  -------------MAAARAAGRLSRRLGAA-----------------LRDTAMNAARDFTRMGSSRAQKATAVKP-------------------------
Mipuc01g03710  -------------MAAARAAGRLSRRLGAAT---------------IALRDTASNAARAFARKSSSRAQETTGVKP-------------------------
Mipuc03g05030  EPEPQPESSVAPSAAAASAAAAMTAPSEASSPPPEPQPESSAPPGAATLVPASNLNRCLRGGASSTGAKTITSSPNADETSTGPAFFPRSFMKKFRNNIL
Mipuc07g01720  EPEPQPESSVAPSAAAASAAAAMTAPSEASSPPPEPQPESSAPPGAATLVTASNLNRCLRGGGSSTGAKTITSSPNADETSTGPAFFPRSFMKKFRNNIL
Mipuc03g01960  EPEPQPESSVAPSAAAASAAAAMTAPSEASSPPPEPQPESSAPPGAATLVTASNLNRCLRGGGSSTGAKTITSSPNADETSTGPAFFPRSFMKKLRNNIL
Mipuc01g06560  LEDDPFVFDLDDEKLAEEYAAALHVPFVVIMSG---------------HNRRCCLAAEIGDSCAPRLDDIRRVTP--------------------LED--
Mipuc03g01120  LEDDPFVFDLDDEKLAEEYAAALHVPFVVIMSG---------------HNRRCCLAAEIGDSCAPRLDDIRRVTP--------------------LED--
                              *    *. :     .                          :    :.    .     .*

Prim.cons.     EPEPQPESSVAPS2AAA2AAAAL22P22A2SPPPEPQPESSAPPGAA222TASNLARC2R2GGSSR22KT2TV2PNADETSTGPAFFPRSFMKKFRNNIL

                      310       320       330       340       350       360       370       380       390       400
                       |         |         |         |         |         |         |         |         |         |
Mipuc07g03770  --------------------------------SPCILRAPKRTPTSTS----------------------------------------------------
Mipuc09g04130  --------------------------------SPCILRAPKRTPTSTS----------------------------------------------------
Mipuc01g03710  --------------------------------SRYDFRASTRTQTSTS----------------------------------------------------
Mipuc03g05030  GFELRSVGEPIQAAIGRFLTASAMKILNTRGDGNCMFRALLECMTGNSDKHVEFRLQVVNLMRSDPNSYRAEAEADQVDDAP------HDRPFDFDSYCD
Mipuc07g01720  GFELRSVGEPIQAAIGRFLTASAMKILNTRGDGNCMFRALLECMTGNSDKHVEFRLQVVDLMRSDPNSYRAEAEADQVDDAP------HDRPFDFDSYCD
Mipuc03g01960  GFELRSGGEPIQAAIGRFLTASAMKILNTRGDGNCMFRALLECMTGNSDKHVEFRLQVVGLMRDDPDSYRAEAEADQVDDAPHDDDAPHDRPFDFDSYCD
Mipuc01g06560  --------------------------------DSRAARLDEKYPQSQD----Q---------------------------D-------------------
Mipuc03g01120  --------------------------------DSRAARLDEKYPQSQD----Q---------------------------D-------------------
                                                .    *    .   .  .

Prim.cons.     GFELRSVGEPIQAAIGRFLTASAMKILNTRGD2NCMFRALL22PTS2SDKHVEFRLQVV3LMRSDPNSYRAEAEADQVDDAPHDDDAPHDRPFDFDSYCD

                      410       420       430       440       450       460       470       480       490       500
                       |         |         |         |         |         |         |         |         |         |
Mipuc07g03770  ------------------------------------------------------------------------------------ATPAPTLAPTPAP
Mipuc09g04130  ------------------------------------------------------------------------------------ATPAPTLAPTPAP
Mipuc01g03710  ------------------------------------------------------------------------------------ATPAATPAPTPAP
Mipuc03g05030  NMKRVGIFGGTPELHAASVLLKVDIMVIDVRYGRLVLDTGGDQPQERGTIAVAFDSDGKHYNAIVHLELSRRGFPDDTEASAATFSVNTTKASFADATPT
Mipuc07g01720  NMKRVGIFGGTPELHAASVLLKVDIMVIDVRYGRLVLDTGGDQPQERGTIAVAFDSDGKHYNAIVHLELSRRGFPDDTEASAATFSVNTTKASFADATPT
Mipuc03g01960  NMERDGIFGGTPELHAASVLLKVDIMVIDVSYGRLVLDTGGDQPQERGTIAVAFDSDGKHYNAIVHLELSRRGFPDDTEASAATFSVNTTKASFADATPT
Mipuc01g06560  ------------------------------------------------------------------------------------VTSRGSFSYTNAD
Mipuc03g01120  ------------------------------------------------------------------------------------VTSRGSFSYTNAD
                                                                                                    *.   :  :  :  .

Prim.cons.     NMKRVGIFGGTPELHAASVLLKVDIMVIDVRYGRLVLDTGGDQPQERGTIAVAFDSDGKHYNAIVHLELSRRGFPDDTEASAATFSV2T22ASFA2T2A2

                      510       520       530       540       550       560       570       580       590       600
                       |         |         |         |         |         |         |         |         |         |
Mipuc07g03770  TPAPTPRYILRASTRTQTSTSATP---AATPAATP-----------AATRAVAQQP-VSSRTRSGGRGGGG------------------GAGFDADPVE
Mipuc09g04130  TPAPTPRYILRASTRTQTSTSATP---AATPAATP-----------AATRAVAQQP-VSSRTRSGGRGGGG------------------GAGFDADPAE
Mipuc01g03710  TPAPTPRYILRASTRTQTSTSATP---AATPAATPA---------AAATRAVAQQP-VSSRTRSGGRSRGG------------------GDGFDADPVE
Mipuc03g05030  SGVSTAASVVKTRARAQISTSFSEEDEVTSPGADPRD------TEFTPPRSSSDAPNTRARARATGRSRDAPTATSRDARAAARSKRNAKHADDDVDPES
Mipuc07g01720  SGVSTAASVVKTRARAQISTSFSEEDEVTSPGADPRD------TEFTPPRSSSDAPNTRARARATGRSRDAPTATSRDARAAARSKRNAKHADDDVDPES
Mipuc03g01960  SGVSTAASVVKTRARAQISTSFSEEDEVTSPGADPRD------TEFTPPRSSSDAPNTRARARATGRSRDAPTATSRDARAAARSKRNAKHADDDVDPES
Mipuc01g06560  MLQMGFKKVKRPPEKDLFPPAMPDCLPPATPDEEDDDRMSLSYGMDVDDIPPVDSAGVATTARGKRRREDAP------------------SPGQRSSEAQ
Mipuc03g01120  MLQMGFKKVKRPPEKDLFPPAMPDCLPPATPDEEDDDRMSLSYGMDVDDIPPVDSAGVATTARGKRRREDAP------------------SPGQRSSEAQ
                        : :.  :    ..: .     ::*                   .    .   :  .  .  :  :*.  *   ..                  .    .   .

Prim.cons.     2222T222V2R222R2Q2STS222EDE2ATP2A2PRDRMSLSYTEF222R222D2PNV22RAR22GRSRDAPTATSRDARAAARSKRNAK2AG2D2DP22

                      610       620       630       640       650       660       670       680       690       700
                       |         |         |         |         |         |         |         |         |         |
Mipuc07g03770  YMTPAFNAALTRLR-------------------DGDVGRDDSAPPPPAKRRLAVESLSE-----LPPLPRRSS--------------------------
Mipuc09g04130  FMTLACNAVLSRLR-------------------DDGVGRDDSAPPPRAMRRLAVESTSE-----LPPPSRRSS--------------------------
Mipuc01g03710  YMTPAFNAALTRLR-------------------DGDVGRDDSAPPPPAKRRLAVESPSE-----LPPLPRRSS--------------------------
Mipuc03g05030  ESTAAASKHFPRATRPNLSAHGSKAAKRIKTSPSTSAARKQAAAPMPSRSRAQQATAAKPSRYNLRPPKRTPTSTSATPAATPAATPAATPAAT------
Mipuc07g01720  ESTAAASKHFPRATRPNLSAHGSKAAKRIKTSPSTSAARTQAAAPTPSRSRAQQATAAKPSRYNLRPPKRTPTSTSATPAATPAATPAATPAATPAATPA
Mipuc03g01960  ESTAAASKHFPRATRPNLSAHGSKAAKRIKTSPSTSAARTQAAAPTPSRSRAQQATAAKPSRYNLRPPKRTPTSTSATPAATPAATPAATPAATPAATPA
Mipuc01g06560  RLDAPAVKPVTRPSR-----------------TTKPIERFGFDDHATGASRAIPASAGR------VPGTSAPG---------------------------
Mipuc03g01120  RLDAPAVKPVTRPSR-----------------TTKPIERFGFDDHATGASRAIPASAGR------VPGTSAPG---------------------------
                    .      ..*                           *          .    *     :  ..          *      .

Prim.cons.     E2TAAA2KH2TR22RPNLSAHGSKAAKRIKTSP2TS22RD22A2PPP2RSRA22ASA22PSRYNL2PPKR2P2STSATPAATPAATPAATPAATPA
```

```
                  710       720       730       740       750       760       770       780       790       800
                    |         |         |         |         |         |         |         |         |         |
Mipuc07g03770  --------------------------------------------PRLQPSALVVTPAAPMTSAT----PRP---SVARTPSAAR----KAVTRGAHSVVQ
Mipuc09g04130  --------------------------------------------PPLQPSALVVTPAAPMTSAT----PRP---SVARTPSAAR----KAVTRGAHSVVQ
Mipuc01g03710  --------------------------------------------PRLQPSALVVTPAAPMTSAT----PRP---CAASTPSAAR----KPATRGAHSVVQ
Mipuc03g05030  ------------------PLQSVSSRTSRGRDRGGGDGFDANPPRLQSSALVATSAAPTPSAPRT-KPKSKAKPAARTKSKSKPYKGKAVTRGAHSVVQ
Mipuc07g01720  ATPAATPAATPVATPLQSVSSRTSRTRWRGRDRGGGDGFDANPPRLQSSALVATSAAPTPSAPRT-KPKSKAKPAARTKSKSKPYKGKAVTRGAHSVVQ
Mipuc03g01960  ATPAATPAATPAATPAATPLQSVSSRTSRGRDRGGGDGFDANPPRLQSSALVATSAAPTPSAPRT-KPKSKAKPAARTKSKSKPYKGKAVTRGAHLVVQ
Mipuc01g06560  ---------------------------------G----LAASAPRGRATPAPRGRATPAPRRVPDASAPRGRAAPAPESESVED-----EEAA-RFNAGI
Mipuc03g01120  ---------------------------------G----LAASAPRGRATPAPRGRATPAPRRVPDASAPRGRAAPAPESESVED-----EEAA-RFNAGI
                                                *  :.:.    *:*   ..    *:     .. : *            :   .  .

Prim.cons.     ATPAATPAATPAATP2ATPLQSVSSRTSRGRDRGGGDGFDANPPRLQ2SALV2T2AAP22SAPRTSKPR2KAKPAART2S222PYKGKAVTRGAHSVVQ

                  810       820       830       840       850       860       870       880       890       900
                    |         |         |         |         |         |         |         |         |         |
Mipuc07g03770  DMHSVETRNYDAGKVARQKRKSPDERRRDDAAAASRAANAARDAEEQEAFKLRYETIDELR-AAFEADPLSDDECVRDIKAEHDAMVAAAKKRAADEAK
Mipuc09g04130  DMHSVETRNYEAGKVARQQRKSLDERRRDDAAAEETRAANAARDAAEREAFKLRYETIDELRAAAVKAVQMSDDECVRDIKAEHDAMVAAAKKRAADEAK
Mipuc01g03710  DMHSVETRNYEAGKVARQKRKSPDERRRDDAAAEDARAANAARDAEEREALKLRYETIDKMR-AAVKADLMSEDACVKVIKAEHDAMVAAAKKRAADEAE
Mipuc03g05030  DMHSVETRNNEAGKVARQQRKSPDERRRDDAAAEEARAANAARDAEEREAFKLRYETIDELR-AAFEADPLSDDECVRDIKAEHDAMVAAAKKRAADEAK
Mipuc07g01720  DMHSVETRNYEAGKVARQQRKSPDERSRDDAAAEEARAANAARDAEEREAFKLRYETIDELR-AAFEADPLSDDECVRDIKAEHDAMVAAAKKRAADEAK
Mipuc03g01960  DMHSVETRNYEAGKVARQQRKSPDERRRDDAAAEEARAANAARDAAEREALKLRYETIDKMRAAAVKAVQMSEDACVKVIKAEHDAMVTAAKKRAADEAK
Mipuc01g06560  ICRAVERRNHDDGQRVKEQRTPVDVKHRAEEALELAAVVEAARVVKENADFEAVRKATLEER-AEFDLNPPSEAAYLKRVKQEHKKECDDARVRAEAGG-
Mipuc03g01120  ICRAVERRNHDDGQRVKEQRTPVDVKRRAEEALALAAVDEAARVVKENADFEAVRKATLEER-AEFDLNPPSEAAYLKRVKQEHKKECDDARVRAEAGG-
                 ::** ** : *: .:::*.. * : * : *    : . :*** . *.  ::    ::  : * * ..     *:    :: :* **.     *: **    .

Prim.cons.     DMHSVETRNYEAGKVARQQRKSPDERRRDDAAAEEARAANAARDAEEREAFKLRYETIDELRAAAF2ADP2S2D2CV2DIKAEHDAMVAAAKKRAADEAK

                  910       920       930       940       950       960       970       980       990      1000
                    |         |         |         |         |         |         |         |         |         |
Mipuc07g03770  DEASRSVPKPAELKRR-ILTPEERARVDKIDAPWRGVAKAYLEHDWPLPFWFSAGALAQGNETLELIVEQY-EIDTMDELGHWFGAMCSDASFDAK-SGR
Mipuc09g04130  DEASRSVPKPAELKRR-ILTPEERARVDKIDAPWRGVAKAYLEHDWPLPFWFSAGALAQGNETLELIVEQY-EIDTMDELGHWFGAMCSDAYFEAK-SGR
Mipuc01g03710  DRASRSVPKPAELKRR-TLKPAELDRVNKIDKPWRGVAKAYLEHDWPLPFWFSVGALAQGNETLELIVKEF-DIDTMDELGHWFGAMCSDAYFEAN-TGR
Mipuc03g05030  DEASRSVPKPAELKRRRLLKPAERARVDKIDAPWRGVAKAYLEHDWPLPFWFSAGALAQGNETLELIVEQY-KIDTLDELGHWFGAMCSAAYFEAN-TGR
Mipuc07g01720  DEASRSVPKPAELKRR-ILTPAERARVDKIDAPWRGVAKAYLKHDWPLPFWFSAGALAQGNETLELIVEQY-EIDTLDELGHWFGAMCSDAYFETN-TGR
Mipuc03g01960  DEASRSVPAPAELKRR-ILKLAERARVKKIDKPWRGVAKAFLEHDWPLPFWFSAGALAQGNETLELIVKEY-KIDTMDELGHWFGAMCSDAYFDAKSSGR
Mipuc01g06560  ---AVTMPPREALQILFHLIKGHLERLQVLAIPWRGIAMVSLQYKRPLPRCFPATQLALVIPAIANLLRDYPWIDTLADIGYFTGAMFSDAGMDCS--GN
Mipuc03g01120  ---AVTMPPREPLQILFHLSEGHLERLQVLAIPWRVIAMVSLRYKRPLPRCFPATQLALVIPAIANLLRDHPWIDTLADIGYFTGAMFSDAGMDCS--GN
                  : ::*     *:     *   .   *:. :   *** :* .  * .:.  ***  *..  **       ::  ::.:.   ***: ::*:: *** * *  :: .   *.

Prim.cons.     DEASRSVPKPAELKRRFIL2PAERARVDKIDAPWRGVAKAYLEHDWPLPFWFSAGALAQGNETLELIVEQYPEIDT2DELGHWFGAMCSDAYF2A2S2GR

                 1010      1020      1030      1040      1050      1060      1070      1080      1090      1100
                    |         |         |         |         |         |         |         |         |         |
Mipuc07g03770  FTLQTTNRDYADLQRANLHNVQWKTVPRP----GAEMESDFIEKFEATCSNGAKSWKYAITTTPVGDK--------------KLQEKFYTNRRGKKVILP
Mipuc09g04130  FTLQTTNREYADLQRANLHNVQWRTTTRP----GAEMESDFIEKFEATCSNGAKSWKYAITTTPVGDK--------------KLQEKFYTNRRGKKVILP
Mipuc01g03710  FALQTTNRDYADLQRANLHNVQWKTVPRP----GAEMESDFIEKFEATCSNGAKSWKYTIAATPVGDK--------------KLQEKFYTNRHGKKVILP
Mipuc03g05030  FALTTTNRDYADLQRANLHNVQWKTVPRT----GAEMESDFIEKFEATCSNGAKSWKYAITTTPVGDK--------------KLQEKFYTNRRGKKVILP
Mipuc07g01720  FALKTTNRDYADLQRANLHNVQWKTVPST----GGKMESEFIERFEATCSNGAKSWKYAITTTPVGDK--------------KLQEKFYTNRHGKKVILP
Mipuc03g01960  FALTTTNRDYADLQRANLHNVQWKTVPRP----GAEMESDFIEKFEATCSNGAKSWHYAIRTTPGGNK--------------KLQEKFYTNRHGKKVILP
Mipuc01g06560  FVLASTNREFPWRLRANLPGLNWNDGGDPDDRVSGRWDAEGVYVHTTKCSNGTPSEKHRVVSPPQSWV-----------HVWDWYPEMQCHQMGSKRLPR
Mipuc03g01120  FVLASTNREFPWRLRANLPGLNWNDGGDPDDRVSGRWDAEGVYVHTTKCSNGTPSEKHRVVSPPQSWVGGATKYSSRGSHVWDWYPEMQYHQMGSKRLPR
               *.* :***::.    **** .::*.      .     ... ::: :  . :.****: * :: : :.* .                 .   ::   :: *.* :

Prim.cons.     FALQTTNRDYADLQRANLHNVQWKTVPRPDDRVGAEMESDFIEKFEATCSNGAKSWKYAITTTPVGDKGGATKYSSRGSHVWKLQEKFYTNR2GKKVILP

                 1110      1120      1130      1140      1150      1160      1170      1180      1190      1200
                    |         |         |         |         |         |         |         |         |         |
Mipuc07g03770  KDLED---DPLFPLALTATPSWMDATVAGDGSLGEGIFSLAFCDFTSSELDWFINILARPRWRDG-TGAVVHGAFPVARKKKERKGQYT-LDMTEKSLKD
Mipuc09g04130  KDLED---DPLFPLALTATPAWMDATVAGDGSLREGIFSLAVCDFTPRELDWFINILARPRWRDG-TDTVVHGAFPVARRKKGRKGQYT-LDMTEKSLKD
Mipuc01g03710  KDLED---DPLFPLALTATPSWMDAIVAGDGSLREGIFSLAVCDFTPRELDWFINILARPRWRDG-TGAVVHGAFPVARKKKGREDQYT-LDMTEKSLKD
Mipuc03g05030  QNLED---DPLFPLALTATPSWMDAIVAGDGSLREGIFSLAFCDFTPRELDWFINILARPRWRDG-TGAVVHGAFPVERKKKGRKGQYT-LDMTEKSLED
Mipuc07g01720  QNLEK---DPLFPLALTATPSWMDATVAGDGSLGEGIFSLAFCDFTSSELDWFINILARPRWRDG-TDTVVHGAFPVVRKKTGRKGQYT-LDMTEKSLKD
Mipuc03g01960  KDLEK---DPLFPLALTATPAWMDAIVAGDGSLREGIFRLAFCDFTPRELDWFINILARPRWRDG-TDTVVHGAFPVVRKKTGRKGQYTTLDMTEKSLKD
Mipuc01g06560  SYVDDPLDREFMMSALTATPAAIDGLVVGDTTCDDTRVQHHLNDFTVREHEFISEWLGRVFWRDGTTGEIIHGGFPVRR-RHTRNDEHL-LEMYSDILEE
Mipuc03g01120  SYVDDPLDREFMMSALTATPAAIDGLVVGDTTCDDTRVLHHLNDFTVREHEFISEWLGRDFWRDGTTGEIIHGGFPVRR-KHIRKDQHV-LEMYSDILEE
               . ::.    ::  ******: :*. *.** :  :   .   . ***  * ::: : *.*  **** *. ::**.*** *  :   *:.::   *:*  .. *::

Prim.cons.     KDLEDPLDDPLFPLALTATP2WMDA2VAGDGSL2EGIFSLAFCDFTPRELDWFINILARPRWRDGTTG2VVHGAFPVARKKKGRKGQYTTLDMTEKSLKD

                 1210      1220      1230      1240      1250      1260      1270      1280      1290      1300
                    |         |         |         |         |         |         |         |         |         |
Mipuc07g03770  AEYAPRFGCGIRTHGIRVKEFIYTAALPVTRAYPHKGVFFPDNDLCSAAGAWRSRGESFTQIIVDG-VIRGFSLEELQAELLRATGLNFTAADLFGIISK
Mipuc09g04130  AGYAPRFGSGTRTHGIRVKEFIYTAALPVTRAYPHKGVFFPDNDLCSAAGAWRSRGESFTHIIVDG-VIRGFSLEELQAELLRATGLNFTDENLFGIITM
Mipuc01g03710  ARYAPRFGCGIRTHGIRVKEFIYTAALPVTRAYPHKGVFFPDNDLYIAGGAWRSRGESFTQIIVDG-VIRRFSLEEMQAELLRATGLNFTAKSLFGIITK
Mipuc03g05030  AGYAPRFGCGIRTHGIRVKEFIHTAALPVTRAYPHKGVFFLDNDLYSAALAWRSRGESFTQIIVDG-VIRGFSLEELQAELLRATGLNFTAADLFGIITK
Mipuc07g01720  AEYAPRFGCGTRTHGIRVKEFIYTAALPVTRAYPHKGVFFPDNDLCSAAGAWRSRGESFTHIIVDG-VIRGFSLEELQAELLRATGLNFTAADLFGIISK
Mipuc03g01960  AGYAPRFGCGIRTHGIRVKEFIDTAALPVTRAYPHKGVFFPDNDLYSAAGAWRSRGESFTQIIVDG-VIRGFSLEELQAELLRATGLNFTAADLFGIISK
Mipuc01g06560  NGYLALHSSTVRTHRNRLVDLLLTAPRALVLAYAHKVACVEDIDWMKRADEWRASGASYDHIVRELYLIRVFSFEEIQEAVQKHTKMLFDRRFLY-----
Mipuc03g01120  NGYLALHSSTVRTHRNRLVDLLLTEPRALVLAYAHKVACVEDIDWVKRADEWRASGASYDHIVRELYLIRVFSFEEIQEAVQKHTKMLFARQTLY-----
                 * . ...   ***  *: ::: *  . .:. **.**  .  * *     **: *  *: :*: :  :** **:**:*   : : * : *       *:

Prim.cons.     AGYAPRFGCGIRTHGIRVKEFIYTAALPVTRAYPHKGVFFPDNDL2SAAGAWRSRGESFT2IIVDGYVIRGFSLEELQAELLRATGLNFTAADLFGII2K

                 1310      1320      1330      1340      1350      1360      1370      1380      1390      1400
                    |         |         |         |         |         |         |         |         |         |
Mipuc07g03770  PLLPPGESSGRKKELGKHHGLGLPFPPRRTCDKTGNADHVKHTMHDVNAANVIIGIVRKGHEHAVLHGGLACGGEDSVKHFIKMRGAMYEEYGYIFDAKQ
Mipuc09g04130  PRLPPGESSGRKKELGKHHGLGLPFPPRRTCDKTGNSDHVKHTIHDVNAANVIIGIVRTGGRNAVLHGGLACGGEDSVKHFIKMRGAMYEEYGYIFDAKQ
Mipuc01g03710  PLLPPGDSSGRKKELGKHHGLGLPFPPRRTCDKTGNADRVKHTMHDVNAANVIIGIVRKGHEHAVLHGGLACGGEDSVKHFIKMRGAMYEESGYIIDAKQ
Mipuc03g05030  PLLPPGESSGRKKVLGKQYGLGLPFPPRRTCDKTGNADRVKHTIHDVNAANVIIGIVRKGHEHAVLHGGLACGGEDSVKHFIKMRGAMYEEYGYIFDAKQ
Mipuc07g01720  PLLPPGESSGRKKELGKHHGLGLPFPPRRTCDKTGNADHVKHTMHDVNAANVIIGIVRKGHEHAVLHGGLACGGEDSVKHFIKMRGAMYEEYGYIFDAKQ
Mipuc03g01960  PLLPPGESSGRKKVLGEHHGLGLPFPPRRTCDKTGNADRVKHTIHDVNAANVIIGIVRKGHEHAVLHGGLACGGEDSVKHFIKMRGAMYEEYGYIFDAKQ
Mipuc01g06560  ------------EKLGS---LGLVRPAHRTCEKTGNDERTKLNRCNVRDVNVVIQKVREGGNDAVSFGGLDEGGEASIQHWLKWIAPVFERQGYVIDHKR
Mipuc03g01120  ------------EKLGS---LGLVRPAHRTCEKTGNDERTKLNRCNVRDVNVVIQKVREGGNDAVSFGGLDEGGEASIQHWLKWIAPVFERQGYVIDHKR
                           : **.    *** *.:***:**** ::.*  .  :*. .**:*  ** *  .** .***  *** *::*::*  ..::*. **::* *:

Prim.cons.     PLLPPGESSGRKKELGKHHGLGLPFPPRRTCDKTGNADRVKHT2HDVNAANVIIGIVRKGHEHAVLHGGLACGGEDSVKHFIKMRGAMYEEYGYIFDAKQ

                 1410      1420      1430      1440      1450      1460      1470      1480      1490      1500
                    |         |         |         |         |         |         |         |         |         |
Mipuc07g03770  GLCIFPGGTIDRDTTKLLTQEVLDAQQRYHTGCVENGKFTAPELLPRRTDAQLKVQFAPLLEEKLEVRALIVKLQGWSRELRRPASSNKARTYFIYTDP-
Mipuc09g04130  GLCIFPGGTIDRDTTKLLTQEVLDAQERYHKGCVENGKFTPPELLPHRTDAQLKVQFAPLLEEKLEVRALIVKLQGWRMELRRTASSNWARSYFIYTDPG
Mipuc01g03710  GLCIFPGGTIDRDTTKLLTQEVLDAQQRYHKGCVENGKFTPPELLPHRTDAQLKVQFAPLLEEKLEVRALIVKLQGWRMELRRTASSNRARSYFIYTDP-
Mipuc03g05030  GLCIFPGGTIDRDTTKLLTQEVLDAQERYHTGCVENGKFTPPELLPHRTDAQLKVQFAPLLEEKLRLSALIVKLQGWRMELRRTASSNRARSYFIYTDP-
Mipuc07g01720  GLCIFPGGTIDRDTTKLLTQEVLDAQQRYHTGCVENGKFTAPELLPRRTDAQLKVQFAPLLEEKLRLSALIVKLQGWRMELRRTASSNKARSYFIYTDP-
Mipuc03g01960  GLCIFPGGTIDRDTTKLLTQEVLDAQQRYHKGCVENGKFTPPELLPHRTDAQLKVQFAPLLEEKLRLSALIVKLQGWRIELRRTASSNRARSYFIYTDP-
Mipuc01g06560  YRAIVPSGMIG--SKTDWTEEEIRDQELFVVHCKTFENNMPP-LLELPGEAEHREHFKNWISAPEKSDCAKLVSLPYWIRARRDTNTTKLGFQCTLPDG-
Mipuc03g01120  YRAIVPSGMIG--STTDWTEEEIRDQELFVVHCKTCENNMPP-LLELPGEAEHREHFKNWISAPEKSYCAKLVSLPYWIRVRRDTNISELGFQCTFPDG-
                .*.*.* *.   :..   *:* :   *: :      *      :  .* **       :*: : :*   *:.      .   :  . ** :. .          .*

Prim.cons.     GLCIFPGGTIDRDTTKLLTQEVLDAQ2RYH2GCVENGKFTPPELLPHRTDAQLKVQFAPLLEEKL222ALIVKLQGWRMELRRTASSN2ARSYFIYTDPG
```

```
                  1510      1520      1530      1540      1550      1560      1570      1580      1590      1600
                     |         |         |         |         |         |         |         |         |         |
Mipuc07g03770    -------------------------------------------------------------------------------------------GTGTVKKET
Mipuc09g04130    TGTVKKETPDLNSAMKWIVVNQPENAQQLKDLLTKYQPEIKKQFERTATATATATATATVLIVKLQGWRMELRRTASSNQARSKYFIYTDPGTGTVKKET
Mipuc01g03710    -------------------------------------------------------------------------------------------GTGTVKKET
Mipuc03g05030    -------------------------------------------------------------------------------------------GTGTVKKET
Mipuc07g01720    -------------------------------------------------------------------------------------------GTGTVKKET
Mipuc03g01960    -------------------------------------------------------------------------------------------GTGTVKKET
Mipuc01g06560    ----------------------------------------------------------------------------------------------TTKSTV
Mipuc03g01120    ----------------------------------------------------------------------------------------------TTKSTV
                                                                                                              *.*. .
Prim.cons.       TGTVKKETPDLNSAMKWIVVNQPENAQQLKDLLTKYQPEIKKQFERTATATATATATATVLIVKLQGWRMELRRTASSNQARSKYFIYTDPGTGTVKKET

                  1610      1620      1630      1640      1650      1660      1670      1680      1690      1700
                     |         |         |         |         |         |         |         |         |         |
Mipuc07g03770    RDLN-------------------------------------------------------------------------------------------SAIRW
Mipuc09g04130    RDLN-------------------------------------------------------------------------------------------SAIKW
Mipuc01g03710    PDLNR------------------------------------------------------------------------------------------MAIKW
Mipuc03g05030    LDLN------------------------------------------------------------------------------------------GMAIKW
Mipuc07g01720    RDLN------------------------------------------------------------------------------------------RKAIKW
Mipuc03g01960    LDLNFAIKWIIANQPENAQQLKDLLTKYQPEIAEQFKRTATATATATATATVLIVKLQGWRMELRRTASSNQARSYFIYTDPGTGTVKKETPDLNSAIKW
Mipuc01g06560    FQT-------------------------------------------------------------------------------------------------
Mipuc03g01120    FQN-------------------------------------------------------------------------------------------------
                  :
Prim.cons.       RDLN2AIKWIIANQPENAQQLKDLLTKYQPEIAEQFKRTATATATATATATVLIVKLQGWRMELRRTASSNQARSYFIYTDPGTGTVKKETPDL3SAIKW

                  1710      1720      1730      1740      1750      1760      1770      1780
                     |         |         |         |         |         |         |         |
Mipuc07g03770    IIANQPENAQKFKDSLTKYQPEIAEQFEWTATATATA--------TATATVPAGGAEGDGDDAEGARDEDEIALTQPKKKRKTKR
Mipuc09g04130    IVVNQPENAQKFKDSLTKYQPEIKKQFEWTATATATA--------TATVPVPAGGAEGDGDDAEGARDEDEIALTQSKKKRKTKR
Mipuc01g03710    IFANQPENAQQLEDLLTKYQPEIAEQFKRTATATVTASATATVTATATVPVPAGGAEGDGDDAEGARDEDEIALTQSKKKRKTKR
Mipuc03g05030    IIANQPENAQQLKDLLTKYQPEIKKQFEWTATATATA--------TATTTVPAGGAEGDGDDAEGARDEDEIALTQSKKKRKTKR
Mipuc07g01720    IIANQPENAQQLKDLLTKYQPEIAEQFEWTATATATA--------TATATVPAGGAEGDGDDAEGARDEDEIALTQPKKKRKTKR
Mipuc03g01960    IIANQPENAQKFKDSLTKYQPEIKKQFEWTATATATA--------TATATVPAGGAEGDGDDAEGARDEDEIALTQPKKKRKTKR
Mipuc01g06560    ----------------------VKRALRFVRSEKYPN--------------------------EYVQEAQQLGLRGQ--------
Mipuc03g01120    ----------------------VQRALHFVRSKKYPN--------------------------EYVQEAQQLGLRGQ--------
```

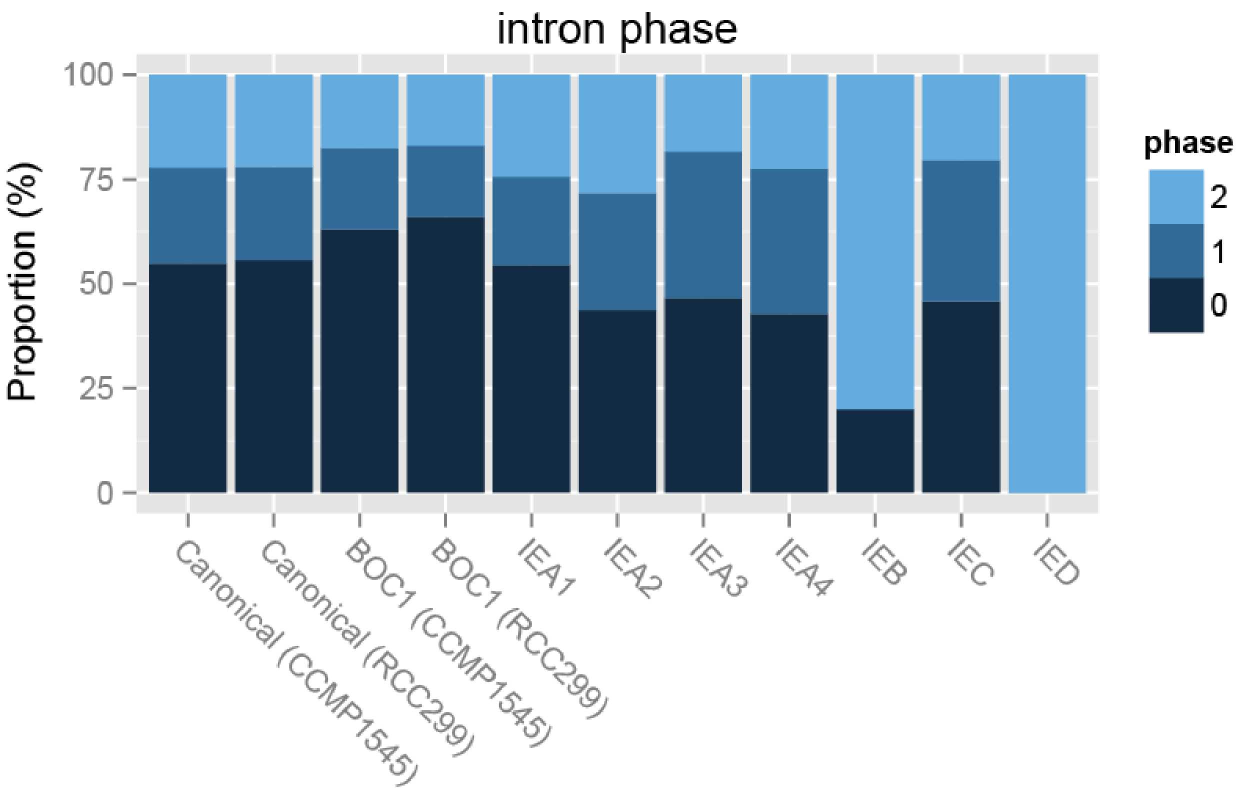

Figure S10. Phase distribution of Introner Elements, BOC1 and canonical introns.

```
                 3’ ccgUCCAUUCAua 5’   U1 snRNA
                       :::.::: :
Mipur01i11380   gacacaaccgGTGAGTTTATT-----TTAATCT---T----TG----TATGGACAATTAGACTGACGTG---GTTTTTTT------TAACAGgtccttactt
Mipur16i00160   gcgtacctcgGTGAGTTTATT-----TTAATTTTGT----TG----TATGGACAATTTGACTGACGTG---GTTTTTTT--TTA---ACAGccccgctcat
Mipur02i00250   gtacaacacgGTGAGTTTATTTACACTTAATCT---T----TG----TATGGACAATTAGTCTGACGTG---GTTTTTTA------AAACAGgaagcgtacg
Mipur07i02450   gtaccggacgGTTCGTTTATT-----TTAATCT---T----TG----TATGGGCAATTAGACTGACGTG---GTTTTTTT--TTA---ACAGgaaccggcga
Mipur09i02530   tcgagttgagGTGAGTTTATT-----TTTATTT---T----CG----TATGGGCAATTAGACTGACGTG---GTTTTTTG--TTT---ACAGggcgtacaaa
Mipur05i00180   ctctgtacagGTGAGTTTATT-----TTTATTT---T----CG----TATGGGCAATTCGACTGACGTG---CTTTTTTG--TTA---ACAGgttccccccc
Mipur01i11610   ggtcatggtgGTGAGTTTATT-----TTAATCT---TCGTACG----TATGGGCAATTAGACTGACGTG---GTTTTTTT--GTTA--ACAGgggttaccgg
Mipur01i11430   cgactcgccgGTGAGTTTATT-----TTCATTT---TCGTATG----TATGGGCAATTTGTATGACGTG---GTTTTTTT--TTA---ACAGggcggaggcg
Mipur01i11510   acgcaacgcgGTACGTTTATT-----TTAATCT---TTGTAAGATCTTATGGTCAATTAGAATGACGTG---GTTTTTTA--TTT---ACAGgtgcgcgtgg
Mipur14i00280   tcgtgcgcagGTGAGTTCATTGTAGTTTAATCT---T----TG----TATGGGCAAATCGACTGACGTG---GTTTTTCA------AAACAGgtacccgtcc
Mipur01i11240   cgcggaatcgGTGAGTTTATT-----TTAATCT---TTGTATG----TATGGGCAATTTGTATGACATG--TGTTTTTTT--TAAA--ACAGgatcgaaaag
Mipur01i11320   ggcgcggctgGTGAGTTTATTTT---TTTATTT---TCGTATG----TATGGGCAATTAGTATGACGTG------TTTTT--TTAAAACAGgacccggcgg
Mipur02i00320   gggcgacaagGTGAGTTTATT-----TTAATCATCATATCCCG----TATGGGTAATTAGAATGACGTG---GTTTTTTTTTTTT---ACAGgttgaggagg
Mipur16i00950   tcggcgattgGTGCGTTTATT-----TTCATTT---T----CG----TATGGGCAATTTGACTGACGTG---GTTTTTTG--TGTACTACAGgacccggatg
Mipur16i00940   agaagctacgGTGCGTTTATTT----TTTATCC----CTTCTG----CATGGGCAATTAGAATGATGTG---GTTTTTTTTGTTTACCACAGggggcatgat
Mipur09i02370   gcggcaactgGTGAGTTTATT-----TTAATCT---T----TG----TATAGGCAATTTGACTGACGTG-ACGTGTTTTTTATTC---TCAGgcaacgcgac
Mipur01i10880   aatgatccggGTGAGTTTATT-----TTCATTT---T----CG----TATGGGCAATTAGAGTGACGTG---GTTTTGTT--TTAA--ACAGgaggaggatt
Mipur14i00290   ctcgtgaacgGTGAGTTTATT-----TTCATTT---T----CG----TATGGGCAATTAAAATGACGTG---GCTTTTTA------AAACAGggtacgccgt
Mipur16i00410   gtcgtcaccgGTGAGTTTATT-----TTCATTT---T----CG----TGTGGGCAATTAGAATGACGTG---GTTTTTTT------CAACAGgcatgacgcc
Mipur09i02260   gacgcacgagGTGAGTTTATT-----TTCATTT---ATTTTCG----TATGGGCAATTAGAATGACGTG---GTTTTTTT--------TTAGgacgccttcc
Mipur04i01040   gcgacctgagGTGAGTTTATT-----TTAATCT---T----CG----TATGGGCAAGCGGACTGACGTGTTTATTTTCTACCTTCGTAACAGggatcactct
Mipur03i03280   atgttcaccgGTGAGTTTAT------TTAATCT---T----TG----CGTGGGCGATTCGTCTGACGTG---GTTTTTTG--TTGTACACAGgcgccgagcc
Mipur08i01840   tggcacgcggGTGCGTTCTTT-----TTTTTGT---TTTTTCG----TATGGGCAATGAGACTGACGTGTACGTGTTTTG--TTT---ACAGggatggtcgc
                                                                 :::.  :..
                                                            3’ uAUGAU^Gug  5’   branchpoint
```

Figure S11. Alignment of typical IE-C sequences, flanked by their exonic regions (grey). Base-pairing information regarding the donor site (U1 snRNA) and the branch-point (U2 snRNA) is also provided.

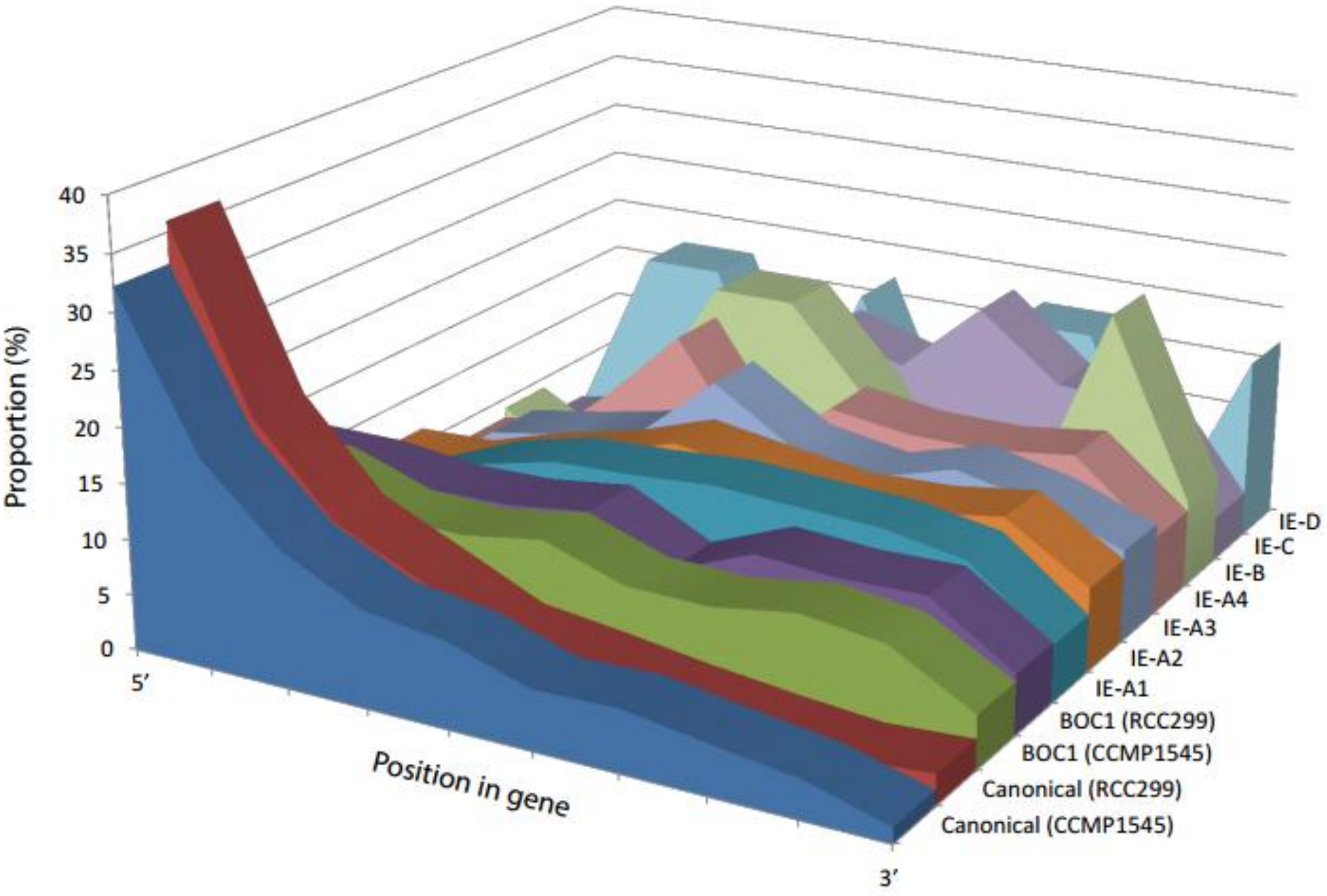

Figure S12. Positioning of Introner Elements, BOC1 and canonical introns inside genes.

Figure S13. Presence/absence polymorphisms in *Micromonas*: IE-A1 PAP (a), IE-A2 PAP (b), IE-A3 PAP (c), and two IE-C PAPs (d).

a)

CCMP1545: scaffold_14 (384641..384976)
Metagenomic read: CAM_READ_0212050959 (CAMERA accession)

```
  CCMP1545 GCGGCTGCTGCAGTACGCGTTCAGCGCGTTCAGCGCGTGCGACCGGTTCTGCGCCGTCGCGAGCGAGGACGACAAGGCGACGCTGCTGG
           ||||||||||||||||||||||||||||||||||||||||||| |||||||||| || |||||||||||||||||||||||| ||||||
Metagenome GCGGCTGCTGCAGTACGCGTTCAGCGCGTTCAGCGCGTGCGACGGGTTCTGCGCGGTGGCGAGCGAGGACGACAAGGCGACGTTGCTGG

ACGCATTTTTTCGAGCCGATGCGGCGGGCGGCGGCGAGAGCGCCGCCGCTCCGGCGTCGCG---------------------------------------
||||| |||||||||||||||||||||||||||||||||||||||||||| | || |
ACGCA-TTTTTCGAGCCGATGCGGCGGGCGGCGGCGAGAGCGCCGCCGCTCCGGAGGCGAGGTGCGTTTTATCTCACACTGGTTCCCATACGACGGCGTT

----------------------------------------------------------------------------------------------------
                                         IE-A1 PAP
CGCGTGGTGAACGCCGATCCTTAAGGACTTTGCCCGGCGCATCTCTCCGCCCACACCTCGCTTTCAATCCCCGACCTCGACGCCTTTCAACTCCATCTGA

----------------------------------GACGAAGAGATATGTCGGACGCGCGTCGGACGAACCTCGCGACGAAAGCCGCCGTCGGTGGGACG
                                  |||||||||||||||||||||||||||||||||||||||||||| ||||| |||||||||||||
CGCCTTTCAACTCCACCCCGACATCGCTTCGTAGGACGAAGAGATATGTCGGACGCGCGTCGGACGAACCTCGCGACGAAGAGCCGTCGTCGGTGGGACG

AGCACGAGGGGTTCGGCCCGCAGCGTGGATCGAGGCCGACGTCCGAGCACGACGTCGTCGTGAAGGGATTGATCGGGTTGGGGTTGGAGGAGCCGTGGAG
||||||||||||||||||||||||||||||||||||||||||||||||||||||||||||||||||||||||||||||| ||||||||||||||||||||
AGCACGAGGGGTTCGGCCCGCAGCGTGGATCGAGGCCGACGTCCGAGCACGACGTCGTCGTGAAGGGATTGATCGGGTT-GGGTTGGAGGAGCCGTGGAG

TTCGTGGGA
|||||||||
TTCGTGGGA
```

b)

CCMP1545: scaffold_01 (251138..251395)
Metagenomic read: CAM_READ_0212289655 (CAMERA accession)

```
  CCMP1545 CGGCGAGCGGCTGAGCGAGCTCGACCCGGACACGTTCGAGTTCGAGGCGAGGAAAATTCTCAG--------------------------
           |||||||||| || |||||||||||||||||||||||||||||||||||||||||||||||||
Metagenome CGGCGAGCGGTTGGGCGAGCTCGACCCGGACACGTTCGAGTTCGAGGCGAGGAAAATTCTCAGGTGCGGTCGACGCGAAACAAACGTCG

-------------------------------------------------------------------------------------------CGGCCTCGG
                                  IE-A2 PAP                                                ||||||||| 
CCGCACCGCTCGGATTCAACATTTGATCGCGTGGGTCCCTTTCAACTGACCGGTGTACTATCCTATTTTTGTACGGAACGCCCCTCATCAGCGGCCTCGG

GTTTTCGAGCGCCACGGTGCCGATGGAACGCGCGACGAAGGACATGTCCGGCGGGTGGCGCATGCGCGTCTCGCTCGCGAAGGCGCTGTTCGCCGCGCCG
| |||| |||||||| |||||||||||||||||||||||||||||||||||||||||||||||||||||||||||||||| |||||||||||||||||||
G-TTTCCAGCGCCACCGTGCCGATGGAACGCGCGACGAAGGACATGTCCGGCGGGTGGCGCATGCGCGTCTCGCTCGCGAAAGCGCTGTTCGCCGCGCCG

ACGCTGCTGCTTCTGGACGAGCCGACGAACCATCTCGACCTCGAGGCGTGCGTGTGGCTCGAAGAACATCTGTCGCGGTATAAAAA-
||||||||||||||||||||||||||||||||||||||||||||||||||||||||||||||||||||||||||||||||||| ||
ACGCTGCTGCTTCTGGACGAGCCGACGAACCATCTCGACCTCGAGGCGTGCGTGTGGCTCGAAGAACATCTGTCGCGGTATAAGAAG
```

c)

CCMP1545: scaffold_14 (643394..644184)
Metagenomic read: AACY020815663 (GenBank Accession)

```
  CCMP1545 GTCGAGTTCCGAACATGGGCGACCTCGCGGACGTCAGCGAGCTCCTCACGAGCAAAGGTTACGGCAGCGACCACAGCGACAGCGAGGGC
           |||||||||||||||||||||||||||||||||||||||||||||||||||||||||||||||||||||||||||||||||||||||||
Metagenome GTCGAGTTCCGAACATGGGCGACCTCGCGGACGTCAGCGAGCTCCTCACGAGCAAAGGTTACGGCAGCGACCACAGCGACAGCGAGGGC

GAACGCGCGCGTGGACTTGACGCAAGATTACAACCGTGTCGCGCGCGAAGGAACGCGGTCGCGGATCATCCTGCAGGAGATTGGCCCGCGGATGGAGCTC
|||||||||||||||||||||||||||||||||||| |||||||||||||||||||||||||||||||||||||||||||||||||||||||||||||||
GAACGCGCGCGTGGACTTGACGCAAGATTACAACCGCGTCGCGCGCGAAGGAACGCGGTCGCGGATCATCCTGCAGGAGATTGGCCCGCGGATGGAGCTC

GAGCTCGTGAAGGTGGAGGAGGGGATGTGCGAGGGGCGGGTGTTGTATCACGCGTACGTGAAGAAGACGGAGGAGGAGGTGTTGGAG-------------
|||||||||||||||||||||||||||||||||||||||||||||||||||||||||||||||||||||||||||||||||||||||
GAGCTCGTGAAGGTGGAGGAGGGGATGTGCGAGGGGCGGGTGTTGTATCACGCGTACGTGAAGAAGACGGAGGAGGAGGTGTTGGAGGTGAGACTGCTTC

----------------------------------------------------------------------------------------------------
                                         IE-A3 PAP
CCATACGACCCCGTTCGCGTGGTGCACGCCGTTCCTTGAGGACTTTTCCCGTCGTCACTCTTCACCCGCGCTTCCCTTTCAACGTTTGACCGGTAAGACG

------------------------------CTGGAGAATAAGGCGGTGACGAAGGAGGCGCTGCGGAAGCGACGGAGGGAGGAACAGGAGGCGAACGTGC
                               |||||||||||||||||||||||||||||||||||||||||||||||||||||||||||||||||||||
TTCGACTGACCGATCGCTTCACCCACGCAGTTGGAGAATAAGGCGGTGACGAAGGAGGCGCTGCGGAAGCGACGGAGGGAGGAACAGGAGGCGAACGTGC

GGAGGAAGGAGAGAGAGGCGAAGGAGAAGGCGGCGATGGAGGAAGAACTCGCGGGGATGAAGAAGAGGAAGCGCGATAATAAATCCGCCCGGGATAAGCT
||||||||||||||||||||||||||||||||||||||||||||||||||||||||||||||||||||||||||||||||||||||||||||||||||||
GGAGGAAGGAGAGAGAGGCGAAGGAGAAGGCGGCGATGGAGGAAGAACTCGCGGGGATGAAGAAGAGGAAGCGCGATAATAAATCCGCCCGGGATAAGCT
```

```
CGACGAGAACGGACGGAAGAAAAAGAAGGGGGCGAGCGGCGCGGAGAAGAGACGCGACGGCGGCGACGACGACGAGGCGCCGTTTAAGCGCGGTCGAAAG
||||||||||||||||||||||||||||||| ||||||||||||||||||||||||||||||||||||||||||||||||||||||||||||||||||||
CGACGAGAACGGACGGAAGAAAAAGAAGGGGCCGAGCGGCGCGGAGAAGAGACGCGACGGCGGCGACGACGACGAGGCGCCGTTTAAGCGCGGTCGAAAG

AGTCAGTACAAGGACAGGGAAG------------------GCGGCGGCGGCGGCGGAAGCGGCGGCGGCGGGAAGAAATCGCCGGCGGGGACGAAGGGCA
||||||||||||||||||||||                  ||||||||||||||||||||||||||||||||||||||||||||||||||||||||||||
AGTCAGTACAAGGACAGGGAAGGCGGCGGCGGCGGAAGCGGCGGCGGCGGCGGAAGCGGCGGCGGCGGGAAGAAATCGCCGGCGGGGACGAAGGGCA

AGGACAAGAACAAGCGCCCGGGGAAGGGTGCGCGCGCGAAGAGGAAGTAGAGACACACGCTCGGCCACGCGGTACGCGCGACGTCTCGCGTCTCGCTCGC
||||||||||||||||||||||||||||||||||||||||||||||||||||||||||||||||||||||||||||||||||||||||||||||||||||
AGGACAAGAACAAGCGCCCGGGGAAGGGTGCGCGCGCGAAGAGGAAGTAGAGACACACGCTCGGCCACGCGGTACGCGCGACGTCTCGCGTCTCGCTCGC

GTCTTCAAAAGTTGTCGGGATTGTACATCCGCATCGCCCCCGTCCGGAGCAA
||||||||||||||||||||||||||||||||||||||||||||||||||||
GTCTTCAAAAGTTGTCGGGATTGTACATCCGCATCGCCCCCGTCCGGAGCAA
```

d)

RCC299: chrom_06 (160647..160930)
Metagenomic read: CCMP1764_READ_00758094 (CAMERA accession)

```
    RCC299 CTCATCGACGTTCGGTGGGATCGGTGGCGAACCGACGCCGCGGGACGGTACCTGGTCAGCCCGCGCGTCGTGTCGCACCTCGCCAACTG
           |||||||||||||||||||||||||||||||||||||||||||||||||||||||||| ||| ||||||||||||||||||||||||||
Metagenome CTCATCGACGTTCGGTGGGATCGGTGGCGAACCGACGCCGCGGGACGGTACCTGGTCA-CCCACGCGTCGTGTCGCACCTCGCCAACTG

CCACCCGCGGGCTGAGTTCGGGCAGGTGATTCTTTGCATTTTAATCTTTGCATCATGGGCAATCAGATTGACGTGGTTTTTTATTCGCAGGCTGTGCGGC
 |||||||||||||||   |||||                              IE-C PAP                             |||||||||
TCACCCGCGGGCTGAGCGTCGGCAA-----------------------------------------------------------------ACTGTGCGGC

GCCCGAGCCGTCGCCGTCGTCGACGAGACG----------------------------------------------------------GCGTACGTCGTG
|| |||||||||||||||||||||||||||                        IE-C PAP                          ||||||||||||
GCTCGAGCCGTCGCCGTCGTCGACGAGACGGTGAGTTTATTTGTTATTTCGTATGGGCAATTAGTTTGACATCGTGTTTTGTTCACAGGCGTACGTCGTG

TCGGAGATGTCCAACTCCCTCTCCGTCGTCGTCCTCCCGGCG
|| |||||||| ||||||||||||||||||||| |||| |||
TCCGAGATGTCAAACTCCCTCTCCGTCGTCGTCGTCCCCGCG
```

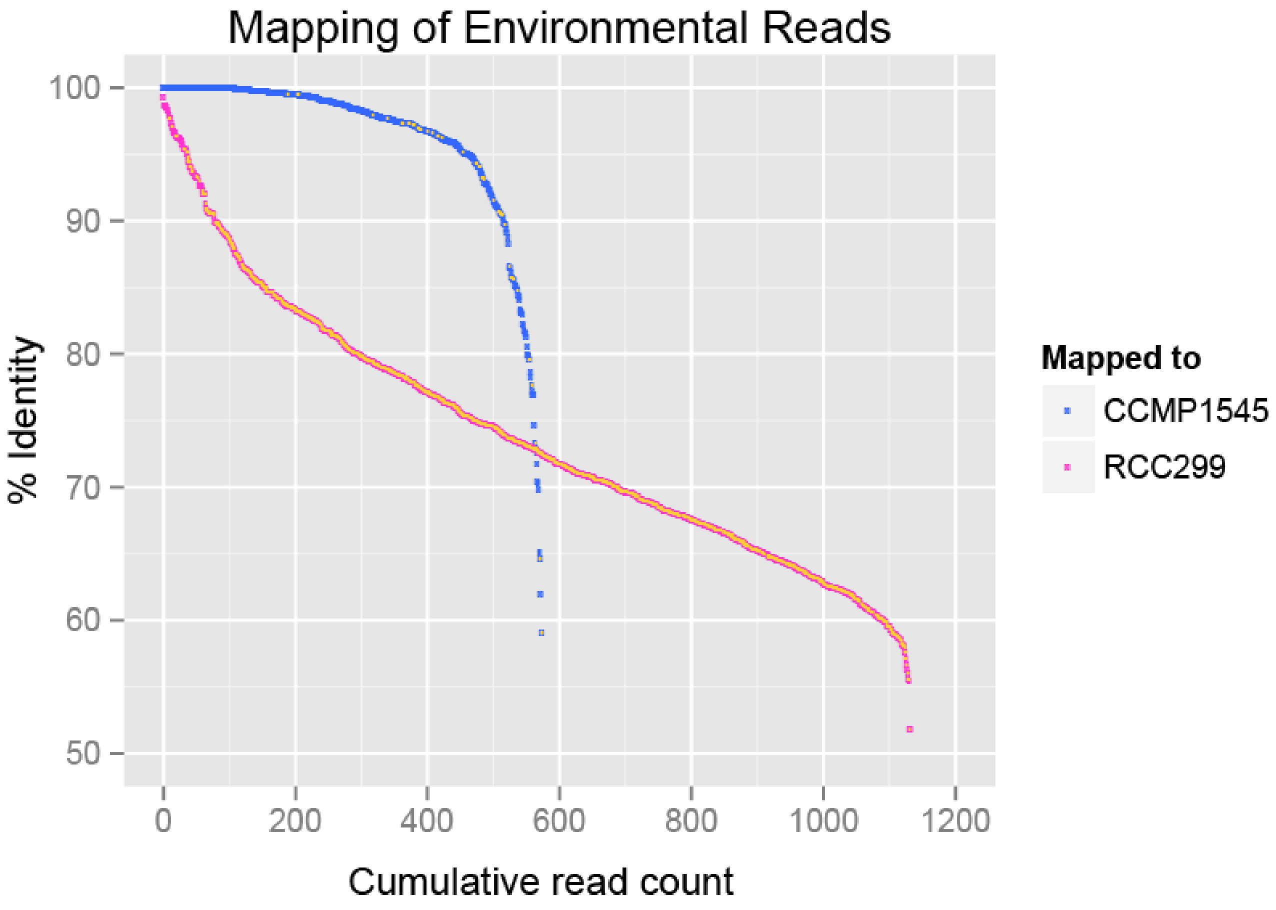

Figure S14. Mapping identities for environmental sequences. Yellow dots indicate alignments showing PAPs.

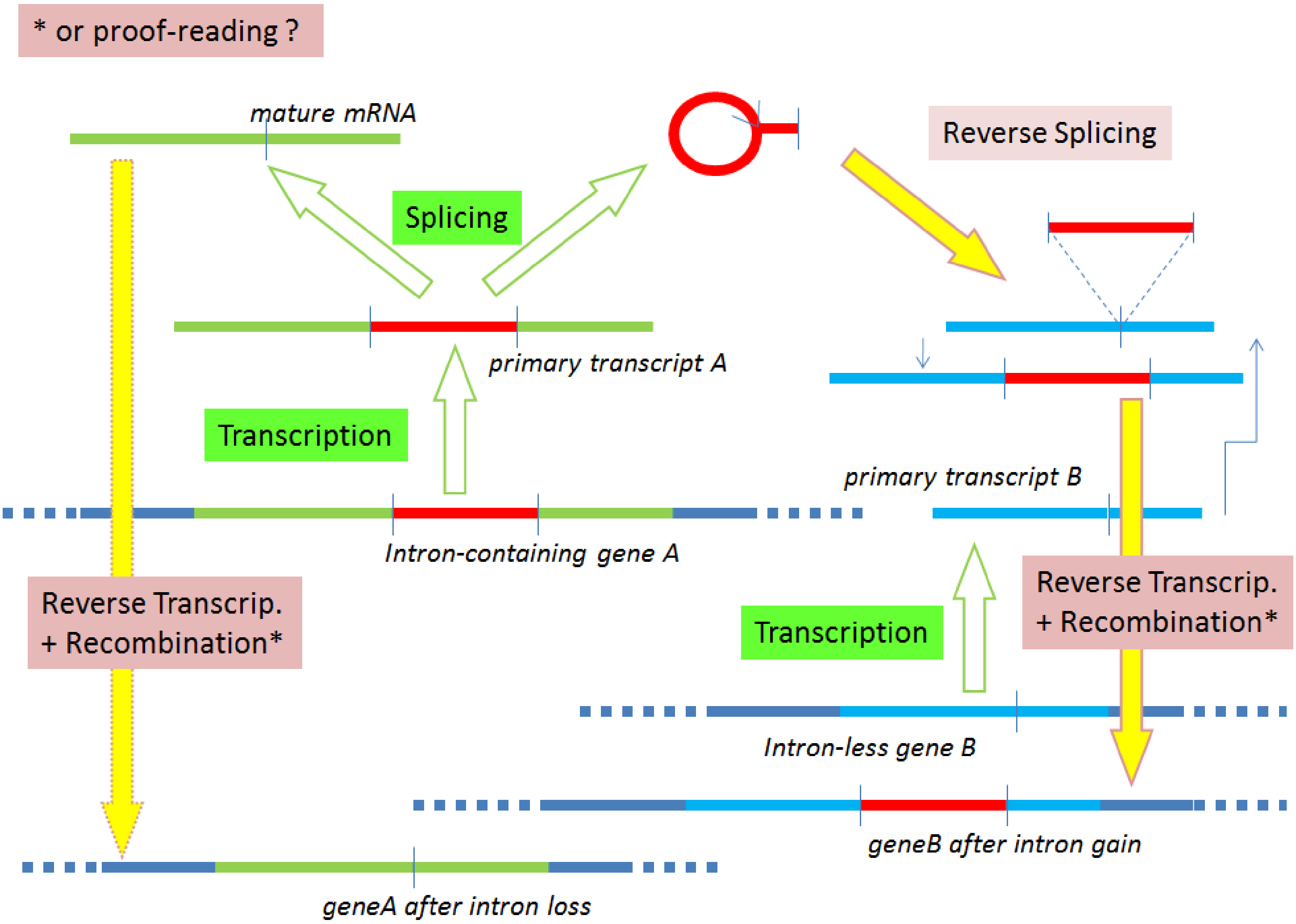

Figure S15. Introner Element replication mechanism.

Mipuc05i01435: IE-A1 + IE-A1

GCGCGTTCTGACTGGTCCCCATACGACCGCGTTCGCGTGGTGAACGCCGATCCTTAAGGACTTTTGC
CGTCCTCTCTCCCCGCCCACCCCTTCCTTTCAATCCCCGCCCGCGACGCCTTTCAACTCCTTTCAAC
TCCTCACGCCGGTCCCCGTACGACCCCGTCGGCGCGGTGCACGCCGTTCCTTAAGGACTTTCTCTCC
CGGCGTGCGTTCGTCTCTCTCCGCCCAGGGTCCTTCGGTTCCAATCCCGACGCGCCTCGACGCCTTT
CAACTCCAACAACTGACGCCTTTCAACTTCACCCCGACGCCTTTCAACTTCACCCCCGTCAG

Mipuc02i02490: IE-A1 + IE-A2

GCGCGTTCTTCCTCACCGCGCCGGTCCCCGCGCTGACCGGTCCCCGCGCTGACTGGTCCCCGTACGA
CCGCGTTCGCGTCGTGAACTTCATTCCTCAAGGACTTTCGGCCGATCCTCGCGGACGGACTTCGCCC
GCCCGGCGCGTCTCTCCGCGCACCCCTCGCCGCTTTCGATCCCCCCGACGCGCCTACGCGACGCCTT
TCGACTCCATCTGACGCGCCCTTGAACTCCACCCCGACGCGCTTCGCTTCGCTTCGCTTCGCGCGGA
CCCTCCCTCGACCCTCAGGTGCGTTCTATACAAAAGTTTTTCACCCACCGCTCGGTATCAACATTTG
ATCGCGTGGGTCCTTTTCAACTGACCGGTGAACTATTTTTGTATGGAACGACCCTCAG

Mipuc16i00710: IE-A1 + IE-A3

GCGCGTTCTGTCTCATCACACTGGTCCCCGTACGACCGCGTTGGCGCGGTGAACGCCGTTCCTTAAG
GACTTTGCCCGCCCGCGTGCGTTTCTCTCCGCCCACACCACGGTTTCAATCCCGACAACACACCGCG
ATGCCTTTCAACTTCAACCGACGCCTTTCAACTCCACCCCGACGTTCATAACTACCCTAAACCCCGT
TCGCGTGGTGAACGCCGTTCCTTAAGGACTTTTCCCGTCGTCACTCTTCACCCGCGCTTCCCTTTCA
ACGTTTGACCGGTAAGACGTTCGACTGACCGATCGCTTCACCCACGCAGGCTCGCGTCGCTCTCCGC
GACGTCGAAG

Mipuc12i00190: IE-A? + IE-A1

GCGCGTTTGGGGCTGACTGGTCCCCATACGACCGCGTTCGCGTGGTGAACGCCGATCCTTAAGGACT
TTACTTCCCGGCCGCCTCCGCCTCCGTCCTATCATCACGCCGGTCCCCGTGCGACTCAGTCGGCGCG
GTGAACGCCGTTCCTCGAGGACTTTGCCCCCCGCTCGCGTTTCTCTCCGCCCATCGCCCCTCGGTTT
TAATCCCGACGCACCGCGACGCCTCGCGACTTCATCTGACACCCCCTGAACGCCACCCACCCCGACG
TCACCTCGTATGGAAAACGACCCTCAG

Figure S16. Examples of merged Introner Elements. Due to loss of internal splice structures when merging, it is hard to exactly delineate borders.

CCMP1545: scaffold_14; 341989..343470
Metagenomic read: AACY02323272 (GenBank Accession)

```
   CCMP1545 CGACGCGCGCGTGGCTGCGTCGACTCGTGGACCCGACGTCCTCGCTTCA
            |||||||||||||||||||||||||||||||||||||||||||||||||
Metagenome CGACGCGCGCGTGGCTGCGTCGACTCGTGGACCCGACGTCCTCGCTTCA

CGCGAACGGCGCGAATTTCCTCGAACACTATCACGCGCTCGTCGACCGCGTCGACGTCGA
||||||||||||||||||||||||||||||||||||||||||||||||||||||||||||
CGCGAACGGCGCGAATTTCCTCGAACACTATCACGCGCTCGTCGACCGCGTCGACGTCGA
               Pre-existing IE-A1
CGTCGAGACGCTGAGGCGCGTTCTGACTGGTCCCCATACGACCGCGTTCGCGTGGTGAAC
|||||||||||||||||||||||||
CGTCGAGACGCTGAGGCGCGTTCTGA-----------------------------------

GCCGATCCTTAAGGACTTTTGCCGTCCTCTCTCCCCGCCCACCCCTTCCTTTCAATCCCC
                       IE-A1 Insertion
------------------------------------------------------------

GCCCGCGACGCCTTTCAACTCCTTTCA-ACTCCTCACGCCGGTCCCCGTACGACCCCGTC
                       |||| |||         ||||||| ||||||||||||
-----------------------TTCACACT---------GGTCCCCATACGACCCCGTC

GGCGCGGTGCACGCCGTTCCTTAAGGACTTTCTCTCCCGG----CGTGCGTTCGTCTCTC
|||| |||| ||  | ||||||||||||||||||||||||    |||  ||||||||||||
GGCGTGGTGAACTTCATTCCTTAAGGACTTTCTCTCCCGGCGTTCGTTCGTTCGTCTCTC

TCCGCCCAGGGTCCTTCGGTTCCAATCCCGACGCGCCTCGACGCCTTTCAACTCCAACAA
| |||||||||||| |||||| | |||||||||||||||||||||||||||||||  |
TGCGCCCAGGGTCCCTCGGTTTCGATCCCGACGCGCCTCGACGCCTTTCAACTCC-GCTT

CTGACGCCTTTCAACTTCACCCCGACGCCTTTCAACTTCACCCCCGTCAGCGCGTTGTAC
||||||||||||||||||||||||||||||||||||||||||||||||||||||||||||
CTGACGCCTTTCAACTTCACCCCGACGCCTTTCAACTTCACCCCCGTCAGCGCGTTGTAC

CGCGACGTGAGCTGCGTGACCACCGCGCAGGGGGAGAAAGTCCGCGGCGGGAGAGACATC
||||||||||||||||||||||||||||||||||||||||||||||||||||||||||||
CGCGACGTGAGCTGCGTGACCACCGCGCAGGGGGAGAAAGTCCGCGGCGGGAGAGACATC

TCCGCGCTCCTCGCGTCGCGCGCCGCCTCCGGCGCGGGCGCGCTGCGGCGGACCGTGCGG
||||||||||||||||||||||||||||||||||||||||||||||||||||||||||||
TCCGCGCTCCTCGCGTCGCGCGCCGCCTCCGGCGCGGGCGCGCTGCGGCGGACCGTGCGG

ACGATCGACGCGCTGCCCGGCGGCGTCGACGGCGGCGTGACCGTGTTCGCGACCGGGACG
||||||||||||||||||||||||||||||||||||||||||||||||||||||||||||
ACGATCGACGCGCTGCCCGGCGGCGTCGACGGCGGCGTGACCGTGTTCGCGACCGGGACG
```

Figure S17. Examples of merged Introner Elements. Due to loss of internal splice structures when merging, it is hard to exactly delineate borders.
